# Mechanisms linking cytoplasmic decay of translation-defective mRNA to transcriptional adaptation

**DOI:** 10.1126/science.aea1272

**Published:** 2026-02-12

**Authors:** Mohamed A. El-Brolosy, Atharv Oak, An T. Hoang, Yassine Damergi, André Fischer, Reuben A. Saunders, Jingchuan Luo, Amer Balabaki, Jeremy Guez, Troy W. Whitfield, Seth R. Goldman, Arash Latifkar, Yuancheng Ryan Lu, Didier Y.R. Stainier, Konrad J. Karczewski, Olivia Corradin, Jonathan S. Weissman

**Affiliations:** 1 Harvard Society of Fellows; Cambridge, MA, 02138, USA.; 2 Whitehead Institute for Biomedical Research; Cambridge, MA, 02142, USA.; 3 Program in Medical and Population Genetics, Broad Institute of MIT and Harvard, Cambridge, MA 02142, USA.; 4 Analytic and Translational Genetics Unit, Massachusetts General Hospital, Boston, MA 02114, USA.; 5 Department of Biology, Massachusetts Institute of Technology; Cambridge, MA 02142, USA.; 6 Nascent Transcriptomics Core, Department of Biological Chemistry and Molecular Pharmacology, Harvard Medical School, Boston, MA 02115, USA.; 7 Max Planck Institute for Heart and Lung Research; Bad Nauheim, 61231, Germany.; 8 Novo Nordisk Foundation Center for Genomic Mechanisms of Disease, Broad Institute of MIT and Harvard, Cambridge, MA 02142, USA.; 9 Howard Hughes Medical Institute, Massachusetts Institute of Technology; Cambridge, MA 02142, USA.; 10 David H. Koch Institute for Integrative Cancer Research, Massachusetts Institute of Technology; Cambridge, MA 02142, USA.

## Abstract

Transcriptional adaptation (TA) is a genetic robustness mechanism through which mutant mRNA decay induces sequence-dependent upregulation of so-called adapting genes. How cytoplasmically generated mRNA fragments impact nuclear transcription remains poorly understood. Using genome-wide CRISPR screens, we uncover ILF3 as an RNA-binding protein connecting cytoplasmic mRNA decay and transcription during TA, and show it is required for a range of TA substrates. ILF3 is enriched at adapting genes’ RNAs, and its artificial recruitment via dCas13 promotes gene expression. Using tiling oligonucleotide screens, we identify trigger RNA fragments that activate adapting genes when introduced into cells. Further functional dissection reveals critical role for homology between trigger and target sequences. These findings enhance our molecular understanding of TA and inform design of programmable oligonucleotides for gene expression augmentation.

Organisms must maintain developmental fidelity amidst genetic variability arising across billions of cell divisions. This evolutionary conundrum has necessitated the emergence of sophisticated adaptive mechanisms to counteract mutations. Among the most severe perturbations are loss-of-function protein-truncating variants (PTVs), such as nonsense mutations. Such disruptions, especially when affecting both alleles of a gene, may cause severe developmental consequences. However, an increasing number of animals engineered with PTVs in critical genes were reported to have no obvious phenotype (1). Furthermore, the identification of loss-of-function PTVs, including within disease-relevant genes, in phenotypically healthy individuals (2–8) underscore the importance of studying genetic robustness. Nonsense mutations create mRNAs with premature termination codons (PTC) that are often degraded by the nonsense-mediated decay (NMD) pathway (9). Additional cytoplasmic RNA surveillance mechanisms that degrade mutant transcripts include non-stop decay (NSD), which targets RNAs lacking a stop codon (10, 11), and no-go decay (NGD), which degrades transcripts where mutations create ribosome-stalling secondary structures (12). Transcriptional adaptation (TA) is a recently identified mode of genetic robustness through which mutant mRNA degradation can lead to upregulation of genes exhibiting sequence similarity with the mutated gene’s mRNA. Such genes, termed adapting genes, may include functionally related paralogs (13, 14), whose upregulation can provide functional compensation, thereby conferring genetic robustness. For example, nonsense mutations in the zebrafish extracellular matrix gene *egfl7* result in no obvious phenotype, while knockdown of the gene using translation blocking morpholinos lead to vascular defects. This discrepancy was shown to result from the upregulation of other extracellular matrix genes observed only in the mutant, but not knockdown animals (15). Unlike other robustness mechanisms, TA is triggered not by the loss of protein function but by mRNAs harboring destabilizing mutations and mRNA degradation, possibly through the decay intermediates or their derivatives (13, 14). Consequently, alleles failing to transcribe the mutated gene (e.g., full-locus deletion alleles) do not exhibit TA, and display more severe phenotypes. TA has been observed for several genes in different model organisms including *C. elegans (16, 17)*, silkworm (18), salamander (19), zebrafish, mouse (13, 14, 20, 21), and human (22, 23) (reviewed in (1, 24, 25)), highlighting its importance as a buffering mechanism for mutations. However, despite identifying mRNA decay as the trigger for TA, the underlying molecular mechanisms remain poorly explored. It remains unclear how mRNA decay intermediates (e.g., those generated by NMD or NSD) are translocated from the cytoplasm to the nucleus to modulate the expression levels of the adapting genes in a sequence-dependent manner (13, 14). Moreover, the nature of those decay intermediates inducing TA remain elusive. We hypothesized the existence of a shuttling RNA binding protein (RBP) connecting cytoplasmically-generated mRNA decay fragments to nuclear transcription. Therefore, we leveraged functional genomics and biochemical approaches to characterize underlying mechanisms.

## Results

### CRISPR screen identifies ILF3 as a mediator of TA

**Fig. 1.**
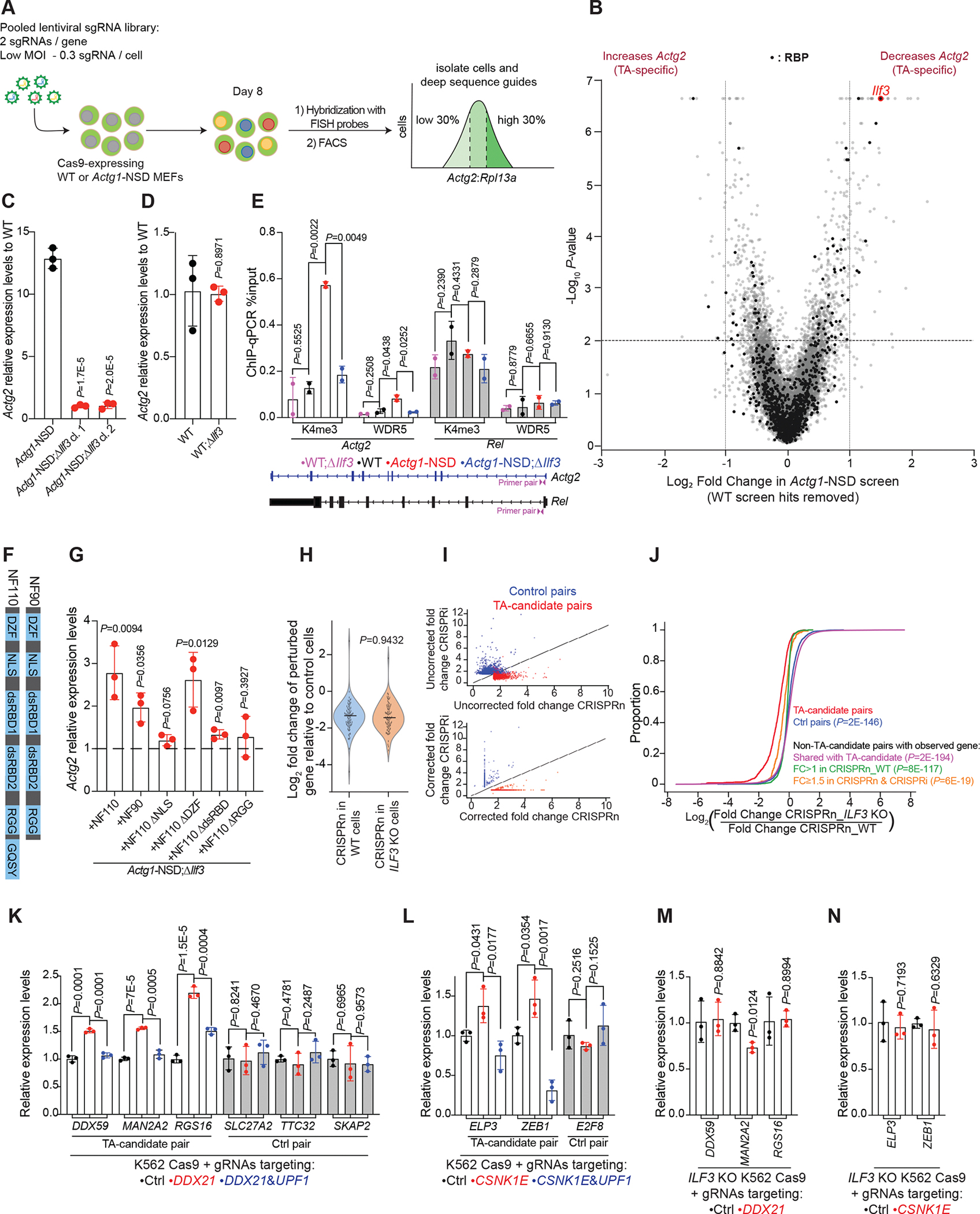
Genetic screens identify ILF3 as a mediator of transcriptional adaptation. (**A**) Schematic showing the Flow-FISH-based genome-wide CRISPR-Cas9 screens used in *Actg1*-NSD and WT cells. (**B**) Gene-level enrichment of the average scores of the sgRNAs in the bottom 30% of *Actg2*/*Rpl13a* expressing cells relative to the top 30% of expressing cells, plotted against MAGeCK-calculated *P*-values obtained from two independent replicates of a genome-wide CRISPR screen in *Actg1*-NSD cells. Genes scoring as hits in the counter screen in WT cells were removed prior to the MAGeCK analysis, and hence the figure looks different than fig. S1C. *Ilf3* is encircled in red. Black dots represent known RBPs (139, 140): RNA binding proteins that are not RNA decay factors. (**C**) Quantitative polymerase chain reaction (qPCR) analysis of *Actg2* mRNA expression levels in *Actg1*-NSD cells and the two generated *Actg1*-NSD;Δ*Ilf3* clones relative to WT cells. (**D**) Quantitative polymerase chain reaction (qPCR) analysis of *Actg2* mRNA expression levels in the generated WT;Δ*Ilf3* clone relative to WT MEFs. (**E**) ChIP-qPCR analysis of WDR5 and H3K4me3 occupancy near TSS of *Actg2*, or *Rel* (as a control locus), in WT, *Actg1*-NSD, *Actg1*-NSD;Δ*Ilf3* and WT;Δ*Ilf3* cells. The *Actg2* and *Rel* loci are shown below the plot, with the position of a primer pair at the TSS shown (purple triangles). (**F**) Cartoon showing the two different ILF3 isoforms, and known domains in the two proteins. DZF: Domain associated with Zinc Fingers; NLS: Nuclear Localization Signal; dsRBD: Double-Stranded RNA Binding Domain. RGG: single stranded RNA binding domain. (**G**) qPCR analysis of *Actg2* mRNA expression levels in *Actg1*-NSD;Δ*Ilf3* cells transduced with plasmids expressing FLAG-tagged full length NF90 or NF110 or NF110 with the indicated truncations under the control of *Ilf3* endogenous promoter relative to control (dashed line). (**H**) Fold change of expression levels of the genes targeted (perturbed-genes) in the CRISPRn Perturb-seq experiment performed on WT and *ILF3* KO K562 cells across each perturbation relative to non-targeting control gRNAs. n=84. (**I**) Top: Expression levels of the assessed genes within the identified TA-candidate (n = 754) and control pairs (n = 2559) upon CRISPRn or CRISPRi-mediated perturbation, relative to control cells. Bottom: Same as top, except that for genes whose expression change was not significant after multiple-testing correction (e.g., fold-change = 0.8 but the *P*-value was not significant), the fold change was set to 1 (no change). This “corrected fold-change” therefore reflects only statistically significant changes, while nonsignificant ones are shown as unchanged (FC = 1). Each dot represents a gene pair. (**J**) Cumulative distribution of the log_2_ of the ratio of fold change of the expression levels of the observed genes in the indicated gene pair groups relative to control upon perturbing a gene with CRISPRn in *ILF3* KO to that of WT K562s. Plotted gene pairs are TA-candidate pairs (red line, n=754), Ctrl Pairs (blue line, n=2559), non-TA-candidate pairs where the observed (assessed) genes were found in the TA-candidate list (magenta line, n=52166), all other non-TA-candidate gene pairs where the observed gene is significantly upregulated (fold-change >1, Padj≤0.05) in the CRISPRn Perturb-seq dataset from WT cells (green line, n=1644), and a further subset of that containing only gene pairs where the observed (assessed) gene was upregulated with a fold-change ≥1.5 and Padj≤0.05 both upon CRISPRn or CRISPRi perturbation in WT cells (orange line, n=299). FC: fold-change. These data show that observed genes in TA-candidate pairs exhibit the strongest downregulation upon ILF3 loss, relative to all other groups. The comparison to non–TA-candidate pairs containing observed genes also present in TA-candidate pairs (magenta line) highlights that the decrease in adapting (observed) gene expression in the TA-candidate group is specific to their respective perturbed partners, as these observed genes were less affected by other perturbations. Among TA-candidate pairs, 54.3% of adapting genes were downregulated by at least 1.5-fold, and 71.8% by at least 1.25-fold upon ILF3 loss. To enable log_2_ transformation, zero values were replaced with half the lowest non-zero value in each dataset. *P* values (Mann–Whitney U test) are reported relative to the TA-candidate group. (**K**) qPCR analysis of the indicated genes’ mRNA expression levels in K562-Cas9 cells that express a non-targeting (control), or *DDX21* sgRNA, or *DDX21* and *UPF1* gRNAs. (**L**) qPCR analysis of the indicated genes’ mRNA expression levels in K562-Cas9 cells that express a non-targeting (control), or *CSNK1E* sgRNA, or *CSNK1E* and *UPF1* gRNAs. (**M**) qPCR analysis of the indicated genes’ mRNA expression levels in Cas12-generated-*ILF3* polyclonal KO K562-Cas9 cells transduced with either a non-targeting (control), or *DDX21* sgRNA. (**N**) qPCR analysis of the indicated genes’ mRNA expression levels in Cas12-generated-*ILF3* polyclonal KO K562-Cas9 cells transduced with either a non-targeting (control), or *CSNK1E* sgRNA. (**C, D, G, K-N**) n = 3 biologically independent samples. Wild-type or control expression levels were set at 1 for each assay. Data are mean ± s.d., and a two-tailed Student’s t-test was used to calculate *P*-values. (**H, J**) Mann-Whitney U test was used to calculate *P*-value.

We took a genetic approach to identify factors mediating TA. We first focused on *Actg1,* a well characterized TA model (13, 26). In mouse embryonic fibroblasts (MEFs), mRNA-destabilizing mutations in *Actg1* (as the NSD allele (13), hereafter referred to as *Actg1*-NSD) result in upregulation of the paralog *Actg2*. In contrast, full-locus deletions of *Actg1* either do not trigger this response or do so to a much lesser extent (13, 26). We performed a genome-wide CRISPR-Cas9 knockout screen to identify factors required for the mutant *Acgt1*-NSD mRNA to induce *Actg2* upregulation using a flow-FISH (fluorescence in-situ hybridization) strategy that allowed direct assessment of endogenous *Actg2* mRNA levels (27) (Fig. 1A, fig. S1A). For those experiments, we stably integrated a transgenic wild-type (WT) copy of *Actg1* into the mutant *Actg1*-NSD cells, allowing us to monitor TA-induced *Actg2* upregulation independently of ACTG1 protein function loss. To avoid factors that influence *Actg2* expression in a TA-independent manner, we also performed a counter screen in WT cells (fig. S1B). Consistent with their reported roles in TA (13, 14), we observed that mRNA decay factors and components of the COMPASS complex, which deposits the permissive H3K4me3 histone mark, modulate *Actg2* expression (fig. S1C). Notably, the screen identified ILF3 as the RBP with the strongest impact on *Actg2* expression levels, specifically in mutant *Actg1*-NSD, but not WT, cells (Fig. 1B).

ILF3 is a ubiquitously expressed RBP that shuttles between the cytoplasm and the nucleus (28) and binds to small RNAs (29) and mRNAs, without apparent specificity to untranslated regions (UTRs) versus coding sequences (28, 30, 31). It interacts with the ribosome and the NMD machinery (32), and is found in p-bodies (33). ILF3 can also contribute to activating gene expression (34–37), and two of its known interactors involved in gene expression, YY1 and PRMT1 (38–40), were also identified as hits in the screen (fig. S1C). Thus, ILF3 fits the expected criteria for an RBP that can mediate TA. To validate the screen results, we generated two homozygous *Ilf3* knockout (KO) clones in *Actg1*-NSD MEFs (hereafter referred to as *Actg1*-NSD*;ΔIlf3*), and a KO clone in WT cells (hereafter referred to as WT;Δ*Ilf3*) (fig. S1D, E). Loss of ILF3 decreased *Actg2* expression levels in the *Actg1*-NSD mutant, but not WT, cells (Fig. 1C, D). Notably, loss of ILF3 did not stabilize the mutant *Actg1*-NSD mRNA, whose decay was previously reported (13), suggesting that ILF3 functions downstream of mRNA decay in inducing TA (fig. S1F–H). Co-immunoprecipitation (IP) experiments, showed that ILF3 interacts with the NMD factor UPF1, as previously reported (32), and this interaction was diminished upon RNase treatment (fig. S1I). ILF3 also interacts with the active, phosphorylated form of UPF1 (9), with the interaction appearing to be diminished following RNase treatment (fig. S1I). However, given the low IP signal for p-UPF1, it remains possible that a partially RNase-resistant interaction exists but falls below detection limits. ILF3 also associates with the NSD factor PELO (41), which was partially decreased upon RNase treatment (fig. S1I). Additionally, ILF3 interacts with the COMPASS complex component WDR5, which contributes to H3K4me3 deposition, and this interaction was RNase-resistant (fig. S1I). Notably, loss of ILF3 appeared to lead to a reduction of both WDR5 and H3K4me3 at the *Actg2* transcription start site (TSS) in the *Actg1*-NSD mutant, but not WT, cells (Fig. 1E).

ILF3 has two major isoforms: NF90, and a C-terminally extended isoform, NF110 (Fig. 1F). Through rescue experiments in *Actg1*-NSD;Δ*Ilf3* cells, both isoforms increased *Actg2* expression, with NF110 showing a somewhat greater effect (Fig. 1G, fig. S1J, K). This result is consistent with previous observations that NF110 is a stronger activator of gene expression (34, 42). We observed that the nuclear localization signal of ILF3 was required for the ability of NF110 to efficiently increase *Actg2* expression levels (Fig. 1G). Furthermore, deletion of the DZF domain, required for dimerization with other DZF-containing proteins as ILF2, ZFR and STRBP (43), did not seem to impair NF110’s ability to increase *Actg2* expression levels in *Actg1*-NSD;Δ*Ilf3* cells (Fig. 1G), and loss of ILF2 in *Actg1*-NSD cells did not decrease *Actg2* levels (fig. S1C, L, M). ILF3 has two double-stranded RNA-binding domains (dsRBDs), and a single-stranded RNA-binding domain: the RGG domain (44). Deletion of the two dsRBDs or the RGG domain largely diminished the ability of NF110 to increase *Actg2* expression levels (Fig. 1G). A study reported that the dsRBDs and RGG domains act cooperatively to flexibly engage different RNA targets, which may explain why both domains are required for increased *Actg2* expression levels (44). Collectively, these findings provide evidence that RNA binding is essential for ILF3’s role in TA. Future studies may elucidate specific residues or domains that enable ILF3 to mediate TA distinctly from its other cellular roles (34–37, 45–47).

### Evidence for a broader role of ILF3 in TA

Next, we sought to determine whether ILF3 is required more generally for TA. To identify a broader set of potential TA responses, we used two complementary Perturb-seq strategies — CRISPR screens coupled to single-cell RNA-sequencing (48–50) — in K562 cells. We reasoned that perturbing genes using the nuclease-active CRISPR/Cas9 system (CRISPRn) would introduce frameshift mutations resulting in NMD-inducing PTCs, which could trigger TA. By contrast, CRISPR-interference (CRISPRi) would repress transcription without inducing mRNA decay (51), and thereby fail to induce TA. However, it provides a useful control for transcriptional changes resulting from TA-independent protein loss-of-function effects. Comparing transcriptional responses between both methods of perturbation could thereby enable systematic interrogation of TA responses. A comprehensive dataset from a genome-wide CRISPRi Perturb-seq experiment in K562 was previously generated by our group (52), so we performed a complementary CRISPRn Perturb-seq experiment targeting 147 genes (selected to represent various gene categories, Methods) —for which 84 genes passed quality control (Methods). We observed a decrease in the mRNA levels for most of the 84 targeted genes upon CRISPRn perturbation, suggestive of NMD (Fig. 1H), and confirming the efficacy of our CRISPRn libraries. Gene pairs in which the assessed gene (hereafter referred to as the observed gene) was upregulated more strongly upon CRISPRn-mediated perturbation of a given gene (the perturbed gene), than with CRISPRi, represented candidate transcriptional adaptation responses (TA-candidate pairs; determined using a cutoff of ≥1.5-fold change upon CRISPRn perturbation and CRISPRn/CRISPRi fold change ratio ≥1.5, Fig. 1I, Methods). In these cases, the upregulated observed genes represented potential adapting genes. As a control group, we used gene pairs in which the observed gene was upregulated more strongly in response to CRISPRi-mediated perturbation (hereafter referred to as control gene pairs; Fig. 1I, Methods; Supplementary Text). In total, we identified 754 TA-candidate gene pairs and 2559 control pairs. We performed several quality control analyses demonstrating that the identification of TA-candidate pairs was not driven by several tested perturbation-specific confounders and that these pairs exhibit properties consistent with previously characterized TA models, including sequence similarity and functional coherence between perturbed and adapting genes (Supplementary Text, fig. S2, S3). Thus, this set provides a pool of TA-candidate pairs which can be used to globally explore requirements for TA, but, as with any large-scale study, individual gene pairs need to be validated before undertaking focused functional investigations.

The set of TA-candidate gene pairs allowed us to investigate if ILF3 is required more generally for TA. To this end, we repeated the CRISPRn Perturb-seq experiment in *ILF3* KO cells (fig. S4A–C, Supplementary Text) at day 8 post-transduction with a Cas12-based *ILF3* KO vector, a time point chosen to precede major proliferation defects observed with prolonged ILF3 loss in K562s, but not detected in MEFs (fig. S4D). We found that most adapting genes in TA-candidate pairs were downregulated upon loss of ILF3 (Fig. 1J), suggesting that ILF3 may act as a broad regulator of TA. Notably, adapting (observed) genes within TA-candidate gene pairs appeared to be more markedly downregulated relative to multiple control groups (Fig. 1J). This pattern suggests that adapting genes within TA-candidate gene pairs are disproportionately affected by ILF3 loss, consistent with a role for ILF3 in mediating TA. Similar to the *Actg1* model, loss of ILF3 did not lead to increased levels of the mutant mRNAs of the perturbed genes (Fig. 1H), suggesting that ILF3 likely acts downstream of mRNA decay in TA. For gene pairs exhibiting sequence similarity, we compared the alignment region from the perturbed gene (which represent potential TA-inducing mRNA decay intermediates) with ILF3 motifs identified from published eCLIP-seq data in WT K562 cells (53, 54). Alignment regions from TA-candidate pairs displayed higher sequence similarity to ILF3 binding motifs relative to control pairs, and a higher percentage of them had a high-confidence match to the motifs (fig. S4E, F, Methods). Furthermore, TA-candidate pairs where the alignment region had a high-confidence match to an ILF3 motif appeared to display higher upregulation levels of the respective adapting genes relative to those without (fig. S4G). It is, nevertheless, important to recognize that RBP motifs tend to be short and degenerate, and that structural features that may dictate recognition of an RNA were not captured in this analysis, nor were other potentially unidentified motifs. Taken together, the data provide further evidence that ILF3 is a regulator of TA, and suggest that ILF3 binding preferences could influence the recognition of TA-inducing mRNA decay intermediates.

We selected two genes with differing numbers of adapting genes for validation prior to more detailed mechanistic investigation: *DDX21*, which had 21 TA-candidate adapting genes, and *CSNK1E*, which had only two. Upon CRISPRn-mediated perturbation of *DDX21*, we tested three top-ranked adapting genes and confirmed their upregulation, including one of its paralogs *DDX59*. Similarly, both adapting genes for *CSNK1E* were validated (Fig. 1K, L). Notably, three randomly selected control genes for *DDX21* and the single control gene for *CSNK1E* were not upregulated (Fig. 1K, L), in line with the Perturb-seq results. Genetic inactivation of *UPF1*, a key component of NMD shown to be required for several TA models (13), abolished the upregulation of the respective adapting genes in both models (Fig. 1K, L, fig. S5A). Additionally, loss of ILF3 in the *DDX21* and *CSNK1E* models similarly abrogated the upregulation of the corresponding adapting genes (Fig. 1M, N, fig. S5B). These effects appeared specific: the adapting genes’ expression remained largely unchanged in *UPF1* and *ILF3* knockout cells when the corresponding perturbed gene was not targeted (fig. S5C, D). ILF3 loss did not lead to increased levels of the mutant mRNA, in contrast to UPF1 loss, which did (fig. S5E, F). Consistently, half-lives of the mutant mRNAs were comparable in wild-type and *ILF3* knockout cells (fig. S5G, H), suggesting that ILF3 does not influence mRNA stability and likely acts downstream of decay. Furthermore, ILF3-independent proliferation-limiting conditions did not prevent TA in the *DDX21* model (Supplementary Text, fig. S6).

### ILF3 is enriched at adapting genes’ RNAs, promoting their transcription

**Fig. 2.**
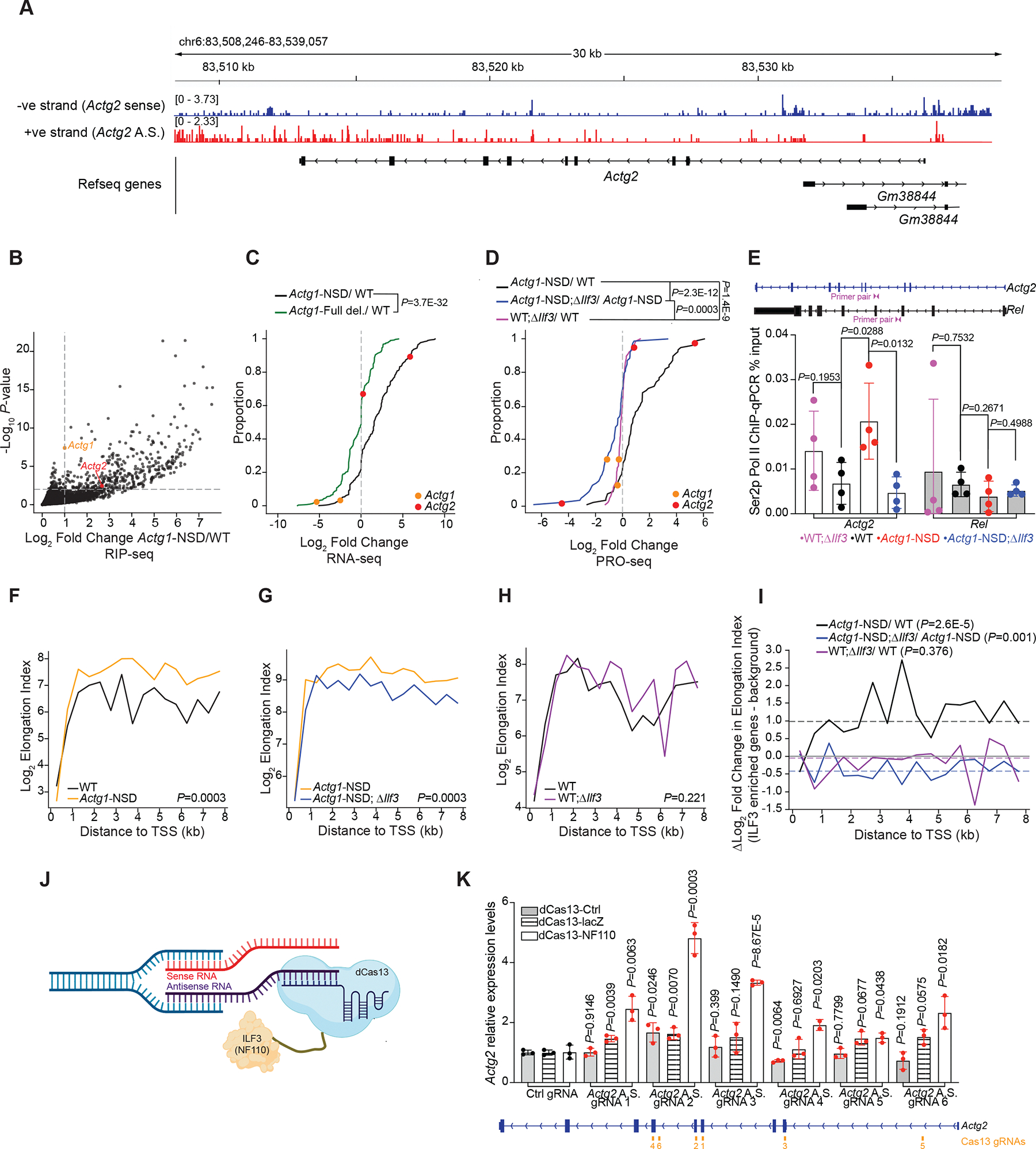
ILF3 is enriched at adapting genes’ RNAs from adapting genes’ loci, and its artificial recruitment is sufficient to promote gene expression. (**A**) IGV (141) tracks of the *Actg2* locus showing PRO-seq signals in WT cells from the positive and negative strands. A.S.: antisense. (**B**) log_2_ (fold-change) of RNA IP/input signal in ILF3 nuclear RIP-seq experiments in *Actg1*-NSD cells relative to that in WT MEFs plotted against -log_10_ (*p*-value) as calculated by DESeq2 (121), showing RNAs where log_2_ fold-change was > 0. *Actg1* and *Actg2* are both highlighted. 383 RNAs were identified to be more associated with ILF3 in *Actg1*-NSD cells relative to WT (identified as log_2_(fold-change) ≥1 and *P*-val ≤0.01). The results presented here were obtained using an unstranded analysis of the sequencing data because the RIP-seq was performed under crosslinking conditions that can enrich for both sense and antisense transcripts if they are spatially proximal. Stranded analyses are presented in fig. S7D. (**C**, **D**) Cumulative distribution of the log_2_ fold change of gene expression between the indicated cell lines from RNA-seq (**C**) or PRO-seq (**D**) analysis for RNAs identified from RIP-seq analysis to be more associated with ILF3 in *Actg1*-NSD cells relative to WT (identified as as log_2_(fold-change) ≥1 and *P*-val ≤0.01). *Actg1* and *Actg2* are both highlighted. RNA-seq of *Actg1*-NSD/WT and *Actg1*-Full del./WT was previously published (13), while the PRO-seq data were generated in this study. The smaller number of genes shown in the PRO-seq plots (n=78) compared to the RNA-seq plots (n=383) reflects the refinement of gene annotations (GGA) workflow (Methods), which includes in the PRO-seq analysis only those genes with high-confidence dominant transcription start and end site assignments. (**E**) ChIP-qPCR analysis of phosphorylated-Ser2-RNA polymerase-II occupancy at the *Actg2,* or *Rel* (as a control locus), gene body in WT, *Actg1*-NSD, *Actg1*-NSD;Δ*Ilf3* and WT;Δ*Ilf3* cells. *Rel* is used as a control locus. (**F-H**) The log_2_-transformed median of elongation index across the first 15 consecutive 500-nt bins following the transcription start site (TSS) bin for ILF3-enriched genes identified by RIP-seq that exhibited significantly increased transcription (*P-val* ≤ 0.05) in the *Actg1*-NSD cells relative to WT based on PRO-seq data (n = 46). Three different panels are shown as PRO-seq experiments were collected in three batches (see Methods). A Mann Whitney U-test was used for statistical inference. (**I**) Difference in log_2_ fold change in elongation index between ILF3-enriched genes that exhibited significantly increased transcription (P-val ≤ 0.05) in the *Actg1*-NSD cells relative to WT based on PRO-seq data, and other upregulated genes not enriched for ILF3 binding (used as background). An analysis performed to confirm that elongation changes for the ILF3-enriched genes (Fig. 2 F–H) are not explained by background trends. Elongation index was computed across 15 consecutive 500-nt bins downstream of the TSS bin. Each line represents a pairwise genotype comparison, with values at each bin reflecting log_2_ fold change in median elongation index for ILF3-enriched genes minus log_2_ fold change in median elongation index for background non-ILF3-enriched genes. Dashed lines indicate the median Δlog_2_ value across all bins for each comparison. For each genotype comparison, a Mann–Whitney U test was used for statistical inference on whether elongation index fold changes at ILF3-enriched genes differ significantly from those of background genes across the tested bins (see Methods for alternative statistical inference procedure). (**J**) Cartoon illustrating the engineered dCas13-NF110 protein targeting antisense transcripts. (**K**) qPCR analysis of *Actg2* mRNA expression levels upon transducing cells expressing dCas13-NF110 fusion protein (white bars) or dCas13 (grey bars) or dCas13-lacZ (bars with stripes) with a non-targeting (control) gRNA, or gRNAs targeting antisense of *Actg2*. A.S.: antisense. n = 3 biologically independent samples. Control expression levels were set at 1 for each assay. Data are mean ± s.d., and a two-tailed Student’s t-test was used to calculate *P* values.

Next, we hypothesized that ILF3 might be guided by mRNA decay intermediates to the adapting genes’ locus in the nucleus. In theory, those decay intermediates could exhibit complementarity, and bind, to either DNA or RNA at the adapting genes’ locus. In the case of paralogous adapting genes, this complementarity would be expected to occur either with the template strand of DNA or with natural antisense RNAs transcribed at those loci (55). Overexpression of RNASEH1, which disrupts RNA:DNA hybrids (56), in *Actg1*-NSD cells did not reduce *Actg2* upregulation (fig. S7A, B). Similarly, its overexpression in K562 cells did not reduce the upregulation of the adapting genes upon CRISPRn-mediated perturbation of *DDX21* or *CSNK1E* —and in some cases, even enhanced it (fig. S7A, B). These findings argued against RNA:DNA hybrids as the trigger for TA. Notably, precision nuclear run-on sequencing (PRO-seq) revealed antisense transcription at the *Actg2* locus (hereafter referred to as *Actg2* antisense RNAs) (Fig. 2A). As reported previously (55), we also observed that antisense transcription was common in both MEFs and K562 cells (fig. S7C). We reasoned that cross-linked ILF3 RNA immunoprecipitation followed by sequencing (RIP-seq) on nuclear fractions of WT and *Actg1*-NSD MEFs will allow the identification of RNAs (including antisense RNAs) at which ILF3 is preferentially enriched in *Actg1*-NSD cells. Cross-linking would allow capturing target RNAs that associate indirectly with ILF3 through hybridization to potential decay intermediates. Indeed, using an unstranded analysis, ILF3 was found to be more enriched with RNAs originating from the *Actg2* locus in the *Actg1*-NSD cells relative to WT (Fig. 2B). Furthermore, we observed that ILF3 was more strongly enriched with RNAs originating from other gene loci (hereafter referred to as ILF3-enriched genes), including from *Actg1*, in the *Actg1*-NSD cells (Fig. 2B). Notably, most of those genes remain enriched when performing strand-specific analysis using only sense or antisense reads (fig. S7D), which is expected given that cross-linking will enrich for RNAs from both strands when they are in close proximity. Those ILF3-enriched genes exhibited higher levels of sequence similarity to *Actg1* mRNA relative to genes with unchanged ILF3 recruitment between *Actg1*-NSD and WT cells (Fig. S7E). In addition, gene ontology analysis suggested that many of these genes are involved in biological processes related to ACTG1 function, and thus could potentially contribute to functional compensation (fig. S7F). Notably, those genes showed a strong propensity to be upregulated in *Actg1*-NSD cells relative to WT, and not in *Actg1* full locus deletion cells where TA does not occur (13) (Fig. 2C), suggesting that they are TA targets. PRO-seq data argued that the majority of the increase in mRNA levels identified by RNA-seq was due to increased transcription (Fig. 2D). Furthermore, knockout of *Ilf3* led to decreased transcription of the majority of those genes in *Actg1*-NSD but not WT cells (Fig. 2D). Notably, CRISPRn-mediated perturbation of *DDX21* in K562 cells also led to increased ILF3 association with RNAs originating from both the mutated *DDX21* locus itself and its paralog *DDX59* (fig. S7G).

The screen showed that loss of factors promoting Pol II pausing (e.g., the NELF complex (57)) enhances TA, while loss of factors promoting transcription elongation decreased TA (fig. S7H). We therefore asked whether the increased transcription observed for the majority of the ILF3-enriched genes (Fig. 2C) might result from enhanced transcriptional elongation. Chromatin immunoprecipitation revealed increased Ser2-phosphorylated RNA polymerase-II—indicative of elongating Pol II—at the *Actg2* gene body in *Actg1*-NSD cells relative to WT, in an ILF3-dependent manner (Fig. 2E). To test this hypothesis more broadly, we performed Transient Transcriptome (TT)-seq, which measures nascent RNA synthesis (58), and calculated the elongation index (59–61) by dividing TT-seq signal by PRO-seq signal, which reflects RNA Pol II occupancy. Indeed, those genes exhibited higher elongation indices in *Actg1*-NSD cells relative to WT (Fig. 2F), and *Ilf3* knockout reduced their elongation index in *Actg1*-NSD but not WT cells (Fig. 2G, H). These differences were not due to global changes in elongation index between conditions, as both the increased elongation observed in *Actg1*-NSD versus WT and the decreased elongation upon *Ilf3* knockout were preserved after normalization to elongation measurements from all other genes that were similarly significantly upregulated by PRO-seq (*P*≤0.05) in *Actg1*-NSD relative to WT but not enriched for ILF3 binding (Fig. 2I). Together, these data are consistent with a previously reported model of ILF3-dependent transcriptional activation through enhanced elongation (35). Furthermore, the CRISPR screen uncovered previously unknown epigenetic modulators of TA. In addition to the previously-identified COMPASS complex (13, 14), which has also been shown to promote transcriptional elongation (62), the CRISPR screen identified the ILF3 interactors and transcriptional activators PRMT1 and YY1 (38–40) (fig. S1C), and BRG1 (encoded by *Smarca4*), the catalytic subunit of the SWI/SNF complex that increases chromatin accessibility (63) (fig. S8A). Of note, PRMT1 was reported to recruit BRG1 (64). Chromatin-immunopreciptation (ChIP) experiments revealed an apparent ILF3-dependent enrichment of BRG1, PRMT1 and YY1 at *Actg2* TSS in *Actg1*-NSD cells (fig. S8B). The identification of the SWI/SNF complex explains the previously observed increased chromatin opening at the adapting gene’s locus (13, 26). Altogether, these data suggest that ILF3 is enriched at RNAs at the adapting gene’s locus, promoting gene expression through recruitment of epigenetic modifiers and enhancing transcription elongation.

### Artificial recruitment of ILF3 is sufficient to promote gene expression

We next reasoned that if mRNA decay intermediates direct ILF3 to *Actg2* antisense RNAs, then its artificial recruitment should be sufficient to increase gene expression. To this end, we engineered a fusion protein of dead Cas13 (dCas13, an RNA-targeting Cas protein (65)) with two nuclear localization signals, and NF110 (the ILF3 isoform which best activates TA (Fig. 1G, (34, 42)); hereafter referred to as dCas13-NF110) (Fig. 2J, fig. S9A). Using gRNAs targeting *Actg2* antisense RNAs to recruit dCas13-NF110 led to *Actg2* mRNA upregulation in MEFs (Fig. 2K). In contrast, the same gRNAs did not lead to *Actg2* upregulation in most cases when used with dCas13 alone (expressed at much higher levels than dCas13-NF110) or with dCas13-lacZ (expressed at comparable levels as dCas13-NF110); in rare cases, weak effects were observed but remained below those seen with dCas13-NF110 (Fig. 2K, fig. S9A). Notably, recruitment of dCas13-NF110, but not the control dCas13-lacZ, also appeared to lead to increased WDR5, H3K4me3, BRG1, PRMT1 and YY1 at *Actg2* TSS (fig. S9B, C). gRNAs targeting antisense transcripts of *Cdk9* and *Rel*, two previously identified TA models (13), similarly led to upregulation of the respective adapting genes (fig. S9D). Notably, recruiting dCas13-NF110 to the 5’ end of *Actg2* sense pre-mRNA also led to *Actg2* mRNA upregulation (fig. S9E). Similarly, targeting dCas13-NF110 to the pre-mRNAs of *Serpine1* and *Nrg1*, also led to upregulation of those genes (fig. S9F). These results suggest that ILF3 by itself is sufficient to increase gene expression when recruited to RNAs at the adapting genes’ locus.

### Trigger screens identify sequences of the mutant mRNA responsible for TA

**Fig. 3.**
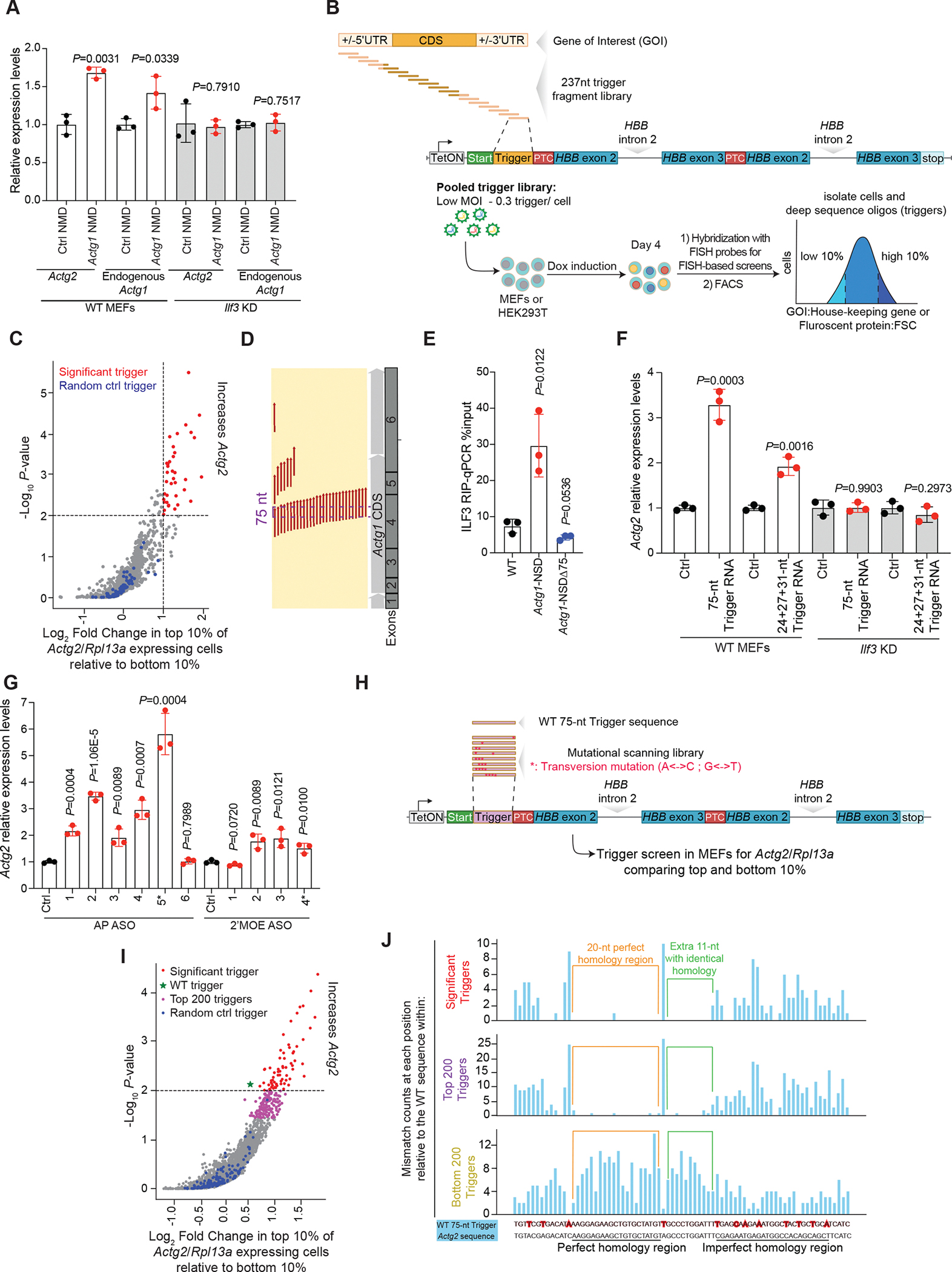
Tiling trigger screen identifies regions of mRNA sufficient for TA-mediated paralogous gene upregulation. (**A**) qPCR analysis of *Actg2* and the endogenous *Actg1* mRNA expression levels in WT (white bars) or *Ilf3* knockdown (kd) (grey bars) MEFs expressing *Actg1* coding sequence, or a control (GFP-2A-RFP) message from the NMD vector. When the stable *Actg1* NMD cells were maintained in culture for several weeks and the experiment was repeated using Tet system–approved FBS prior to doxycycline induction, we observed a stronger 3- to 4-fold upregulation of *Actg2*. (**B**) Schematic showing the Flow-FISH-based *Actg1* trigger screen. (**C**) Enrichment of triggers in the top 10% of *Actg2*/*Rpl13a* expressing cells relative to the bottom 10% of expressing cells, plotted against one-sided MAGeCK-calculated *P* values obtained from two independent replicates of the trigger screen. Red dots represent triggers that significantly increased *Actg2* expression, while blue dots represent the control random scrambled triggers showing that none of them scored as significant. (**D**) Location of the significant triggers identified from the trigger screen mapped onto *Actg1* mRNA sequence. Most of the identified trigger sequences shared the 75-nucleotide region (TGTTCGTGACATAAAGGAGAAGCTGTGCTATGTTGCCCTGGATTTTGAGCAAGAAATGGCTACTGCTGCATCATC). (**E**) Cross-linked nuclear RIP-qPCR analysis of ILF3 enrichment with RNAs from *Actg2* in *Actg1*-NSD and *Actg1*-NSDΔ75 (*Actg1*-NSD MEFs where the 75-nt trigger sequence region within exon 4 of *Actg1* was deleted) MEFs relative to WT. (**F**) qPCR analysis of *Actg2* mRNA expression levels in WT (white bars) or *Ilf3* knockdown (grey bars) MEFs transfected with the indicated trigger RNAs. The 24, 27, 31 nt RNAs were selected as they individually induced a significant mild upregulation of *Actg2* when transfected to cells (fig. S9I). The control used for the 24+27+31 experiment in WT MEFs the same as that used for 21–31 nt trigger RNA experiments the in fig. S10I. (**G**) qPCR analysis of *Actg2* mRNA expression levels in WT MEFs transfected with the indicated antisense oligos (ASOs) targeting antisense RNAs in the *Actg2* locus homologous to the 75-nucleotide identified from the trigger screen relative to non-targeting control. * Indicates ASOs that target the perfect 20 nt homology region. AP: Affinity Plus (lock nucleic acid ASOs). 2’MOE: 2’-Methoxyethyl ASOs. (**H**) Schematic illustrating the mutagenesis screen of the identified 75-nt trigger sequence. The library includes all possible single, double, adjacent triple, and adjacent quadruple transversion mutations (A<->C and G<->T). (**I**) Enrichment of triggers in the top 10% of *Actg2*/*Rpl13a* expressing cells relative to the bottom 10% of expressing cells, plotted against one-sided MAGeCK-calculated *P* values obtained from two independent replicates of the mutagenesis screen. Red dots represent triggers that significantly increased *Actg2* expression, the green star represents the WT 75-nt trigger, the magenta dots represent the top 200 triggers per MAGeCK analysis in activating *Actg2*, and blue dots represent the control random scrambled triggers showing that none of them scored as significant. (**J**) Number of mismatches observed at each position of the 75-nt trigger RNA within the significant (top) or top 200 (middle) or bottom 200 (bottom) trigger sequences. Below, the *Actg1* 75-nt trigger RNA is shown, with red strokes indicating mismatches relative to the *Actg2* sequence displayed underneath. A region of perfect homology—a 20-nt stretch identical between *Actg1* and *Actg2*—is marked by orange lines in the plots. This is followed by an 11-nt region of additional identical sequence homology (marked in green), separated from the first by a single mismatch. The 3′ end of the RNA, contains multiple mismatches and is referred to as the imperfect homology region. Within both the significant and top 200 trigger sets, mutations were depleted in the perfect homology region and the adjacent 11-nt region, relative to other regions of the RNA, including the imperfect homology region. (**B, H**) Figure was created with BioRender.com. (**D**) Figure made in Snapgene. (**A, E, F, G**) n = 3 biologically independent samples. Control expression levels were set at 1 for each assay. Data are mean ± s.d., and a two-tailed Student’s t-test was used to calculate *P* values.

To determine what potentially guides ILF3 to RNAs at adapting genes’ loci, we sought to identify the mRNA fragments responsible for triggering TA. Transgenes designed to undergo NMD can induce TA in the absence of a mutation within the endogenous gene (14, 23, 66, 67). Using a similar transgenic approach (fig. S10A, B), we found that cloning *Actg1* into a vector designed to undergo NMD (hereafter referred to as NMD vector) leads to the upregulation of both *Actg2* and endogenous *Actg1* in wild-type MEFs, in a manner dependent on NMD and ILF3 (Fig. 3A. fig. S10C, D). To identify the critical sequences of *Actg1* required to upregulate *Actg2*, we cloned a library of 237-nt oligos tiling *Actg1* mRNA with 1-nt increments into the NMD vector and used them to screen, in a pooled fashion, for sequences that can induce *Actg2* upregulation using the flow FISH strategy (Fig. 3B, hereafter referred to as trigger screen). Oligos that scored as hits that increase *Actg2* expression clustered to a defined 75-nucleotide window in exon 4 of *Actg1* (Fig. 3C, D, hereafter referred to as trigger sequence). This sequence displayed extensive homology with exon 7 of *Actg2* (fig. S10E). Notably, this 75-nucleotide region was sufficient to induce *Actg2* upregulation when cloned into the NMD vector (fig. S10F). Deletion of the 75-nucleotide region from the *Actg1* transgene in the NMD vector abolished *Actg2* upregulation (fig. S10F). Similarly, deletion of the same region from the endogenous *Actg1* locus in *Actg1*-NSD cells (hereafter referred to as *Actg1*-NSDΔ75 cells) led to a strong reduction of *Actg2*, but not *Actg1*, transcription levels (fig. S10G). The decreased *Actg1* mRNA levels is likely due to the extra deletion destabilizing the *Actg1*-NSDΔ75 mRNA. Notably, cross-linked ILF3 nuclear RIP-qPCR experiments showed that ILF3 association with RNAs from the *Actg2* locus in *Actg1*-NSDΔ75 cells was no longer enriched relative to WT cells (Fig. 3E), suggesting that the identified 75-nt trigger sequence plays a key role in guiding ILF3 to RNAs from the *Actg2* locus. Accordingly, recruiting dCas13-NF110 to antisense RNAs within the trigger-homologous region of *Actg2* led to strong upregulation of *Actg2* (fig. S10H). Transfection of cells with an RNA corresponding to the 75-nt trigger sequence (hereafter referred to as trigger RNA) led to *Actg2* upregulation in an ILF3-dependent manner (Fig. 3F). To identify if there are other smaller trigger RNA fragments that may originate from the 75-nt trigger sequence, we performed native ILF3 nuclear RIP-seq on *Actg1*-NSD cells followed by small RNA-sequencing. We detected small RNA fragments mapping to *Actg1*’s 75-nucleotide trigger sequence that may represent endogenous ILF3-associated RNAs. Upon transfection of nine of the identified RNAs into WT MEFs, we observed that three of them led to mild upregulation of *Actg2* (fig. S10E, I). Notably, co-transfection of the 3 RNAs together led to a stronger upregulation of *Actg2* in an ILF3-dependent manner (Fig. 3F). The extensive homology between those RNAs and *Actg2* (fig. S10E) suggested that they act through hybridizing to antisense RNA in the *Actg2* locus.

Targeting dCas13 alone, or dCas13-lacZ, to *Actg2* antisense RNAs led in some cases to upregulation of *Actg2*, albeit with milder levels compared to dCas13-NF110 (Fig. 2K, fig. S10H). Antisense RNAs can lower sense RNA expression through multiple mechanisms including 1) recruitment of chromatin-modifying complexes that deposit repressive histone marks or promote promoter methylation, 2) transcriptional interference via RNA polymerase collisions leading to premature transcription termination and 3) base-pairing with sense pre-mRNAs that can alter splicing, transcription, or affect RNA stability (68–90). The effects of the control dCas13 experiments could reflect direct interference of the gRNA–dCas13 complex with antisense RNA structure, accessibility, or expression levels, thereby potentially decreasing its capacity to recruit repressive chromatin modifiers and/or influencing its ability to hybridize with the sense pre-mRNA (those effects are hereafter referred to collectively as “interference with antisense RNAs”). While the exact mechanism underlying the interference in this context remains uncertain, these data suggested that interfering with antisense RNAs, thereby alleviating their potential negative regulatory effects on gene expression (68–90), could by itself also be contributing to TA, alongside ILF3-mediated recruitment of positive epigenetic modifiers. We thus reasoned that if such interference mechanisms can also contribute to TA, then direct depletion of these RNAs—as a possible way of achieving such interference—could elicit *Actg2* upregulation in a manner that can occur independently of ILF3. To test this hypothesis, we transfected gapmer antisense oligonucleotides (ASOs) targeting *Actg2* antisense RNAs within the region homologous to the trigger sequence. Several ASOs led to *Actg2* upregulation, with the more active locked nucleic acid–based Affinity Plus (AP) ASOs leading to a stronger upregulation than the less efficient 2′-O-methoxyethyl (2′MOE) ASOs (Fig. 3G, fig. S10J). Notably, these ASOs appeared to function in an ILF3-independent but RNASEH1-dependent manner (fig. S10K–M).

### Actg2 trigger activity depends on homology with Actg1

Next, we sought to determine the specific sequence requirements for activity of the 75-nucleotide trigger sequence. We designed a scanning mutagenesis library covering all single, double, and adjacent triple and quadruple transversion mutations (A↔C and G↔T). The library was cloned into the NMD vector and screened for variants based on their ability to activate *Actg2* (hereafter referred to as mutagenesis screen, Fig. 3H). The wild-type sequence shares extensive homology with exon 7 of *Actg2*, including a 20-nt perfect match in the 5’ half (perfect homology region), and an adjacent 11-nt stretch of identical homology, while the 3′ portion contained more mismatches (imperfect homology region) (fig. S10E). Sequences identified as significant in the screen, as well as the top 200 ranked by MaGeCK score (91)—based on their ability to activate *Actg2*—were depleted of mutations in the perfect homology region and the adjacent 11-nt stretch of identical homology, relative to regions with lower homology (Fig. 3I, J). Conversely, the bottom 200 sequences were enriched for mutations in these same regions, supporting their functional importance (Fig. 3J). These findings indicate that mutations within high-homology stretches are less tolerated, consistent with the idea that these regions are required for hybridization to antisense RNAs at the *Actg2* locus—a key step in driving TA. To validate these results, we designed new variants of the 75-nt trigger RNA containing three or four non-adjacent mutations within either the perfect or imperfect homology regions, using sequences not present in the mutagenesis screen. For the imperfect homology region, mutations targeted nucleotides that were identical between *Actg1* and *Actg2*. Upon transfection, mutations in the perfect homology region abolished *Actg2* upregulation, while mutations in the imperfect region retained activity, albeit at reduced levels compared to the WT sequence (fig. S10N). These results validate the important role of the perfect homology region and suggest that identical nucleotides in the imperfect region also contribute to full trigger RNA function. Taken together, these findings identify RNA fragments that induce TA-mediated paralog upregulation and suggest antisense transcripts targeting via homology-based pairing as a driver of TA.

### Trigger screens identify self-activating sequences

**Fig. 4.**
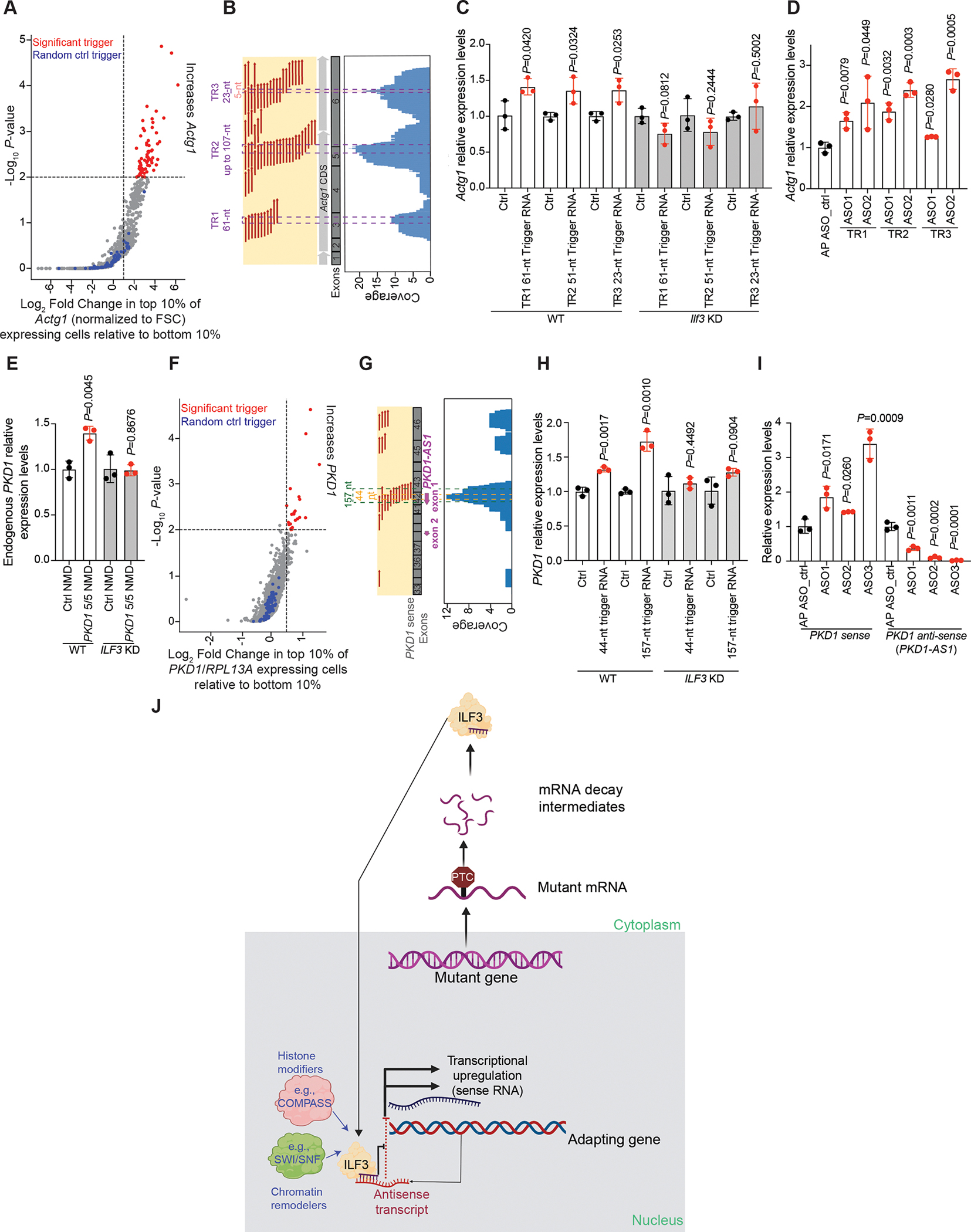
Trigger screens identify regions of mRNA sufficient for self-TA. (**A**) Enrichment of triggers in the top 10% of *Actg1* (as inferred by NeonGreen (NG) signal) expressing cells, normalized to forward scatter (FSC), relative to the bottom 10% of expressing cells, plotted against one-sided MAGeCK-calculated *P* values obtained from two independent replicates of the trigger screen. Red dots represent triggers that significantly increased *Actg1* expression (Log_2_ Fold Change >=1, P-value ≤0.01), while blue dots represent the control random scrambled triggers showing that none of them scored as significant. (**B**) Location of the significant triggers identified from the trigger screen mapped onto *Actg1* mRNA sequence. Three major trigger regions (TR) where identified of different lengths. Bottom plot shows coverage of the significant triggers at each nucleotide within *Actg1* cDNA. (**C**) qPCR analysis of *Actg1* mRNA expression levels in WT (white bars) or *Ilf3* knockdown (grey bars) MEFs transfected with the indicated trigger RNAs relative to cells transfected with control RNAs of the same length. (**D**) qPCR analysis of *Actg1* mRNA expression levels in MEFs transfected with the indicated ASOs targeting antisense RNAs within the different TRs. (**E**) qPCR analysis of *PKD1* expression levels in WT (white bars) or *ILF3* knockdown (kd) (grey bars) HEKs expressing the last fifth of the *PKD1* coding sequence, or a control (GFP-2A-RFP) message from the NMD vector. (**F**) Enrichment of triggers in the top 10% of *PKD1*/*RPL13A* expressing cells, relative to the bottom 10% of expressing cells, plotted against one-sided MAGeCK-calculated *P* values obtained from two independent replicates of the trigger screen. Red dots represent triggers that significantly increased *PKD1* expression (Log_2_ Fold Change >=0.5, P-value ≤0.01), while blue dots represent the control random scrambled triggers showing that none of them scored as significant. (**G**) Location of the significant triggers identified from the trigger screen mapped onto the last fifth of *PKD1* coding sequence. A single trigger region was identified that mapped exactly at exon 1 of an annotated antisense RNA at *PKD1* locus. Bottom plot shows coverage of the significant triggers at each nucleotide within the last fifth of *PKD1* coding sequence. (**H**) qPCR analysis of *PKD1* mRNA expression levels in WT (white bars) or *ILF3* knockdown (grey bars) HEKs transfected with the indicated trigger RNAs. The 157-nt trigger RNA experiment was repeated five times and consistently led to an upregulation of *PKD1* except in a single experiment. (**I**) qPCR analysis of *PKD1* mRNA and *PKD1* antisense RNA expression levels in HEKs transfected with the indicated ASOs targeting *PKD1* antisense RNA. (**J**) A model for ILF3-dependent transcriptional adaptation. ILF3 binds to mRNA decay intermediates and translocates back to the nucleus, where it is guided to the adapting genes’ locus via interaction with antisense RNAs at the locus. Once localized at a given locus, ILF3 may recruit transcriptional regulators that enhance expression of the adapting gene directly (e.g., by enhancing transcriptional elongation) or indirectly by modifying the chromatin environment. In addition, base-pairing with antisense RNAs may itself interfere (black T-bar) with the antisense RNAs’ negative effects (dashed red T-bar) on sense gene expression, thereby also contributing to increased gene expression. However, TA effects may also arise if decay intermediates exhibit sequence homology and bind to other RNAs, such as regulatory RNAs including eRNAs or promoter-associated RNAs, or even sense pre-mRNAs. In such cases, ILF3 recruitment could promote gene expression through a variety of potential mechanisms (see Supplementary Discussion, fig. S12). Figure created with BioRender.com. (**B, G**) Left Figure made in Snapgene. (**C, D, E, H, I)** n = 3 biologically independent samples. Control expression levels were set at 1 for each assay. Data are mean ± s.d., and a two-tailed Student’s t-test was used to calculate *P* values.

Genes harboring PTCs can display elevated pre-mRNA levels (92). Consistently, TA has also been shown to increase expression of the gene from which the trigger originates—a phenomenon termed ‘self-TA’ (13, 14, 23, 26, 66)—which may be particularly beneficial for upregulating the wild-type allele in cases of heterozygous mutations, as it can lead to functional compensation. Notably, we observed increased ILF3 enrichment at RNAs originating from the perturbed gene’s locus in mutant cells (Fig. 2B, fig. S7G). Consistent with this observation, and as previously noted, endogenous *Actg1* was upregulated in MEFs transduced with the *Actg1* NMD vector (Fig. 3A). We therefore asked whether trigger screens could also identify regions that mediate self-TA. To this end, we generated a NeonGreen (NG) reporter cell line for endogenous ACTG1 through a knockin approach (93) (fig. S11A, Methods), and performed a trigger screen using the *Actg1*-tiling library to identify sequences capable of activating endogenous *Actg1*. Notably, hits from the screen clustered into three regions across the *Actg1* transcript (Fig. 4A, B), hereafter referred to as Trigger Regions (TR). TR1 showed a distinct peak at a defined 61-nt region in exon 2. TR2 was broader and spanned up to 107 nt across exons 5 and 6. TR3 was sharply defined, peaking within a 5-nt stretch in the 3′ UTR of exon 6. To identify potential trigger RNAs, we selected the 61-nt region corresponding to the peak in TR1 and found that its transfection induced *Actg1* expression (Fig. 4C). For TR2, given the absence of a distinct peak, we turned to our native ILF3 nuclear RIP-seq data and tested five RNAs from this region, one of which, a 51-nt fragment, appeared to be sufficient to activate *Actg1* expression upon transfection (fig. S11B, Fig. 4C). For TR3, we expanded the 5-nt peak observed in the screen to a 23-nt region to generate a testable RNA fragment, which also induced *Actg1* expression (Fig. 4B, C). Notably, each of these RNAs led to increased *Actg1* expression in an ILF3-dependent manner (Fig. 4C). Accordingly, recruitment of dCas13-NF110 to antisense RNAs within each of the three trigger regions led to upregulation of *Actg1* (fig. S11C). Similarly, transfection of gapmer ASOs targeting antisense RNAs in these regions induced *Actg1* expression (Fig. 4D).

To determine whether trigger screens can identify trigger sequences outside the gamma-actin family, we tested *Rela*, a previously reported TA model (13). Expressing *Rela* coding sequence (CDS) from the NMD vector in MEFs led to increased endogenous *Rela* expression in a ILF3, and NMD-dependent manner (fig. S11D, E). We therefore cloned a new trigger screen library tiling *Rela* CDS into the NMD vector, and used it to screen for sequences capable of activating endogenous *Rela* using FLOW-FISH. To distinguish screen-derived transcripts from endogenous *Rela*, we used FLOW-FISH probes targeting *Rela* UTRs, which are not present in the library, and confirmed that these probes detect *Rela* mRNA (fig. S11F, G). Oligos that scored as hits clustered within a defined 187-nt region within exon 4 (fig. S11H, I). However, transfection of a corresponding 187-nt RNA did not induce *Rela* expression (fig. S11J). We therefore reasoned that smaller RNAs within this region might serve as more effective triggers. Using our native ILF3 nuclear RIP-seq data, we selected seven RNAs mapping to the identified trigger region and found that four of them appeared to induce endogenous *Rela* expression upon transfection into MEFs, in an ILF3-dependent manner (fig. S11K). As with other models, a gRNA targeting dCas13-NF110 to antisense RNAs within the trigger region led to *Rela* upregulation (fig. S11L), and gapmer ASOs targeting these antisense RNAs similarly increased *Rela* expression (fig. S11M).

Next, we sought to apply the trigger screen approach to a clinically relevant gene, *PKD1*, whose haploinsufficiency can cause polycystic kidney disease and may benefit from strategies that upregulate the WT allele (94). Due to its length (~14 kb), we divided *PKD1* CDS into five fragments, cloned each into the NMD vector, and tested their ability to upregulate endogenous *PKD1* in HEK cells. Notably, the fifth (most 3′) fragment induced the strongest *PKD1* expression, in an NMD, and ILF3-dependent manner (Fig. 4E, fig. S11N–P). We therefore cloned a trigger screen library tiling this last fifth region into the NMD vector and screened for sequences activating *PKD1* using FLOW-FISH probes targeting upstream regions, which we confirmed detect *PKD1* mRNA (fig. S11Q, R). Oligos that scored as hits clustered within a defined 44-nt region in exon 42 of *PKD1* mRNA (Fig. 4F, G). This region overlapped with exon 1 of an annotated antisense RNA (*PKD1-AS1*), further supporting a mechanistic link between trigger RNA activity and antisense transcripts targeting (Fig. 4G). This interaction, however, likely involves the *PKD1-AS1* pre-mRNA rather than the mature transcript since the pre-mRNA is more likely to be in proximity to chromatin, whereas the mature spliced transcript may rapidly diffuse away. Transfection of the corresponding 44-nt trigger RNA modestly upregulated *PKD1* in an ILF3-dependent manner (Fig. 4H). The limited activity could be partly due to its short length and high GC content (82%), which may contribute to suboptimal targeting efficiency. We therefore extended the trigger region to 157-nt around the peak (Fig. 4G), which induced stronger *PKD1* upregulation in an ILF3-dependent manner (Fig. 4H). Notably, ASO-mediated knockdown of the overlapping antisense RNA (*PKD1-AS1*) led to increased *PKD1* expression (Fig. 4I). We also observed that each trigger RNA appeared to activate its cognate target with higher specificity (fig. S11S). Taken together, these findings support the use of trigger screens to identify self-activating trigger sequences and provide further evidence of antisense transcripts targeting as an important driver of TA.

## Discussion

Here, we identify ILF3 as an RBP connecting mRNA decay and transcriptional activation during TA in both mouse and human cells, and through two modes of mutant mRNA decay: NSD and NMD. We propose a working model in which ILF3 binds to mRNA decay intermediates which, upon translocation to the nucleus, can guide it to the adapting genes’ locus through sequence complementarity to antisense RNAs at those loci. At the adapting genes’ locus, ILF3 can lead to increased gene expression through recruiting epigenetic modifiers (Fig. 4J). In some cases, it is possible that base-pairing with antisense RNAs may itself interfere with their negative effects on sense gene expression, thereby also contributing to increased gene expression (68–90) (Fig. 4J). We also expand the molecular understanding of TA by showing that it may involve enhanced transcriptional elongation. We propose two main requirements for ILF3-dependent TA: (a) mRNA decay intermediates that may originate from NMD (UPF1-dependent) or from NSD (involving PELO) (13, 16, 17, 23) and that can bind ILF3; and (b) antisense RNAs at the adapting gene’s locus that exhibit complementarity to the decay intermediates. Our screens identified trigger RNAs of different sizes, and future studies will help define the size, sequence, and potential structural constraints of TA-inducing decay intermediates. Other translation-independent RNA decay processes, such as Cas13-mediated cleavage or ribozyme-driven nuclear decay of pre-mRNAs (23, 26), have also been reported to induce TA-like effects, though a role for ILF3 remains to be tested. Translation-dependent decay pathways, such as NGD, would also be expected to trigger TA, although it likewise remains to be tested. While we focus in this study on antisense RNA targeting—implicated in both paralogous and self-TA—it is also possible that TA may arise from sequence homology to other RNAs, including promoter- or enhancer-associated transcripts. We outline a working model for this broader possibility that warrants further investigation (fig. S12, Supplementary Discussion).

The trigger screen’s identification of trigger sequences shows that targeting key anti-sense transcripts within a narrow region of a gene can be sufficient for modulating the expression of a gene. This strategy opens up the possibility that short trigger RNAs, or ASOs, can function as a novel therapeutic strategy for upregulating gene expression. Future trigger screens will enable better design and prediction of trigger RNAs across a range of clinically-relevant genes and will enhance our understanding of the features of trigger RNAs and their targets influencing the response (Supplementary Discussion). Future studies may also shed light on whether certain RNA fragments need to be co-loaded onto ILF3 during the process of mRNA decay, which may explain why some RNAs failed to induce TA when transfected into cells (fig. S10I, S11B, J, K).

The increased gene expression upon targeting dCas13-NF110 to antisense RNAs opens up the question of what other RBPs can augment gene expression, and whether they, or their associated RNAs, can be engineered as gene-activation tools. Finally, several endogenous transcripts that originate from alternative splicing events are substrates of NMD (95), and previous studies have reported that in WT cells, mRNA decay and gene expression are coupled processes (96–99). It is of interest to define if TA and ILF3 are involved in such processes and the extent to which they may be important to maintain stoichiometric expression ratios for some genes with sequence similarity under homeostasis.

## Materials and Methods

### Cell culture

MEFs were cultured in DMEM (Thermo) supplemented with 10% bovine calf serum (HyClone), 100 U/ml penicillin and 100 μg/ml streptomycin. K562 cells were grown in RPMI-1640 with 25 mM HEPES, 2.0 g/l NaHCO3, and 0.3 g/l L-glutamine supplemented with 10% FBS (or Tet System Approved FBS), 2 mM glutamine, 100 U/ml penicillin, and 100 μg/ml streptomycin. K562s were maintained at a confluency between 0.25 × 10^6^ to 1 × 10^6^ cells/mL. HEK293T/17 cells grown in DMEM supplemented with 10% FBS, 100 U/ml penicillin and 100 μg/ml streptomycin. All cells were grown at 37 °C, 95% humidity with 5% CO2.

### Lentivirus production

Lentivirus was produced by co-transfecting HEK293T/17 cells with the desired transfer plasmids (or pooled library) and two packaging vectors (psPAX2 and pMD2.G, Addgene #12260 and #12259) using TransIT-LTI Transfection Reagent (Mirus, MIR 2306) or Fugene HD transfection reagent. 6–14 hours post transfection, fresh media was added to the HEK cells, and virus was collected through collecting the media supernatant at 48 hours post transfection then flash frozen. For the NMD vectors, due to the low titers obtained from vectors with antisense transcription (100), ViralBoost Reagent (Alstem) was added to HEK cells 24 hours post transfection to increase viral production. In all instances, virus was rapidly thawed prior to transfection. Virus for the different screens was also generated using this method.

### Plasmids and cell line generation

#### Cloning

For rescue and overexpression experiments, cDNAs for *Actg1*, the two *Ilf3* isoforms (NF90 and NF110), along with the different NF110 domain deletion variants, and *Rnaseh1* were amplified from MEF cDNA. PCR fragments were cloned into a modified pLX_TRC209 lentiviral vector (101) (in which the hygromycin resistance cassette was replaced with a blasticidin resistance gene) using Gibson assembly (NEBuilder HiFi DNA Assembly Master Mix, NEB), between the BamHI and MluI restriction sites. NF110 deletion domains were generated by amplifying two PCR fragments flanking the region to be deleted and assembling them into the lentivector using Gibson assembly. To create 5′ FLAG-tagged versions of NF90, NF110, and their deletion variants, a FLAG-coding sequence (GACTACAAAGACGATGACGATAAGGAAGAAGTAAAA) was incorporated into the forward primer during PCR amplification from MEF cDNA. For FLAG-LacZ, two gene blocks encoding it were synthesized by IDT and cloned using a three-piece Gibson assembly strategy. To express NF90, NF110, and the different NF110 domain deletion variants under the control of *Ilf3* endogenous promoter, a 3 kb sequence upstream of *Ilf3* transcription start site was synthesized (Genewiz) and cloned by Gibson assembly between the SpeI and MluI sites of the pGM265 vector (Addgene #220179) (102), replacing the UCOE-EF1α promoter. The coding sequences for NF90, NF110, and NF110 deletion variants were then amplified and inserted downstream of the endogenous promoter, preceded by a BFP-P2A sequence, and cloned between MluI and SbfI sites using a three-piece Gibson assembly.

To clone the dCas13-NF110 plasmid, the NF110 isoform of *Ilf3* was amplified from mouse cDNA and cloned with an upstream XTEN80 linker, using Gibson assembly, downstream of dCas13 in the pXR002 plasmid (Addgene # 109050) that was digested with NheI and treated with antarctic phosphatase (NEB). To clone dCas13-lacZ, two gblocks encoding XTEN80-lacZ were ordered from IDT and cloned using a three-piece Gibson assembly, downstream of dCas13 in the pXR002 plasmid. The parental pXR002 plasmid was used to generate dCas13-Ctrl control cells expressing just dCas13. The NMD vector was generated through cloning GFP-2A-RFP and the downstream *HBB* NMD2 cassette from the NMD2 vector with a GCCACC kozak sequence upstream (103) into the pTREX vector ((100), gift from Bryan Cullen, Duke university) that was digested by AgeI and XbaI. The resulting plasmid allowed for expression of an NMD substrate as described in (103), and rTTA3 from the same vector. The No-NMD vector was cloned using a similar strategy by cloning GFP-2A-RFP without the downstream PTC-containing *HBB* exons and introns, that thereby wouldn’t elicit NMD, from the control vectors in (103) into the pTREX vector. To clone *Actg1* or *Rela* coding sequence, the five different parts of *PKD1* coding sequence into either vector, the respective sequences were amplified from cDNA of MEFs or HEKs, or ordered as gBlocks from IDT, and cloned between AgeI and NotI restriction sites using Gibson assembly. Full coding sequences were designed to have a stop codon that truncate the open reading frame (to avoid overexpressing the protein as NMD is not 100% efficient). We note that while we identified ORFs that when cloned into the NMD vector led to TA (including the ones reported in this study and other unpublished examples), some other tested genes did not elicit a detectable response. These include *HBB*, which did not induce its paralogs *HBG1*/*2* or itself in HUDEP-2 cells; *PKD1* (split into 5 pieces), which did not induce its paralog *PKD2* in HEK cells; *Dmd* (split into 4 pieces), which did not induce its paralog *Utrn* or itself in undifferentiated C2C12 cells; and *ARG1*, which did not induce its paralog *ARG2* or itself in HepG2 cells. While these genes do not appear to be subject to TA in the tested cell lines, we cannot rule out that they may display TA in other cell lines if conditions necessary for TA are absent in the tested lines but present in others, such as antisense transcription. For example, *DMD* transgenes designed to undergo NMD were reported to induce TA in HEK and HAP1 cells, but less so in HeLa cells (23).

#### Generation of stable cell lines

To generate stable cell transgenic cell lines, lentivirus was made from respective constructs and infected to cells at a multiplicity of infection (MOI) of 0.3 by adding it to media following splitting of MEFs, or using spinfection of K562s by centrifuging cells with virus containing media at 33°C for 1–2 hours at 1000×g to transduce K562s. 48 to 72 hours post infection, cells were treated with an antibiotic for 2 to 3 days to select for successfully transduced cells, or sorted based on a fluorescent protein. For MEFs, Puromycin was used at a concentration of 5 μg/ml, and Blasticidin (Thermo) at 10 ug/ml. For K562s and HEKs, Puromycin (Sigma) was used at a concentration of 1 μg/ml. For selection of cells generated by vectors encoding a fluorescent protein rather than a resistance marker, cells were sorted at 72–96 hours post transduction using a Sony Cell Sorter (SH800S). 100,000 cells were at least sorted when generating polyclonal stable cell lines. The following cell lines were used, or generated using the indicated plasmids, for multiple experiments: *Actg1*-NSD MEFs rescued with full length transgenic *Actg1* cDNA (and western blot assays confirmed rescue); WT and *Actg1*-NSD, rescued or not-rescued, MEFs expressing Cas9-T2A-EGFP under an Ef1a promoter (Gift from Dian Yang), or ZIM3 KRAB-dCas9-P2A-EGFP (Addgene #188899); K562 Cas9-BFP expressing cells (104).

#### Generation of knockout and knockdown cell lines

Cas9 gRNA plasmids were generated by annealed oligo cloning of top and bottom oligonucleotides (IDT) into a lentiviral pU6-sgRNA EF-1α-Puro-T2A-BFP vector between BstXI and BlpI sites (Addgene #84832). For knock down with CRISPRi, we used a programmed dual sgRNA guide vector ((105); Addgene #140096) to increase the efficiency of knockdown. A dual gRNA approach was also used to clone the gRNAs used to target *DDX21* and *CSNK1E* in K562s, and *Rnaseh1* in MEFs for Cas9 KOs. To generate *UPF1* knockouts in K562s a dual gRNA was cloned into a modified dual gRNA vector where mCherry was the selection marker. 72 hours post transduction, cells were sorted based on mCherry expression and transduced with virus encoding gRNAs targeting *DDX21* or *CSNK1E*, or non-targeting gRNAs. To generate *ILF3*, *SRP9* and *SERBP1* knockouts in K562s, four Cas12 gRNAs were cloned using golden-gate assembly, into an all-in-one Cas12a expression lentivector (Addgene #203398) that was modified to replace the puromycin selection marker with mCherry. To generate *ILF3* knockout in HEK cells, the same all-in-one Cas12 vector containing *ILF3*-targeting gRNAs was used. Experiments were performed on day 8 post-transduction, and ILF3 siRNA (Horizon Discovery, 012442–00-0005) was transfected on day 6. This early time point and siRNA treatment was necessary to achieve efficient ILF3 depletion, as the high proliferation rate of HEK cells—combined with the essential role of ILF3 in their growth—led to preferential expansion of cells in which ILF3 was not effectively knocked out (e.g., due to in-frame mutations). gRNAs used to generate a knockout was chosen as the top 1 or 2 gRNAs from the mouse CRISPR knockout Gouda library (106) or the human Brunello library (107), or the human Cas12 Inzolia library (108), while those used to knockdown mouse gene expression were chosen as the top 1 or 2 gRNAs in the mouse CRISPRi-v2 CRISPRi library (51). For arrayed experiments, lentiviruses used to overexpress an ORF were used at an MOI of 0.3 while arrayed gRNA validation experiments for Cas9 or Cas13 were used at higher MOIs. To generate monoclonal *Actg1*-NSD;Δ*Ilf3* MEFs, two gRNAs were designed using the CRISPR design tool CHOPCHOP (http://chopchop.cbu.uib.no/ (109)) and cloned into two modified pX458 plasmids expressing either ECFP (Addgene #112220) or DsRed2 (Addgene #112219) as previously described (110). MEFs were then electroporated using Nucleofection (Lonza) with 5 μg of each of the two cloned plasmids, according to the manufacturer’s protocol. Two days after transfection, cells expressing ECFP and DsRed2 were subjected to single-cell sorting into 96-well plates using a FACSAria II (BD Biosciences). Three to four weeks after single-cell sorting, clones were isolated and genotyped by Western blot and/or PCR and sequencing. Two *Actg1*-NSD;Δ*Ilf3* knockout clones were obtained (clone 1 and 2; Fig. 1C, fig. S1D). Further experiments were done with clone 1. Although *Actg1*-NSD;Δ*Ilf3* cells exhibited reduced *Actg1* transcription (fig. S1F), we tested CRISPRa-mediated activation of *Actg1* in those cells and found that it did not increase *Actg2* expression, indicating that ILF3 loss, rather than diminished transcription of the mutant transcript and consequent reduction in decay intermediates, underlies the observed decrease in *Actg2* levels. Independently, we generated four additional *Ilf3* knockout clones in a heterozygous *Actg1*-NSD cell line (which also strongly upregulates *Actg2*) and observed a consistent decrease in *Actg2* and *Actg1* expression levels in these clones. A similar approach was used to generate the *Actg1*-NSDΔ75 using two gRNAs, one upstream and the other downstream of the 75-nt trigger sequence of *Actg1* that deleted exon 4 containing the 75-nt trigger region. We were only able to generate just one clone with a homozygous deletion of the *Actg1*-NSDΔ75 genotype. To generate WT;Δ*Ilf3*, a dual Cas9 gRNA targeting *Ilf3* was cloned into the pU6-sgRNA EF-1α-Puro-T2A-BFP and used to make lentivirus that were then transduced into WT-Cas9 cells. Three days after transduction, cells expressing BFP were subjected to single-cell sorting into 96-well plates. We also generated three *Ilf3* knockout clones in *Actg1* full-locus deletion cells that, like in WT cells, did not affect *Actg2* or *Actg1* expression levels. We also generated three additional WT;Δ*Ilf3* clones besides the one reported in Fig. 1D that also did not display changes in *Actg1* or *Actg2* levels. We note that while multiple clones were generated to rule out clone-specific effects on *Actg1* or *Actg2* expression, the more expensive sequencing experiments were performed on a single clone; as a result, some gene-specific responses in those experiments may vary between clones, but the overall conclusions are expected to be consistent. Cas13 gRNAs were cloned into pLentiRNAGuide_00 between BsmBI sites (Addgene #138150). Cas13 gRNAs were designed through https://cas13design.nygenome.org/ (111). Cas9, Cas12 and Cas13 gRNA sequences used are listed in table S1.

#### Generation of Actg1-NG11 Knock-in (KI) Cell Line

W we generated a NeonGreen (NG) reporter cell line for endogenous ACTG1 by knocking in (93) *NG11* sequence immediately downstream of the start codon, while stably expressing *NG1–10* from a separate transgene. Specifically, prior to generating the *Actg1*-*NG11* knock-in (KI) cell line, *NG1–10* was first cloned into a modified pLX_TRC209 lentiviral vector (101) (in which the hygromycin resistance cassette was replaced with a blasticidin resistance gene) and used to make lentivirus that was transduced into WT MEFs to establish a cell line that stably expresses NG1–10. *NG11* was then knocked into the *Actg1* locus immediately downstream of the start codon in exon 2, followed by a GS linker, as previously described (93). Briefly, a gRNA targeting a region near the start codon was cloned into PX330 (Addgene #42230) (112). A donor vector containing *NG11* with homology arms was constructed by amplifying 900 nucleotides of the left homology arm (LHA) upstream, and 900 nucleotides of the right homology arm (RHA) downstream of the intended knock-in site, from genomic DNA. Silent mutations were introduced into the gRNA PAM sequence within the RHA to prevent re-cutting. The LHA and RHA were cloned into the pUC57 vector (Thermo Fisher) between BamHI and XbaI sites using Gibson Assembly. A total of 5 μg each of the PX330-gRNA construct and the donor vector were co-nucleofected into NG1–10-expressing MEFs following the manufacturer’s instructions. Three days post-transfection, cells expressing NeonGreen were single-cell sorted into 96-well plates using a FACSAria II (BD Biosciences). Two to three weeks after sorting, clones were expanded and genotyped using primers flanking the knock-in region to identify those with a homozygous *NG11* insertion. We chose the split NeonGreen system to minimize the size of the knock-in at the *Actg1* locus, reducing the risk of perturbing local transcriptional regulation or interfering with transcriptional adaptation at this gene.

### qPCR expression analysis

qPCR was performed in a QuantStudio 7 Real-Time PCR System (Applied Biosystems). RNA was isolated using TRIzol and at least 500 ng RNA was used for reverse transcription using the Maxima First Strand cDNA synthesis kit (Thermo). All reactions were performed in at least technical duplicates and the results represent biological triplicates. Primers were designed using PrimerBlast (https://www.ncbi.nlm.nih.gov/tools/primer-blast/). Primers used to detect the *Actg1* NMD and no-NMD transgene were designed where the forward primer bind to the end of the *Actg1* sequence and the reverse primer binding to exon 2 of *HBB* in the NMD2 cassette, while for the no-NMD vector the reverse primers was designed to bind the junction of *Actg1* and a vector-specific downstream sequence. All experiments involving the use of lentivectors encoding gRNAs performed at day 8 post transduction. Primers used to detect endogenous *Actg1* or *Rela* in cells transduced with *Actg1* or *Rela* NMD or no-NMD vectors were designed in the UTRs to avoid amplifying the transgenic *Actg1* or *Rela* which was only the coding sequence. For *PKD1*, primers within the coding sequence that did not match the region cloned into the NMD or no-NMD vectors were used. Worth noting is that some gRNAs that led to a strong level of *Actg2* upregulation with dCas13-NF110 (or sometimes dCas13 alone) led to slower cell proliferation relative to control gRNAs, a phenotype that could be due to strong upregulation of *Actg2* in the presence of WT *Actg1* as previously reported (113), or potential off-target effects of the individual gRNAs. For some cases, we also observed loss of *Actg2* upregulation upon culturing the cells for longer periods of time until the growth phenotype has been resolved. It likely reflects silencing of the dCas13 or gRNA expression, or cells with weaker expression outcompeting those with strong *Actg2* upregulation. For the NMD and no-NMD vectors initial experiments, cells were cultured in standard FBS or BCS and treated for 72 hours with 2μg/ml doxycycline hyclate (Sigma) following the end of puromycin selection before extracting RNA for qPCR analysis. In the initial experiments, we observed a consistent 1.5- to 1.7-fold upregulation of *Actg2* with the *Actg1* NMD vector (Fig. 3A). However, when the stable *Actg1* NMD cells were maintained in culture for several weeks and the experiment was repeated using Tet system–approved FBS (Thermo) prior to doxycycline induction, we observed a stronger 3- to 4-fold upregulation of *Actg2*. Independently from this study, while working on a project investigating the specificity of trigger RNAs, RNA sequencing revealed that transfection of the *Actg2* 75-nt trigger RNA elicited an immune response in WT cells. Although such a response is expected when introducing foreign RNAs into cells, transfection of poly(I:C) did not induce *Actg2* expression (and instead reduced it). RNA sequencing also showed that the 75-nt trigger RNA also elicited an immune response in *Ilf3* knockdown cells, which did not upregulate *Actg2*. Further disconnecting immune activation from *Actg2* upregulation, RNA sequencing of WT cells transfected with the shorter 24+27+31 RNAs (Fig. 3F), which did upregulate *Actg2*, showed no signs of immune activation. Together, these results ruled out an association between immune responses and *Actg2* upregulation. Future studies will aim to design trigger RNAs with reduced immunogenicity. We also observed that the *Actg2* 75-nt trigger RNA activated *Actg2* expression in SRF (a transcription factor regulated by actin dynamics) polyclonal knockout MEFs, supporting that the trigger’s activity is independent of ACTG1 protein loss. Equal numbers of cells were seeded in 12 or 24 well plates 48 or 72 hours prior to RNA extraction for all qPCR experiments except those that involved nucleofection of ASOs or trigger RNAs where cells were plated in 96 well plates following nucleofection. For K562s, cells were split to be at a concentration of 250,000 cells/ml a day before they were collected for RNA extraction and qPCR. For experiments involving *DDX21*, the top 3 TA-candidate adapting were selected as protein-coding genes that exhibited the highest upregulation levels upon CRISPRn-perturbation but not upregulated upon CRISPRi-mediated perturbation (Pvalue ≤0.05). *Rpl13a* and *Hprt* were used as housekeeping genes to normalize the experiments in mouse cells, and *ACTB* and *HPRT1* were also used to normalize qPCRs done on human cells. Fold changes were calculated using the 2−ΔΔCt method. Primer sequences used for the qPCR experiments are listed in table S2.

### Flow-FISH

Flow-FISH was performed according to the manufacturer’s protocol using the PrimeFlow^™^ RNA Assay kit (Invitrogen) with Alexa Fluor^™^ 647 (AF647) *Actg2* or *Rela* or *PKD1* probes and Alexa Fluor^™^ 750 (AF750) probes for *Rpl13a* (for MEFs) and *RPL13A* (for HEKs) as housekeeping genes that was used to normalize for cell size. The following modifications were made to the protocol: 20 million cells per 15-ml tube cells were fixed with in a 10 ml solution of 3.7% Formaldehyde for 10 minutes followed by permeabilization with 70% Ethanol for 1–2 hours at 4°C as per the Stellaris RNA FISH protocol for cells in suspension (BioSearch Technologies). Fixed and permeabilized cells were then split across 1.5 ml tubes with 5 million cells in each and proceeding according to the PrimeFlow protocol except that the incubation with the Target probes was performed overnight rather than 2 hours. Following completion of the protocol, cells were sorted based on A657 to A750 ratio, and the top and bottom 30%, or 10%, were sorted for the CRISPR screens, or the trigger screens, respectively. Cells were then centrifuged at 800g for 5 minutes and frozen at −80°C until gDNA isolation. Probes were tested for specificity prior to its use in each screen. For *Actg2* probes it was tested in WT and *Actg1*-NSD cells where *Actg2* is upregulated. For *Rela,* the probes were tested in WT MEFS or MEFs that were transduced with an all-in-one CRISPRi vector encoding Zim3_KRAB-dCas9 and two CRISPRi gRNAs targeting *Rela* to knockdown its expression. The *PKD1* probes were tested in WT HEK cells, or HEK cells that were transduced with an all-in-one CRISPRi vector encoding Zim3_KRAB-dCas9 and two CRISPRi gRNAs targeting *PKD1* and that was also simultaneously transfected with *PKD1* siRNA (Santa Cruz Biotechnology, sc-40861) to further knockdown its expression.

### CRISPR screen

The compact Mouse CRISPR Knockout Pooled Library (Gouda (106); two gRNAs per gene, Addgene Pooled Library #136987) was transduced to 150 million cells (and thereby maintaining a 1000x coverage of each library element post selection) of the rescued *Actg1*-NSD and WT MEFs cells expressing Cas9-GFP at an MOI of 0.3. Transduction was performed in the presence of 8 μg/ml Polybrene (EMD Millipore), and cells were grown in multiple 15 cm dishes. 48 hours after infection with the genome-wide library, guide positive cells were selected with 5 μg/mL puromycin for three days. At day 8 post infection, 200 million cells were collected and stained with *Actg2* and *Rpl13a* probes as per the Flow-FISH protocol. Following completion of the protocol, cells were sorted based on A657:A750 ratio, and the top and bottom 30% were sorted. Cells were then centrifuged at 800g for 5 minutes and flash frozen at −80°C until genomic DNA (gDNA) isolation. The screen in rescued *Actg1*-NSD cells was performed in duplicate while the WT counter screen was a single replicate. Prior to gDNA purification, cells were reverse cross-linked according to a modified ChIP reverse cross-linking protocol (114). gDNA was purified using the Nucleospin Blood XL kit (Takara Bio, #740950.10) and amplified by index PCR with barcoded primers. The resulting guide library (~354 bp) was purified using SPRIbeads (SPRIselect Beckman Coulter #B23318). Sequencing was performed using an Illumina HiSeq2500 high throughput sequencer. The MAGeCK tool (91) was then used to align sequencing reads to the Gouda library sequences, followed by counting, generation of gene level-enrichment scores and calculation of *P* values. The two replicates of the rescued *Actg1*-NSD screen were analyzed using the --paired option of the *mageck test* function. We note that the NMD factor *Upf1* was not identified as a hit in our screen, which is expected given that the screen was performed using an NSD model. The NSD factor *Pelo*, however, also did not score as a hit. To investigate this, we tested the two *Pelo*-targeting gRNAs included in the library and found that neither stabilized the mutant *Actg1*-NSD mRNA, and western blot analysis suggested they were not effective in knocking out the protein. However, the observation could also be due to that for some mutant mRNAs, compensatory mRNA decay pathways prevent stabilization of the transcript upon loss of another decay factor, thereby not affecting TA. For example, in two studies relying on NMD models, only dual knockdown of *Upf1* together with either *Smg6* or a component of the exosome complex was sufficient to stabilize the mutant transcript and abolish TA (13, 23). Notably, the decay factor *Cnot9* was identified only in the *Actg1*-NSD screen and not in the WT screen further supporting the importance of mRNA decay for TA. We also identified the ribosome recycling factor *Abce1*, which, in addition to its broad role in translation regulation, has been suggested to be involved in NSD (115), and was therefore highlighted in our analysis. However, it also scored as a hit in the WT screen. When we tested the *Abce1*-targeting gRNAs independently in WT and *Actg1*-NSD Cas9-expressing MEFs, we observed that most cells began dying as early as 4–5 days post-transduction, indicating that *Abce1* is a very essential gene in MEFs, consistent with its broad role as a ribosome recycling factor. In K562 cells, its DepMap (116) dependency score is −2.435, much higher than that of *UPF1* (−1.08), *CNOT9* (−0.56), and *ILF3* (−1.06). It is therefore possible that the observed effects are secondary effects, making assessment of its role in TA challenging, particularly given its broader functions beyond mRNA decay, which may be difficult to disentangle from TA-specific effects. We also identified nuclear RNA decay factors, including *Zc3h18* and *Skiv2l2*, suggesting that TA may be influenced by nuclear RNA decay, either directly, as supported by recent studies (23, 26), or indirectly through the degradation of repressive antisense transcripts. *Zc3h18* was identified only in the *Actg1*-NSD screen, while *Skiv2l2* also scored in WT cells. Further investigation will be needed to clarify the underlying mechanisms. Finally, we confirmed that components of the COMPASS complex are important for modulating *Actg2* expression in *Actg1*-NSD cells. While *Wdr5* also scored as a hit in the WT screen (which is not unexpected, as loss of H3K4me3 would generally reduce transcription), *Setd1a* was identified only in the *Actg1*-NSD screen, consistent with previous reports that identified the COMPASS complex to be important for certain TA models (13, 14). Furthermore, while our analysis focused on a subset of screen hits, other candidates, including RBPs that may act in sequence- or context-dependent ways, represent promising avenues for future exploration of TA mechanisms. In Figures 1B, S7H and S8A, genes scoring as significant hits in WT cells were removed from the genes list prior to using the *mageck test* function for analysis of the *Actg1*-NSD screen. Gene-level enrichment scores and *P* values for the screens are available in table S3.

### Trigger library design and cloning

The *Actg1*, *Rela*, and *PKD1*_5/5 trigger library oligo pool was generated by designing 237-nt oligonucleotides that tile the full mouse *Actg1* mRNA sequence (including the 5′ and 3′ UTRs), the coding sequence of *Rela*, or the final fifth of the *PKD1* coding sequence, respectively, with 1-nt increments. This resulted in a total of 1716, 1414, and 2410 trigger oligos for *Actg1*, *Rela*, and *PKD1*, respectively. For the Random control oligo pool, a random sequence was generated (http://www.faculty.ucr.edu/~mmaduro/random.htm) and oligos were designed to tile that sequence with 10nt increments and iteratively optimized to have minimal mapping to the mouse and human genome. The overall synthesized oligo pool (Twist Biosciences) consisting of the gene-specific trigger sequences and 86 unique control random triggers were ordered to have the following structure: 5’ – PCR adapter – AgeI motif (ACCGGT) – Kozak sequence (GCCACC) – start codon (ATG) – 237nt trigger – stop codon (TAA) – NotI motif (GCGGCCGC) – PCR adapter 3’ and were cloned into the NMD lentiviral vector. Library cloning was guided by the protocol for Cloning of Pooled sgRNAs into Lentiviral Vector from the Weissman lab (https://weissman.wi.mit.edu/resources/Pooled_CRISPR_Library_Cloning.pdf) with adaptations of restriction enzymes used for insert/vector digestion and using an E-Gel EX 2% Agarose followed by column purification using GeneJET MicroKit (Thermo Scientific) for insert purification instead of a polyacrylamide gels. For oligo pool amplification, NEB next ultra II Q5 master mix 2x (NEB) was used using the manufacturer’s recommended PCR conditions. Ligation of the amplified library to the digested NMD vector was performed using T4 DNA ligase (NEB) at 16°C for 16h, followed by ethanol precipitation overnight at −20°C. The ligation product was then electroporated to Endura DUOs Electrocompetent Cells (BioSearch technologies) which were then incubated in liquid culture for 14h at 37°C. The resulting library was purified using Plasmid Plus Maxi Kit (Qiagen) and amplified using staggered P5/P7 indexed primers for paired-end sequencing on Miseq (Illumina) to confirm its balance. Paired-end reads were trimmed to include only the trigger sequences (i.e. between the ATG start codon and TAA stop codon) using the cutadapt tool (https://cutadapt.readthedocs.io/en/stable/). The trigger sequences used in the screen are available in table S4.

### Trigger screen

WT MEFs (for *Actg2* and *Rela* screens), *Actg1*-NG11 K.I. MEFs (for *Actg1* screen) and HEK293T/17 (for *PKD1* screen) were transduced with the trigger library at an MOI of 0.3 in the presence of 8 μg/mL polybrene transfection reagent to have a coverage of at least 1000 cells per element. 72h post transduction, MEFs and HEKs were selected with 5 μg/ml, or 1 μg/ml Puromycin, respectively, for two to three days. Cells were always maintained a 1000x coverage of each library element. Following at least 48-hour recovery, cells were induced with 2μg/ml Doxycycline Hyclate (Sigma) and expanded in 15cm plates with daily media exchange and fresh doxycycline addition for 4 days. Cells at 10,000x coverage for each library element were then collected. For *Actg2*, *Rela* and *PKD1* screens, cells were collected and stained with *Actg2 or Rela* or *PKD1* probes that were conjugated to AF647 and *Rpl13a* (mouse) or *RPL13A* (human) probes that were conjugated to AF750 as housekeeping control genes as per the Flow-FISH protocol. *Actg2* probes spanned the entire mRNA sequence of *Actg2*. The *Rela* probes were designed to bind only the UTRs of *Rela* to avoid it from detecting the *Rela* sequences in the *Rela* trigger library. *PKD1* probes were probes that bound to regions upstream of the fifth part of *PKD1* coding sequence that was used in the screen. Following completion of the protocol, cells were sorted based on the A647:A750 ratio, and the top and bottom 10% were sorted. For the *Actg1* screen, cells were collected and directly sorted based on the Neon-Green(NG):Forward-Scatter(FSC) ratios, and the top and bottom 10% were sorted. Each screen was performed in duplicates. Cells were then centrifuged at 800g for 5 minutes and flash frozen at −80°C until genomic DNA (gDNA) isolation. Cells from *Actg2*, *Rela* and *PKD1* screens were reverse-crosslinked prior to gDNA isolation. gDNA was purified using the Nucleospin Blood L kit (Takara Bio) and amplified by index PCR with barcoded primers. The resulting library was purified using SPRIbeads (SPRIselect Beckman Coulter #B23318). Paired-end sequencing was performed using an Illumina MiSeq, or an Element Biosciences AVITI, sequencer using custom primers. A Python script (https://github.com/josephreplogle/CRISPRi-dual-sgRNA-screens; (105)) was used to align the reads to the trigger library sequences and quantifying them. The MAGeCK tool (91) was then used for generation of enrichment scores and calculation of *P* values. The two replicates of the screen were analyzed using the --paired option of the *mageck test* function. For the *PKD1* screen, oligos containing a NotI site within the trigger sequence were excluded from analysis, as they could not be properly cloned into the NMD reporter vector, which was digested with NotI as part of the cloning strategy. Trigger enrichment scores and *P* values are available in table S4. Triggers identified to significantly increase *Actg2*, *Actg1*, *Rela* and *PKD1* expression were then mapped to the relevant cDNA on the SnapGene software.

### Mutagenesis Screen

For the mutational scanning library, we designed a comprehensive set of 75-nt oligonucleotides based on the WT *Actg1* 75-nt trigger sequence: TGTTCGTGACATAAAGGAGAAGCTGTGCTATGTTGCCCTGGATTTTGAGCAAGAAAT GGCTACTGCTGCATCATC. This library included all possible single and double transversion mutations (A ↔ C and G ↔ T), as well as all adjacent tri- and tetra-nucleotide mutations, enabling systematic exploration of sequence homology requirements for transcriptional adaptation in the *Actg1*/*Actg2* model. Additionally, we included a set of control sequences derived from a random 75-nt control RNA: CAATTTCAGCCCTCTTATCCTCGGCGTTGTGTGTCAAGTGACGTAGACCTAGATTGAC TCTATGACGGTATCTGC. From this sequence, we generated 4 variants with one mismatch, 13 with two mismatches, 4 with three adjacent mismatches, and 4 with four adjacent mismatches. In total, the library contained 3,022 unique oligonucleotides. Those oligos were then cloned into the NMD vector, and used for a FLOW-FISH based screen as described above for the *Actg2* trigger screen.

### RIP-seq and qPCR

Cross-linked RIP was performed using the Magna Nuclear RIP (Cross-Linked) Nuclear RNA-Binding Protein Immunoprecipitation Kit (Millipore Sigma) while native RIP was performed using the Magna Nuclear RIP (Native) Nuclear RNA-Binding Protein Immunoprecipitation Kit (Millipore Sigma) according to the manufacturer’s protocol using at least 2× 10^7^ WT or rescued *Actg1*-NSD MEFs per replicate. Cross-linking was specifically performed to capture target RNAs hybridized to decay intermediates that associate indirectly with ILF3. For the cross-linked RIP, fixed nuclei were subjected to sonication using Bioruptor (Diagenode) prior to IP. Input samples were generated by reserving 10% of the starting lysate; the remaining volume was subjected to IP using the with 10 μg Anti-ILF3 antibody (abcam; ab92355 and BD Biosciences; Clone 21/DRBP76) coated onto protein A/G magnetic beads as described in the Magna RIP technical manual. RNA purified from both IP and input samples was concentrated by ethanol precipitation and resuspended in equivalent volumes of RNase-free water to be used to generate RNA-seq libraries. For the cross-linked total RNA RIP-seq, sequencing libraries were generated using 10 ng of RNA from input and IP samples using the SMARTer^®^ Stranded Total RNA-Seq Kit v2 - Pico Input Mammalian (Clontech). RNA sequencing was performed on a NovaSeq S1 instrument (Illumina), resulting in an average of 43 million reads per library, with 50 × 50 bp paired-end setup. Reads were then trimmed followed by mapping to the mouse reference genome GRCm38 (mm10), with gene annotations from Ensembl release 102. The number of reads aligning to genes were counted with featureCounts with the following parameters -*B -C -s 0 -t exon*, where only reads mapping at least partially inside exons were admitted, and these reads were aggregated per gene. Reads overlapping with multiple genes or aligning to multiple regions were excluded. Because the RIP-seq experiment was performed under crosslinking conditions—designed to capture target RNAs hybridized to decay intermediates and thus indirectly associated with ILF3—it may enrich both sense and antisense RNAs that are in close spatial proximity, base-paired as RNA duplexes (85, 87, 117), or co-localized within shared RNP hubs. To avoid bias toward one strand, we therefore analyzed the data shown in Fig. 2B in an unstranded manner (using the -*s 0* option in featureCounts). This choice is supported by prior work in which crosslinked RIP of dCas13—targeted to a specific mRNA via a guide RNA—identified trans-acting RNAs that physically interact with the mRNA itself rather than with dCas13 or its guide RNA directly (118). Unstranded analysis also reduces the likelihood of missing lowly expressed antisense RNAs that may not reach statistical significance despite enrichment, and avoids restricting detection to antisense transcripts typically associated with paralogous TA. It allows the identification of non-paralogous adapting genes, where decay intermediates may hybridize to sense pre-mRNAs at their loci. Supporting this rationale, strand-specific analysis (using -*s 1* or -*s 2* options of featureCounts) shows that most genes enriched in the unstranded data, including *Actg1* and *Actg2*, are also enriched when considering only the antisense or sense strand (fig. S7D). Experiments were done using three biological replicates. Differentially expressed ILF3 binding in rescued *Actg1*-NSD MEFs vs WT was identified using DESeq2 v.1.38.3 as described in https://support.bioconductor.org/p/61509/ in order to identify enrichments after normalizing IP signal to input. Genes were considered significantly enriched for ILF3 binding in *Actg1*-NSD cells relative to WT (the ILF3-enriched genes) if they met the following thresholds: baseMean > 30, log₂ fold change ≥ 1, and P value ≤ 0.01. *Actg1* had a log2 fold change of 0.9987 and was also included. This yielded 383 genes. Of these, 81 genes did not meet a Benjamini-Hochberg adjusted P value (Padj) threshold of ≤ 0.1. *Actg2* was among this group (Padj = 0.131), however it was independently validated by qPCR (Fig. 3E). We retained these genes in the analysis, as their exclusion did not affect the conclusions presented in Fig. 2— in fact, some conclusions were strengthened without them, but we opted to keep them to include *Actg2*. We further tested more stringent filtering strategies, including restricting to Padj ≤ 0.05 and excluding genes with low expression in WT cells (baseMean < 6) to avoid input bias. In both cases, exclusion of these gene subsets did not alter the conclusions, so all genes were retained in the final analysis. For small RNA native RIP-seq, 10ng of RNA was used to generate small RNA-seq libraries using the SMARTer smRNA-Seq Kit (Clonetech). RNA was treated with T4 Polynucleotide Kinase for 1 hr prior to library preparation to capture various potential mRNA decay intermediates that may not have a 5’P or 3’OH. RNA sequencing was performed on a NovaSeq S1 instrument (Illumina), with 150 × 150 bp paired-end setup (only Read 1, however, was used in downstream analysis). Cutadapt was then used to trim Read 1 using the following criteria *m 15 -u 3 -a AAAAAAAAAAAAAAA* as per the kit’s manufacturer’s recommendation followed by mapping to the mouse reference genome GRCm38 (mm10), with gene annotations from Ensembl release 102, as described above. We note that this native RIP was designed as an exploratory experiment to detect candidate locus-derived small RNAs that may associate with ILF3. Specificity of these associations remains to be determined in future experiments (e.g., by performing pulldowns in *ILF3* knockout cells). The generated BAM files were then used to identify RNAs associated with the trigger regions. Because the native RIP-seq was performed in *Actg1*-NSD cells lacking an mRNA-destabilizing *Rela* mutation, the identified RNAs tested in fig. S11K may be derived from normal decay of the wild-type *Rela* transcript. However, PRO-seq data showed that *Rela* transcription was not decreased in WT;Δ*Ilf3* relative to WT cells, suggesting that endogenous decay does not regulate *Rela* levels in WT cells. These observations may suggest that for the identified RNAs to possibly induce self-TA, higher levels—potentially generated through NMD or supplied via transfection—may be required to trigger *Rela* self-TA. For cross-linked RIP-qPCR, the RNA immunoprecipitated and input were used to make cDNA using the Maxima First Strand cDNA synthesis kit (Thermo), and qPCR was used to calculate % input for the different conditions tested.

### PRO-seq

PRO-seq (119) was performed in three different batches. The first was WT versus *Actg1*-NSD MEFs, the second was *Actg1*-NSD MEFs versus *Actg1*-NSD; Δ*Ilf3* cells, and the third was WT versus WT;Δ*Ilf3* cells. PRO-seq library preparation, sequencing and analysis was performed through the nascent transcriptomics core at Harvard Medical School. For each sample, we provided 2 million cells that were permeabilized, followed by library construction as described before (120). Batch 1 (WT versus *Actg1*-NSD MEFs) used an earlier version of the protocol that did not use UMIs, however a dual 6nt UMI was used for batch 2 and batch 3. Libraries were sequenced using an Illumina NovaSeq sequencer. Reads were processed and aligned to the mouse reference genome GRCm38 (mm10), with gene annotations from Ensembl release 102, as described previously (120). To select gene-level features for differential expression analysis, we applied a refinement of gene annotation (GGA) using PRO-seq and RNA-seq, assigning a single, dominant TSS and transcription end site (TES) to each active gene. This was accomplished using a custom script, get_gene_annotations.sh (available at https://github.com/AdelmanLab/GetGeneAnnotation_GGA), which uses RNA-seq read abundance and PRO-seq R2 reads (RNA 5’ ends) to identify dominant TSSs, and RNA-seq profiles to define most commonly used TESs. RNA-seq from WT and *Actg1*-NSD (13) cells and PRO-seq data from all conditions were used for this analysis, to comprehensively capture gene activity in the different samples. Differentially expressed genes were identified using DESeq2 (121) v.1.38.3. For comparisons between WT and *Actg1*-NSD cells, the deleted region of *Actg1* in *Actg1*-NSD cells was excluded to ensure a fair comparison.

### TT-seq and elongation index calculations

For each replicate, 10 million cells were seeded into two 15-cm dishes one day prior to the experiment. Two replicates were performed per genotype, resulting in a total of four plates per genotype. On the day of the experiment, one plate per genotype was processed at a time to avoid handling more than four plates simultaneously. The media was aspirated and replaced with fresh media containing 500 μM 4-thiouridine (4sU) for 20 minutes. Following incubation, the media was aspirated again, and cells were rapidly washed with PBS. Two milliliters of TRIzol were then added per plate, and the cells were scraped and collected into tubes, which were flash-frozen in liquid nitrogen. TRIzol samples were then handled to the Nascent Transcriptomics Core at Harvard Medical School which performed the TT-seq library preparation, sequencing and analysis as previously described (59, 122). Libraries were sequenced using an Illumina NovaSeq sequencer. Reads were processed and aligned to the mouse reference genome GRCm38 (mm10). Differentially expressed genes were identified using DESeq2 (121) v.1.38.3. As described previously (59), the elongation index was estimated using the ratio of normalized TT-seq to PRO-seq signals. Briefly, bedGraph files were first normalized to the spiked-in *Drosophila melanogaster* reads. An exception was made for batch 3, which was normalized to sequencing depth instead due to a twofold imbalance in PRO-seq spike-in read counts between experimental conditions. We confirmed that the choice of normalization method did not affect the conclusions. Following normalization, PRO-seq reads were summed in 500-nt bins using the make_heatmap script (https://github.com/AdelmanLab/NIH_scripts/tree/main/make_heatmap) with the -v t option. For TT-seq, mean per-nucleotide coverage was computed in the same 500-nt bins using the -v c option of the same script, then multiplied by 500 to obtain the mean coverage per bin. To ensure data quality, bins containing zero reads in any dataset, as well as those overlapping or downstream of the transcription end site (TES), were assigned a value of zero. The elongation index was calculated as the ratio of TT-seq to PRO-seq signal in each bin and the 15 bins (7.5 kb) downstream of the TSS were retained for further analysis. To assess changes in the elongation indices between ILF3-enriched genes and background in the different genotypes, we used two complementary statistical approaches: (i) a within-batch Mann–Whitney U test on log-transformed ratios of elongation index (Fig. 2I), and (ii) a multilevel model fitted across all batches. For all comparisons, only genes that were transcriptionally upregulated in *Actg1*-NSD cells relative to WT according to PRO-seq (log_2_(fold-change) > 0, p ≤ 0.05) were considered. For the first analysis, for each batch, consider two genotypes A and B (i.e. WT vs *Actg1*-NSD in batch 1, *Actg1*-NSD vs *Actg1*-NSD;Δ*Ilf3* in batch 2 and WT vs WT;Δ*Ilf3* in batch 3). For each bin, *j*, the ILF3-enrichment-dependent change in log_2_(fold-change) between genotypes can be written:

$$\Delta_{j}=\Delta_{j}^{\text{ILF3-enriched}}-\Delta_{j}^{\text{bg}},$$

where $\Delta_{j}^{\text{ILF3-enriched}}=\log_{2}\left({\text{EI}}_{A,j}^{\text{ILF3-enriched}}\right)-\log_{2}\left({\text{EI}}_{B,j}^{\text{ILF3-enriched}}\right),$ and $\Delta_{j}^{\text{bg}}=\log_{2}\left({\text{EI}}_{A,j}^{\text{bg}}\right)-\log_{2}\left({\text{EI}}_{B,j}^{\text{bg}}\right).$

Here, $\text{E}{\text{I}}_{\text{A,j}}^{\text{ILF3-enriched}}$ is the median elongation index in bin j for genotype A taken across transcriptionally upregulated ILF3-enriched genes, while $\Delta_{j}^{\text{bg}}$ is across background (bg) non-ILF3-enriched genes. For each batch, the Mann-Whitney U test was performed using ILF3-enriched and $\Delta^{bg}$ for all bins (see Fig. 2I for p-values). As an alternative statistical approach, the log-transformed elongation index, $y_{i}$, can be modelled using the following multilevel (123) approach:

$$y_{i}=N\left(X\beta +\gamma_{j\left[i\right]}+\delta_{k\left[i\right]}+\eta_{l\left[i\right]},\sigma_{y}^{2}\right),\;i=1,\ldots ,N,$$

$$\gamma_{j}∼N\left(0,\sigma_{\gamma }^{2}\right),\;j=1,\ldots ,n_{\text{bin}}$$

$$\delta_{k}∼N\left(0,\sigma_{\delta }^{2}\right),\;k=1,\ldots ,n_{\text{genes}}$$

$$\eta_{l}∼N\left(0,\sigma_{\eta }^{2}\right),\;l=1,\ldots ,n_{\text{batches}}$$

where the total number of datapoints is $N=n_{\text{genes}}\times n_{\text{distances}}\times n_{\text{genotypes}}\times n_{\text{batches}}$, $y_{i}=\log_{2}\left(\text{elongation inde}{\text{x}}_{\text{i}}\right)$ and there are 8 categorical covariates $X_{0},\ldots ,X_{7}$ that correspond to ILF3-enriched genes in WT cells, background genes in WT cells, ILF3-enriched genes in *Actg1*-NSD cells, background genes in *Actg1*-NSD cells, ILF3-enriched genes in WT;Δ*Ilf3* cells background genes in WT;Δ*Ilf3* cells, ILF3-enriched genes in *Actg1*-NSD;Δ*Ilf3* cells and background genes in *Actg1*-NSD;Δ*Ilf3* cells, respectively. Implementing the above model using the lme4 package (v. 1.1–37) (124) in R (v. 4.2.1), the following inferences can be made for the differential impact on elongation index for ILF3-enriched genes in the different genotypes: ILF3-enriched genes showed a significantly higher elongation index in *Actg1*-NSD relative to WT (estimate = 0.43959, SE = 0.0704, Z = 6.244, p = 4.24×10^−10^), no detectable difference between WT and WT;Δ*Ilf3* (estimate = −0.09368, SE = 0.096, Z = −0.976, p = 0.33), and a significantly lower elongation index in *Actg1*-NSD;Δ*Ilf3* relative to *Actg1*-NSD (estimate = −0.57614, SE = 0.0798, Z = −7.216, p = 5.35×10^−13^). Both of the statistical approaches above reach consistent conclusions.

### RNA-seq

RNA from *Actg1* NMD vs *GFP-2A-RFP* NMD transgenic MEFs was isolated using the RNA Clean & Concentrator-5 kit (Zymo) three days post doxycycline induction after extended culture in media with Tet system–approved FBS (thermos) prior to doxycycline induction. To avoid contamination with genomic DNA, samples were treated by on-column DNase digestion. Total RNA and library integrity were verified on LabChip Gx Touch 24 (Perkin Elmer). 10 ng of the total RNA was used as input for the SMARTer^®^ Stranded Total RNA-Seq Kit v2 - Pico Input Mammalian (Clontech). RNA sequencing (RNA-seq) was performed on a NovaSeq S1 instrument (Illumina), resulting in an average of 70 million reads per library, with 150 × 150 bp paired-end setup. The first three nucleotides of Read 2 were trimmed as per the Pico RNA-seq protocol. Reads were aligned against the mouse reference genome GRCm38 (mm10), with gene annotations from Ensembl release 102, using STAR 2.5.3a (125). The numbers of reads that aligned to genes were counted with featureCounts 1.6.0 from the Subread package with the following parameters -*B -C -s 2 -t exon* (126). Only reads mapping at least partially inside exons were admitted, and these reads were aggregated per gene. Reads overlapping with multiple genes or aligning to multiple regions were excluded. Differentially expressed genes were identified using DESeq2 v.1.38.3.

### Trigger RNAs and ASOs transfection

Trigger RNAs were invitro transcribed from a dsDNA, made from annealed oligos, of the sequence GTGAATTGTAATACGACTCACTATAGGGATGNNNNNNNNNNNNNNNNNNTAA using the T7 mMESSAGE mMACHINE Transcription Kit (Thermo), where the Ns represent the trigger RNA sequence. For each experiment, 140 pmol of each trigger RNA were transfected to 200,000 WT MEFs using Nucleofection (Lonza V4XC-3032) according to the manufacturer’s protocol. HEK cells were transfected using either Lipofectamine MessengerMax (Thermo, LMRNA001) or Nucleofection (Lonza, V4XC-2032), with no major differences observed between the two methods in the pilot experiments. Nucleofection was used for most repeat experiments, as it yielded more robust results, especially for the 44-nt *PKD1* trigger RNA, likely due to higher transfection efficiency—an advantage that may be particularly important for low-activity RNAs such as the 44-nt trigger (Fig. 4H). 24 hours post transfection, RNA was extracted from cells for downstream analysis. Filter-binding and electrophoretic mobility shift assay (EMSA) assays provided evidence that the *Actg2* 75-nt trigger RNA binds to ILF3 in vitro. At higher concentrations, the *Actg2* trigger RNA induced several fold stronger upregulations in WT cells, and in some experiments using these higher concentrations, a weaker response was also detected in *Ilf3* KO cells, possibly due to direct interference with antisense RNAs similar to the more chemically stable ASOs or the gRNA-dCas13 control/lacZ experiments. We also tested an uncapped version of the *Actg2* 75-nt trigger RNA (unlike the capped version generated using the mMESSAGE mMACHINE kit used in our experiments), which is expected to be rapidly degraded inside cells. This uncapped RNA, ordered from IDT, did not induce *Actg2* upregulation, supporting that trigger RNA stability and intracellular concentration influence the response. ASOs where designed and ordered through IDT. For *Actg1* trigger regions, we had to expand the windows of design by a few nucleotides 5’ and 3’ of the exact trigger peak region to be able to design at least two ASOs that are predicted to be of good specificity and efficiency. 200 pmol of each ASO was nucleofected into MEFs or HEKs using the SG Cell Line 4D-Nucleofector^®^ X Kit (Lonza, V4XC-3032) or the SF Cell Line 4D-Nucleofector^®^ X Kit (Lonza, V4XC-2032) as per the manufacturer’s protocol. 24 hours post nucleofection, RNA was extracted from cells for downstream analysis. 5’6-FAM conjugated versions of the *Actg2* ASOs were also ordered through IDT, nucleofected to MEFs, and analyzed 24 hours post nucleofection on a NXT Flow Cytometer (Thermo Fisher). ASO and trigger RNA sequences are in table S5.

### Western Blot Analysis

Cells were collected then lysed by rocking them for 15 minutes at at 4°C in RIPA lysis buffer (Thermo) supplemented with 1x Halt Protease and Phosphatase Inhibitor Cocktail (Thermo Fisher, 78446), followed by centrifugation for 5 minutes at 5,000 rcf to obtain clarified cell lysates. Protein concentrations within the lysates were quantified using the Pierce BCA Protein Assay kit (Thermo Fisher, 23225) for equal loading. 4–5 μg of protein were then boiled with 6x Laemmli SDS buffer at 95°C for 5 minutes before proteins were separated on Bolt 4–12% Bis-Tris gels (Thermo Fisher, NW04127BOX) then transferred to Nitrocellulose membranes using the Mini Trans-Blot Cell (BioRad) kit at the high molecular weight settings. Following transfer, membranes were blocked with EveryBlot Blocking Buffer (BioRad, 12010020) at room temperature for 1 hour, and subsequently incubated with primary antibodies, at dilutions indicated by the manufacturer, overnight at 4°C. The following day, membranes were washed 3 times with 1X TBST followed by a 1 hour incubation with the secondary antibodies. 3 washes in 1X TBST were then performed and the membranes were then incubated with 2 ml working solution of SuperSignal West Pico PLUS Chemiluminescent Substrate (Thermo, 34577) and imaged using a ChemiDoc MP system (Biorad). For western blots on nuclear lysates, nuclei were first isolated through a modified version of the 10xGenomics Isolation of Nuclei for Single Cell RNA Sequencing & Tissues for Single Cell RNA Sequencing protocol (CG000124, Rev F) followed by lysis in RIPA buffer. The following antibodies were used: Primary antibodies against ILF3 (abcam, ab92355), ILF2 (Cell signaling technology, 63079), RNASEH1 (Proteintech, 15606–1-AP), Cas13d (abcam, ab314741), YY1 (Cell Signaling Technology, 46395), UPF1 (Cell signaling technology, 12040), ACTG1 (EMD Millipore, MABT824), GAPDH (Cell signaling technology, 2118), VINCULIN (Cell signaling technology, 13901), SRP9 (Proteintech, 11195–1-AP), SERBP1 (Proteintech, 10729–1-AP) and FLAG (Sigma M2 anti-FLAG, M8823). Secondary antibodies used were anti-rabbit IgG, HRP-linked (CST 7074) and anti-mouse IgG, HRP-linked (CST 7076). All blot images presented in this study were screened for potential image duplication or manipulation using ProofIG AI (https://www.proofig.com/); no irregularities were detected.

### Chromatin immunoprecipitation

ChIP was performed using the truChIP Chromatin Shearing Reagent kit (Covaris) using 30 million cells per immunoprecipitation according to the manufacturer’s protocol. Chromatin was sheared using Bioruptor (Diagenode) to generate fragments of 200–1000 bp in size. Immunoprecipitation was then performed as previously described (127). The following antibodies were used: WDR5 (4 μg per immunoprecipitation, 13105, Cell Signaling Technology), H3K4me3 (4 μg per immunoprecipitation, 9751, Cell Signaling Technology), BRG1 (4 μg per immunoprecipitation, 49360, Cell Signaling Technology), YY1 (4 μg per immunoprecipitation, 46395, Cell Signaling Technology), PRMT1(4 μg per immunoprecipitation, ab190892, abcam) and Phospho-Rpb1 CTD (Ser2) (4 μg per immunoprecipitation, 13499, Cell Signaling Technology). Following immunoprecipitation and reverse cross-linking, samples were purified using the NucleoSpin Gel and PCR Clean-up kit (Macherey-Nagel), according to the manufacturer’s protocol for samples containing sodium dodecyl sulfate.

### Co-immunoprecipitation

Cells expressing FLAG-tagged versions of NF110 or LacZ or GFP were lysed in 2ml of buffer containing 50mM HEPES pH 7.5, 200mM NaCl2, 2mM MgAc2, 1 mM DTT, 1% triton x-100 and 1x Halt Protease and Phosphatase Inhibitor Cocktail (Thermo Fisher, 78446). Lysates were rocked for 30 minutes at 4°C, followed by 15 minutes at 37 degrees with or without RNase cocktail (Thermo, AM2286), then spun down for 10 minutes at 10,000 rcf to obtain clarified cell lysate. 50μl of the lysate was set aside and stored at −80 to be used later as input samples. Anti-FLAG Magnetic Agarose Beads (Sigma) (70 μL per sample) were added to lysates and rocked at 4°C for 2 hours. Post-binding, beads were washed 5 times with 1 ml wash buffer and bound material (IP samples) was eluted by resuspending the beads in 100μl of a 150 ng/μl 3XFLAG solution. Eluates were collected by separating the beads with a magnet and collecting the samples. To assess interaction partners, samples were first boiled with 6x Laemmli SDS buffer at 95°C for 5 minutes and proteins were separated on Bolt 4–12% BisTris gels (Thermo Fisher, NW04127BOX), and visualized by immunoblotting as described above. The following primary antibodies were used: UPF1 (Cell signaling technology, 12040), phospho-UPF1(Ser1127) (EMD Millipore, 07–1016), PELO (abcam, ab309344), WDR5 (Cell signaling technology, 13105), GAPDH (Cell signaling technology, 2118), FLAG (Sigma M2 anti-FLAG, M8823).

### Quantifying mRNA half-lives by transcription inhibition

Cells were treated with 10 μg/ml actinomycin D (Sigma) to block transcription and where then collected in TRIzol at various time points post-treatment for RNA extraction. Rn18s was used as a housekeeping gene, as its expression level was not affected over the time course of treatment. Half-lives were then quantified from fitted nonlinear exponential decay curves, and *P* values were calculated using the time points that captured the dynamic phase of decay, excluding the pre-treatment reference point and the final time point, at which transcript levels were uniformly low across the tested genotypes.

### Metabolic labeling pulse-chase experiment

A day prior to the experiment, 2.25 million *Actg1*-NSD or *Actg1*-NSD;Δ*Ilf3* MEFs were seeded in 10 cm dishes (nine dishes per genotype). On the following day, the culture medium was replaced with fresh medium containing 500 μM 4-thiouridine (4sU; Sigma-Aldrich) for 1 hour (pulse period). At the end of the pulse, cells corresponding to the 0-hour time point were washed with ice-cold PBS and lysed directly in 1 mL TRIzol (one dish per replicate). For the chase samples, the medium was replaced with medium containing 5 mM uridine (Sigma-Aldrich) for 1 hour, followed by replacement with medium containing 2 mM uridine for the remainder of the chase period. Seven hours after the end of labeling, cells were washed with ice-cold PBS and lysed in 1 mL TRIzol (two dishes per replicate). Labeled RNA was purified as previously described (128). Briefly, total RNA was extracted using the miRNeasy Mini Kit (Qiagen), and the 4sU-labeled RNA was biotinylated with biotin-HPDP (Thermo Fisher Scientific). Biotinylated RNA was isolated using the μMACS Streptavidin Kit (Miltenyi Biotec) and subsequently used for reverse transcription and qPCR analysis.

### CRISPRn perturb-seq library design and cloning

We designed a dual gRNA CRISPRn library composed of closely spaced gRNAs targeting 147 genes that included 10 negative-control non-expressed genes: *MAGEA5*, *FOLH1B*, *TBC1D3B*, *SPATA31C2*, *ZNF806*, and 5 olfactory receptors (*OR4F29*, *OR1F1*, *OR2C1*, *OR3A1* and *OR3A2*), in addition to 5 pairs of non-targeting control sgRNAs. The genes targeted spanned a wide range of gene ontology terms that included subsets of: (i) orthologs of genes targeted in previous genetic compensation studies (13, 14) (ii) genes identified to have stronger growth effects when targeted by CRISPRn versus CRISPRi and vice versa as identified in screens performed in a previous study (129) (iii) Cancer Dependency Map common essential genes as defined in 20Q1 (iv) non-essential genes (v) genes that are under the control of bi-directional promoters (vi) 10 negative-control non-expressed genes as CRISPRn double-stranded breaks control (vii) non-targeting control sgRNAs; the library was designed to include 9–10% control gRNAs (negative control and non-tageting gRNAs). To increase the potential of having an out-of-frame mutation that will elicit NMD, and to be on-par with the CRISPRi Perturb-seq dataset to which the data was going to be compared (52), a multiplexed CRISPRn library was constructed which targeted each gene with two unique sgRNAs expressed from tandem U6 expression cassettes in a single lentiviral vector (105). The Human Improved Genome-wide Knockout CRISPR Library (130) and the Brunello library (107) CRISPRn sgRNA library were used as a source of sgRNAs targeting each gene, with the optimal sgRNA pair targeting each gene selected to be the closest two sgRNAs to each other to avoid having large deletions that can influence TA responses. We also avoided sgRNAs targeting within the first 150 nucleotides of an open reading frame as stop codons in these regions can escape nonsense-mediated decay (131). The sgRNAs used can be found in table S6. Cloning of the dual gRNA libraries with capture sequences for 3’ direct capture Perturb-seq into an sgRNA lentiviral expression vector (pJR101, Addgene #187241) was performed as described before (52, 105, 132) and here: https://weissman.wi.mit.edu/resources/2022_crispri_protocols/Protocol_1_dual_sgRNA_lib_cloning.pdf. Briefly, a two-step restriction enzyme digestion and ligation cloning of oligos into pJR101 was performed to maintain coupling of sgRNAs targeting the same gene. Oligos encoding the targeting regions of dual-sgRNA pairs were synthesized as an oligonucleotide pool (Twist Biosciences) with the structure: 5’- PCR adapter - CCACCTTGTTG – targeting region A - gtttcagagcgagacgtgcctgcaggatacgtctcagaaacatg – targeting region B - GTTTAAGAGCTAAGCTG - PCR adapter-3’. Oligo pools were amplified, digested with BstXI/BlpI, and ligated into pJR101. To add an sgRNA constant region and hU6 promoter to the vector, pJR89 (Addgene #140096) was BsmBI-digested and and the sequence encoding the sgRNA constant region and hU6 promoter was ligated into the intermediate library.

### Perturb-seq

CRISPRn perturb-seq experiments were performed similar to the day 8 genome-wide CRISPRi perturb-seq (52) to allow for direct comparison of the two different datasets. The CRISPRn library was packaged into lentivirus in 293T/17 cells and K562 Cas9 cells were transduced via spinfection (1000g) with polybrene (8 μg/ml) with the target of obtaining an infection rate of ∼30%. Cells were maintained at a viability of >90%, a coverage of 1000 cells per library element, and a density of 250,000 to 1,000,000 cells/ml for the course of the experiment. Three days post transduction, cells were sorted to near purity by FACS (FACSAria2, BD Biosciences), using GFP as a marker for sgRNA vector transduction. Eight days post infection, the cells were measured to be 97% GFP+ (LSR2, BD Biosciences), >90% viable, and at a concentration of ∼800,000 cells/ml (Countess II, ThermoFisher). Cells were prepared for single-cell RNA-sequencing by resuspension in 1X PBS with 0.04% BSA as detailed in the 10x Genomics Single Cell Protocols Cell Preparation Guide (10x Genomics, CG00053 Rev C). Cells were then separated into droplet emulsions using the Chromium Controller (10x Genomics) with Chromium Single-Cell 3′ Gel Beads v3 (10x Genomics, PN-1000075) across 3 “lanes”/”GEM groups” following the 10x Genomics Chromium Single Cell 3ʹ Reagent Kits v3 User Guide with Feature Barcode technology for CRISPR Screening (CG000184 Rev C) with the goal of recovering ∼20,000 cells per GEM group before filtering. To perform the CRISPRn perturb-seq experiment in *ILF3*-knockout K562 cells, K562-Cas9 cells were co-transduced via spinfection (as described above) with two components: (1) an all-in-one Cas12 expression vector co-expressing four sgRNAs targeting ILF3, and (2) the CRISPRn perturb-seq library. The spinfection was optimized to achieve an infection rate of ~30% for each independently. Three days post-transduction, double-positive cells were isolated by FACS (FACSAria2, BD Biosciences), using mCherry as a marker for the Cas12-sgRNA vector and GFP to mark the CRISPRn perturb-seq library, enriching for cells expressing both constructs to near purity. Eight days post infection, the cells were measured to be 97% double positive for GFP and mCherry (LSR2, BD Biosciences). Cells were prepared for single-cell RNA-sequencing by resuspension in 1X PBS with 0.04% BSA as detailed in the 10x Genomics Single Cell Protocols Cell Preparation Guide (10x Genomics, CG00053 Rev C). Cells were then separated into droplet emulsions using the Chromium Controller (10x Genomics) with Chromium Single Cell 3’ HT Gel Bead Kit v3.1 (10x Genomics, PN-1000370) across 2 “lanes”/”GEM groups” following the Chromium Next GEM Single Cell 3’ HT Reagent Kits v3.1 User Guide with Feature Barcode technology for CRISPR Screening (CG000421Rev D) with the goal of recovering ∼30,000 cells per GEM group before filtering. As noted in the main text, while loss of ILF3 did not seem to affect cell proliferation in MEFs, it led to an observable cell proliferation phenotype in K562s which is consistent with its reported essentiality in K562s in the cancer dependency map portal (DepMap). While we performed the experiment at a time point preceding decreased cellular proliferation, it is nevertheless important to note that while our analyses provide a pool of TA-candidate pairs which can be used to globally explore requirements for TA and ILF3 involvement, as with any large-scale study, individual gene pairs need to be validated before undertaking focused functional investigations. For the *ILF3* KO Perturb-seq experiment, WT cells transduced with four different control gRNAs were pooled with *ILF3* KO cells prior to GEM generation in proportions ensuring representation equivalent to other gRNAs. This allowed inclusion of a WT control population alongside the *ILF3* KO cells transduced with control gRNAs. The analyses presented in the manuscript use *ILF3* KO cells transduced with control gRNAs as the control group; however, conclusions were unchanged when using WT cells as controls. For preparation of gene expression and sgRNA libraries, samples were processed according to 10x Genomics Chromium Single Cell 3ʹ Reagent Kits v3 or v3.1 User Guide with Feature Barcode technology for CRISPR Screening (CG000184 Rev C, CG000418 Rev D). For sequencing, mRNA and sgRNA libraries were pooled to avoid index collisions at a 10:1 ratio. Libraries were sequenced on a NovaSeq (Illumina) according to the 10x Genomics User Guide. Following sequencing, reads were used as input to Cell Ranger for alignment. In total, 55846 cells were sequenced for the perturb-seq experiment in WT K562 Cas9 cells and 63554 for the *ILF3* knockout cells.

#### Alignment, cell calling, and guide assignment

Cell Ranger 6.1.2 software (10x Genomics) was used for alignment of scRNA-seq reads to the transcriptome, alignment of sgRNA reads to the library, collapsing reads to UMI counts, and cell calling. The 10x Genomics GRCh38 version 2020-A genome build was used as a reference transcriptome. Reads from the sgRNA libraries were mapped with Cell Ranger. To account for differences in sequencing depths across GEM groups from the same experiment, reads were downsampled to produce a more even distribution of the number of reads per cell across gemgroups, with a threshold of 1000 reads per cell. Guide calling was performed with a Poisson-Gaussian mixture model as previously described. For each guide, the mixture model was fit 100 times, selecting the maximum likelihood model from among the fits. After guide calling, each cell was categorized according to its guide identities as representing a single genetic perturbation or a multiplet (which may arise from lentiviral recombination or multiple cell encapsulation during droplet generation). Only cells bearing two guides targeting the same gene were used for downstream analysis. Downstream analyses were performed in Python, using a combination of numpy, scipy, Pandas, scikit-learn, pomegranate, infercnvpy, pygenometracks, scanpy and seaborn libraries as described before (49, 52).

#### Normalization of gene expression measurements and gene-level differential expression testing using the Mann-Whitney tests

We used normalization processes similar to the one used for the CRISPRi genome-wide perturb-seq (52) and as described before (49) using control non-targeting sgRNAs. We then computed a normalized gene expression matrix for cells via UMI count normalization where we scaled expression within all cells so that their total UMI counts equal the median UMI count of core control cells within the experiment). We then test for each gene whether the distribution of normalized expression is identical between control cells bearing non-targeting sgRNAs and cells bearing each perturbation. Only genes detected in at least 3 cells were analyzed, and only cells where at least 200 genes were detected were kept for the analysis. We used the Mann-Whitney U test (scipy.stats.mannwhitneyu) implemented in scipy, which tests whether one distribution is stochastically greater than another. We used the asymptotic *P* values and excluded any perturbation with fewer than 40 cells. *P* values were adjusted for multiple hypothesis testing using three complementary correction strategies: (i) Bonferroni correction across the 84 perturbed genes (significance threshold: P ≤ 0.05/84 = 5.95E-4), (ii) Benjamini-Hochberg (BH) correction applied globally across all gene-pair comparisons (n = 692,664; significance threshold: adjusted *P* ≤ 0.05), and (iii) BH correction applied locally within each perturbation-specific gene-pair set (n = 8,246 per perturbation; adjusted *P* ≤ 0.05). A gene-pair was considered significantly affected by a perturbation if it passed the adjusted significance threshold under any of the three correction strategies. This inclusive approach was chosen to maximize detection sensitivity across a range of multiple-testing correction stringencies, balancing Bonferroni with the broader detection power of BH procedures. However, as noted in the main text, while the resulting set of TA-candidate and control gene pairs provides a valuable resource for globally exploring the requirements of TA, individual gene-pair relationships should be treated as candidates, as global patterns can be highly informative but individual outcomes may be influenced by confounding factors that may include gRNA off-target, batch effects or Cas9-specific double-strand breaks. These relationships should therefore be subjected to orthogonal or functional validation prior to focused investigation.

#### Data analysis

While the library targeted 147 genes, the downstream analyses focused on 84 genes only. Besides perturbations that were eliminated in the quality control steps described above, we excluded genes that were either missing in the CRISPRi dataset or whose levels following CRISPRi-mediated knockdown was not < 0.33. *TUBA1C* was also excluded as we observed that one of the designed gRNAs had a perfect match with another gene *TUBA1B*. For downstream analysis we examine “gene pairs” in which we analyze the expression levels of genes (hereafter referred to as observed genes) upon perturbing a given gene (hereafter referred to as perturbed gene). TA-candidate gene-pairs were identified as those where the observed gene was significantly (equivalent to a corrected *P* value of <= 0.05) upregulated upon CRISPRn-mediated perturbation of the perturbed gene by a fold change >= 1.5 and that were either: a) not significantly upregulated upon CRISPRi-mediated perturbation of the same perturbed gene or, b) if it is, the fold change in upregulation of the observed gene upon CRISPRn-mediate perturbation must be at least 1.5 times higher than what is observed with CRISPRi. Control gene pairs where identified as those with the opposing criteria (i.e., the observed gene is significantly upregulated upon CRISPRi-mediated perturbation of the perturbed gene by a fold change >= 1.5 and that was either: a) not significantly upregulated upon CRISPRn-mediated perturbation of the same perturbed gene or, b) if it is, the fold change in upregulation of the observed gene upon CRISPRi-mediate perturbation must be at least 1.5 times higher than what is observed with CRISPRn). The rationale for the choice of those control pairs was to define a comparison group in which the observed (assessed) gene was amenable to upregulation, but in a TA-independent manner. This approach allowed us to avoid including genes located in compact heterochromatin environments that are generally not amenable to upregulation. In addition, it enabled comparison to a group of similar size, rather than to all possible gene pairs (>650K), which would have required computationally intensive subsampling analyses across all analyses. While our analysis identified only one paralogous pair among the TA-candidate pairs, this likely reflects the relatively lower sensitivity of scRNA-seq in detecting small expression changes, as well as the relatively strict thresholds used to define TA candidates. For example, applying a very relaxed criterion of CRISPRn fold change > 1 and CRISPRn/CRISPRi ratio > 1 yields 156 of the 439 possible paralogous pairs. Although most of these are very unlikely to represent TA candidates, a few may correspond to genuine but weak TA responses that could be of interest for future validation studies. We nonetheless chose to retain stricter thresholds to maintain higher confidence in the candidates included in our global analysis.

##### Assessment of similarity of perturbation transcriptional profiles in the different experiments

CRISPRn and CRISPRi perturbation of a given gene similarly led to transcriptional responses that are a signature of successful perturbations. For each perturbed gene the total number of differentially expressed genes (DEGs) was close for the two methods of perturbation (fig. S2A). As apparent in the figure, a few outliers existed—for example, *RPL26*, which had 900 control gene pairs—but we chose to retain them in the analyses, as they may reflect true biological effects. As a test, we excluded some outliers from some of our analyses and found that their removal did not affect the conclusions. We applied UMAP to normalized transcriptomic profiles with parameters n_neighbors =2, min_dist=0 and random_state=42 to generate two- dimensional (2D) embeddings for each perturbed gene in either Perturb- seq experiments. For each perturbed gene, Euclidean distance in high-dimensional space between the two embeddings were calculated as an imperfect proxy for how similar the transcriptome-wide response between CRISPRn Perturb- seq and CRISPRi Perturb- seq experiments were. However, to assess the similarity of transcriptomic responses between CRISPRn and CRISPRi perturbations (fig. S2B), we first applied principal component analysis (PCA) to transform the transcriptomes into an orthogonal, lower-dimensional space, using varying numbers of components. We then calculated Euclidean distances between transcriptomic profiles in this PCA-transformed space. This approach reduces noise and highlights major sources of variance, which is particularly helpful when comparing self-perturbations (same gene) versus non-self perturbations (different genes), where signal can be diluted by background transcriptional variability. As a background comparison, for each gene perturbed by CRISPRn, we calculated the median distance between its CRISPRn-induced profile and the CRISPRi-induced profiles of all other (nonself) genes. We found that CRISPRn and CRISPRi perturbations of the same gene were significantly closer in PCA space than CRISPRn perturbations of a gene compared to CRISPRi perturbations of other genes (fig. S2B). A similar pattern was observed when comparing CRISPRn perturbations in wild-type versus *ILF3* knockout cells (fig. S4C), confirming that perturbations of the same gene cluster more closely than those of different genes—indicating similarly successful perturbation and broadly similar global transcriptomic responses across conditions. For other analyses, we used Euclidean distances in high-dimensional gene expression space (fig. S2C, D) to capture the full complexity of the transcriptomic profiles without relying on dimensionality reduction.

#### Annotation of gene elements

Unless noted otherwise, we used the genetic coordinate information of each gene and its canonical transcript found in Ensembl v109, hg38. The region ± 2500 base pair around the transcription start site was annotated as promoter. As there are multiple ways to define enhancers and connect enhancers to genes, we used annotations from 4 diverse datasets to be comprehensive. One of the main ways to define enhancers are from epigenetic marks. *Enhancer_epimap* are 239,349 candidate-enhancer regions from the Epimap dataset (https://compbio.mit.edu/epimap/, (133)). These candidate enhancer elements are defined by the 18-state ChromHMM Roadmap model from observed and imputed tracks of six histone marks (H3K27ac, H3K4me1, H3K4me3, H3K36me3, H3K9me3, H3K27me3) in K562 sample BSS00762. Enhancers were connected to genes using minimum 0.7 correlation threshold between epigenetic marks and gene expression (links_corr_only), as recommended by the authors. *Enhancer_ABC* are candidate-enhancer regions defined by epigenetic marks for the sample “K562-Roadmap” in (134). Enhancers were connected to gene using prediction from ABC method, which predict enhancer–gene connections based on measurements of chromatin accessibility (ATAC-seq or DNase-seq), histone modifications (H3K27ac ChIP–seq), and chromatin conformation (Hi-C). Enhancer-gene pairs with ABC score > 0.015 were used for further analyses, which resulted in 61,981 regions. *Enhancer_eRNA_Yulab* and *Enhancer_eRNA_Lidschreiber* are both putative enhancer regions with evidence of transcription of relatively short-lived, divergent enhancer RNA transcripts. *Enhancer_eRNA_Yulab* are 70,107 proximal and distal elements defined by integrating data from 7 RNA-seq assay methods to detect eRNA in K562 (https://pints.yulab.org/, (135)) and linked to the nearest gene. *Enhancer_eRNA_Lidschreiber* are 12,854 putative enhancer elements that show evidence of intergenic and antisense RNA transcription, identified via transient transcriptome sequencing (136). The authors provided 3 methods to connect enhancer to gene (PairedNearest, PairedCorrelatedNeighbouring and PairedCorrelatedWindow), so we used gene-enhancer pairs linked by at least one method, to be inclusive.

#### Sequence similarity analysis

We performed sequence similarity analyses between the perturbed gene’s cDNA sequence and the aforementioned observed gene’s elements using BLASTn (137). cDNA sequence of the perturbed gene’s canonical transcript was obtained from Ensembl v109, along with coordinates of exons, cDNA coding region, and UTRs. Only the observed genes that appeared in TA-candidate and the control gene pairs were included in the BLAST analysis. Genetic coordinates for the observed genes’ elements were obtained from the various datasets as mentioned, converted to hg38 coordinates using liftOver if needed, and used to retrieve nucleotide sequence. BLASTn analysis was performed comparing each perturbed gene’s cDNA sequence against each of the 6 sequence databases of observed gene’s elements (gene body, promoter, enhancer_epimap, enhancer_ABC, enhancer_eRNA_Yulab, enhancer_eRNA_Lidschreiber) with parameters word size 4 and E value up to 100000 to capture all possible alignments. Same BLASTn criteria was used for the analysis in fig. S7E. We note that the conclusions presented in fig. S3A (and 1J) did not change when non-significantly changed genes (based on corrected P values) were assigned a fold-change value of 1.

#### ILF3 motif enrichment analysis

Eight motifs for ILF3 in K562 were in mCrossBase, a database of RNA-binding protein binding motifs and crosslink sites defined jointly from ENCODE’s eCLIP data (https://zhanglab.c2b2.columbia.edu/mCrossBase/index.php, (53, 54)). We used MAST from MEME Suite version 5.5.3 to search sequences of BLASTn alignments between gene pairs for matches to the set of ILF3 motifs. MAST was specified to score only the exact alignment sequence and not the reverse complement, with an Evalue threshold < 1000, and used as background a random sequence model that assumes each position in a random sequence is generated according to the average letter frequencies in the database of all BLASTn alignment sequences. For each sequence, MAST returns a position p-value for each identified motifs, sequence p-value, and sequence E value. High-confidence matches are identified as motif matches with a p-value < 0.0001. The sequence p-value is the combined best matches of a sequence to the group of ILF3 motifs, and sequence E value is the probability of observing a sequence p-value at least as small in a random sequence file of the same size.

#### Similarity of genes in Exome-wide association study’s significance patterns

Gene-level burden test summary statistics from a recent exome-wide association study (138) was downloaded. The summary statistics was stratified by phenotype, variant consequence (pLOF, DelMissense, pLOF_and_DelMissense) and variant MAF (singleton, <0.001%, < 0.01%, < 0.1%, < 1%). We included genes from a) TA candidate gene pairs, b) control gene pairs. We obtained for each gene a p-value for each combination of [gene]_ [variant_consequence]_[MAF]_[phenotype] (for example, [ACTG1]_[pLOF]_[<0.001%]_[Coffee_consumed]). This resulted in a matrix of shape (2870, 39,850). PCA is applied to this matrix to reduce it to 2,700 principal components, resulting in a matrix of shape (2870, 2700). The number of components was chosen based on the finding that 2,131 components explain 95% of the variance. Thus, each perturbed gene can be represented by 2,700 numbers. Euclidean distances between gene pairs were calculated from different numbers of consecutive components. We experimented with different combinations of components and observed similar results.

## Supplementary Material

Table S1

Table S2

Table S3

Table S4

Table S5

Table S6

Supplementary Materials

Supplementary Materials

Supplementary Text

Figs. S1 to S12

Tables S1 to S6

References (142–179)

## Data Availability

Data are available either in the main text or the supplementary materials. Raw sequencing files were deposited to the Gene Expression Omnibus GEO under accession code GSE256101. Cell lines and plasmids used in the study are available for sharing once requested.
