# Supplementary material for: Mechanisms linking cytoplasmic decay of translation-defective mRNA to transcriptional adaptation
Source: Science. Author manuscript; Available in PMC 2026 Jun 22. (PMC13286266; doi:10.1126/science.aea1272)
Supplement: Supplementary Materials [file NIHMS2149358-supplement-Supplementary_Materials.docx]

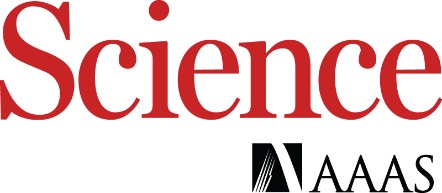


Supplementary Materials for

**Mechanisms linking cytoplasmic decay of translation-defective mRNA to transcriptional adaptation**

Mohamed A. El-Brolosy^1,2,3,4*^, Atharv Oak^2,5^†‡, An T. Hoang^2^†, Yassine Damergi^2^†§, André Fischer^2^†¶, Reuben A. Saunders^1,2^, Jingchuan Luo^2^, Amer Balabaki^2,5^, Jeremy Guez^3,4^, Troy W. Whitfield^2^, Seth R. Goldman^6^, Arash Latifkar^2^, Yuancheng Ryan Lu^2^, Didier Y.R. Stainier^7^, Konrad J. Karczewski^3,4,8^, Olivia Corradin^2,5^, Jonathan S. Weissman^2,5,9,10*^

Corresponding author: mohamed_elbrolosy@fas.harvard.edu (M.A.E.-B.); weissman@wi.mit.edu (J.S.W.)

**The PDF file includes:**

Supplementary Text

Figs. S1 to S12

**Other Supplementary Materials for this manuscript include the following:**

Tables S1 to S6

**Supplementary Text**

***Quality control analyses of TA-candidate responses identified from the perturb-seq analyses***

As a quality control, we verified that for each perturbed gene, the total number of differentially expressed genes (DEGs) was comparable between CRISPRn and CRISPRi perturbations (fig. S2A). Transcriptomic responses to CRISPRn and CRISPRi perturbation of the same gene were significantly more similar than responses to perturbations of different genes (fig. S2B), suggesting consistent gene-specific effects across both approaches. Moreover, the degree of similarity between CRISPRn and CRISPRi transcriptomic profiles for a given gene was not correlated with knockdown efficiency or NMD levels, but rather with the number of TA-candidate and control observed genes (fig. S2C, D), suggesting that differences in transcriptomic responses are not due to differences in perturbation strength. The number of TA-candidate observed genes for a given perturbation was not correlated with CRISPRi knockdown efficiency and it was only moderately correlated with the gene’s baseline expression in wild-type cells or gene essentiality as reported by DepMap (fig. S2E, F). Altogether, these data suggest that, in addition to shared global effects, transcriptional changes specific to CRISPRn are not driven by several potential confounding factors, and represent candidate TA responses.

Sequence similarity between the mutant gene’s mRNA and the adapting gene’s gene body or regulatory regions is a hallmark of TA (*13, 14, 67*). In a first-pass analysis, we examined genes that were significantly upregulated upon CRISPRn perturbation and showed stronger upregulation by CRISPRn than by CRISPRi (defined as fold change >1 for CRISPRn and a CRISPRn/CRISPRi fold change ratio >1). Genes exhibiting detectable sequence similarity to the perturbed gene’s mRNA, using a permissive E-value threshold of <1000, displayed significantly greater upregulation by CRISPRn relative to CRISPRi, compared to the full set of upregulated genes (fig. S3A). Notably, the more stringently defined TA-candidate gene pairs (fold change ≥1.5 for CRISPRn and CRISPRn/CRISPRi fold change ratio ≥1.5) exhibited higher levels of sequence similarity relative to multiple control groups tested (fig. S3B). These results indicate that our comparative Perturb-seq approach can identify candidate TA responses. For subsequent analyses, we used the control pairs as the non-TA comparison group, as they represent a group in which the observed (assessed) gene is amenable to upregulation upon gene perturbation, but in a TA-independent manner. This approach also allowed us to use transcriptionally responsive genes as controls while avoiding those that are generally refractory to expression changes under cellular perturbation. In addition, it enabled comparison to a group of similar size, rather than to all possible gene pairs (>650K), which would have required computationally intensive subsampling across all analyses.

Next, using a UK Biobank exome-wide association study (*138*), we found that TA-candidate gene pairs—each comprising a perturbed gene and its corresponding adapting observed gene—showed greater similarity in their gene-level association patterns with health-related traits, based on the burden of rare coding variants, than the control gene pairs (fig. S3C). These findings suggest that, within the context of TA, adapting genes are more likely to influence similar biological functions or pathways as the perturbed genes. The rich dataset also allowed us to identify additional features associated with TA. First, we found a preference for longer stretches of sequence similarity between the perturbed gene and the adapting observed gene. TA-candidate gene pairs with high-confidence sequence similarity (E value <1; −log_10_ E-value > 0) displayed longer normalized alignments—relative to the length of the aligned-to region in the observed gene—compared to control pairs (fig. S3D). Second, sequence similarity to regulatory regions of the adapting gene also appeared to contribute to TA (fig. S3A, D, E). Within enhancers, TA-candidate pairs with high-confidence sequence similarity displayed longer and higher-scoring alignments than control pairs, typically when evidence of enhancer RNA (eRNA) transcription was present (fig. S3D, E). Finally, we found no evidence of positional bias in the location of sequence similarity relative to the adapting gene body; whether the similarity was located toward the 5′ or 3′ end did not appear to influence TA (fig. S3F).

As a quality control for the CRISPRn Perturb-seq experiment in *ILF3* KO cells, we found that the total number of differentially expressed genes (DEGs) per perturbation was correlated between CRISPRn Perturb-seq experiments in WT and *ILF3*KO cells (fig. S4B). Additionally, CRISPRn perturbations of the same gene in WT and *ILF3*KO cells produced more similar transcriptomic profiles than perturbations of different genes (fig. S4C).

While our ILF3 knockout (KO) CRISPRn Perturb-seq experiment was performed at day 8 post-transduction, prior to the major proliferation defects observed with prolonged ILF3 loss in K562 cells (fig. S4D), we nevertheless sought to test whether proliferation defects could influence TA. To this end, we examined three different proliferation-limiting conditions: (a) growth in 2% FBS, which decreases cell proliferation to an extent similar to that observed with prolonged ILF3 loss (fig. S4D), and (b) knockout of two randomly selected genes with reported RNA-binding activity (*139, 140*) and comparable proliferation-limiting effects to ILF3 based on DepMap (*116*) gene effect sizes in K562 cells (ILF3: –1.060), namely SRP9 (–0.968) and SERBP1 (–1.136) (fig. S6A, B). Unlike the loss of ILF3 (Fig. 1M), CRISPRn-mediated perturbation of *DDX21* under these ILF3-independent proliferation-limiting conditions still led to upregulation of the adapting genes (fig. S6C). We also confirmed that these conditions did not affect adapting gene expression when *DDX21* was not targeted (fig. S6D), and that they did not lead to increased levels of the mutant mRNA (fig. S6E).

***Supplementary Discussion***

ILF3 is a versatile RBP that is implicated in several processes including splicing (*47*), other gene expression regulation processes (*34-37*), and functions unrelated to gene expression (*45, 46, 142*). This functional breadth may help explain the perinatal lethality of *Ilf3* knockout mice (*143*), as well as the reduced proliferation observed in K562 and HEK cells. Here, we expand ILF3’s role to include transcriptional adaptation (TA). Our screen also identified nuclear RNA decay factors as ZC3H18 as regulators of *Actg2* levels in *Actg1*-NSD cells (fig. S1C), suggesting that TA may be influenced by nuclear RNA decay, either directly as supported by recent studies (*23, 26*) or via the degradation of repressive antisense transcripts. Further investigation will be needed to clarify the underlying mechanisms.

Through high-throughput CRISPRn *vs* CRISPRi perturb-seq experiments, we show that TA is a widespread response that is broadly dependent on ILF3. The rich Perturb-seq dataset allowed us to define key factors influencing TA, including similarities within regulatory regions as drivers of TA that may rely on hybridization with regulatory RNAs such as enhancer RNAs. Additional comparative studies across diverse cell types, coupled with machine learning approaches, may help uncover further determinants of TA responses (*144, 145*). For example, a recent study suggested that TA responses can vary between different cell lines (*23*). Such variability may be shaped by multiple factors that comparative approaches can help disentangle, including the expression levels of target RNAs such as the presence or absence of antisense transcripts at sites of sequence homology (particularly in cases involving paralogous genes or self-TA) and how they may be modulating the expression of their sense counterparts. Additional influences may include the chromatin landscape at adapting gene loci, the expression level of the perturbed gene—which could affect the levels of TA-inducing decay fragments—and secondary effects that arise from the loss of the encoded protein. Cell-type-specific differences in ILF3 expression or isoform usage, as well as its essentiality—as we observed in K562 and HEK293 cells but not in MEFs—may also play a role. Other potential modulators may include dependencies on other RBPs or chromatin modifiers (e.g., the RNAi machinery in *C. elegans* (*16*), or UPF3A (*14, 21*)) and variation in mRNA decay kinetics. Within a given cell line, these factors will also influence whether a gene exhibiting sequence homology to a mutant mRNA will be upregulated or not. Consequently, they may also shape how many adapting genes are associated with a given perturbed transcript, and their levels of upregulation. Moreover, the number of adapting genes within TA-candidate gene pairs varied across different perturbed genes (fig. S2D–F). In addition to the factors mentioned above, it remains unclear whether this variability—discussed further below—reflects intrinsic properties of the mutant mRNAs, their degradation intermediates, or the nature of the targeted RNAs. Some TA responses—particularly those lacking extensive sequence similarity—may reflect indirect secondary effects. Alternatively, they could be driven by sequence similarity to regulatory elements, such as distal enhancers whose connections to the adapting gene have not yet been established, or by very minimal homology below the threshold of detection that is nonetheless sufficient to mediate TA.

We observed that in some cases, RNASEH1 overexpression led to enhanced upregulation of the adapting genes (fig. S7A). One possible explanation is that RNASEH1 resolves R-loops that normally impede transcription, thereby allowing further transcriptional activation of these genes. R-loops can act as transcriptional roadblocks by interfering with RNA polymerase II elongation (*146-150*); thus, their removal may potentiate the transcriptional elongation effects associated with TA. Other, more complex mechanisms may also contribute and warrant future investigation.

Future studies will be important to clarify how TA operates when sequence homology is not linked to an antisense RNA. For instance, if decay fragments hybridize to the sense pre-mRNA, this may directly enhance transcription of that gene, as suggested by our data showing increased expression when dCas13-NF110 is targeted to sense pre-mRNAs (fig. S9E, F). TA-like effects may possibly arise from hybridization of decay intermediates to other transcripts at a locus, including enhancer RNAs (eRNAs (*151*)) or promoter associated noncoding RNAs (*152-155*). Such regulatory RNAs could simply act as targets that recruit ILF3 into proximity with gene bodies. More complex mechanisms are also possible—for example, TA could enhance the expression of these regulatory eRNAs or promoter-associated RNAs. eRNAs themselves can amplify transcription through mechanisms similar to those described in this study, including recruitment of the SWI/SNF complex (*156*), maintenance of H3K4me3 levels (*157*), and enhancement of transcription elongation (*158*), among others (*159*). Promoter-associated RNAs may reduce promoter methylation through inhibiting DNMT1 (*154*), or directly activate transcription (*155*). Alternatively, for promoter-associated RNAs overlapping the gene body, TA may involve inhibiting their repressive effects on sense pre-mRNA expression (*153*). It is also possible that secondary effects, whether from protein loss or from changes in the protein levels of the initial TA targets themselves, could give rise to new TA targets. For example, such secondary effects could lead to increased expression of eRNAs, antisense RNAs, or other noncoding transcripts that may then be targeted by decay intermediates (provided sequence homology exists), thereby triggering further TA.

We demonstrate that trigger screens can be used to identify sequences that induce both paralogous and self-TA, including for disease-relevant genes such as *PKD1*. Investigating the prevalence of self-TA will be an important direction for future studies, particularly in the context of heterozygous mutations, where upregulation of the wild-type allele may offer more reliable functional compensation than paralog activation. Supporting the potential for self-TA, a previous study showed that genes harboring PTCs display elevated pre-mRNA levels (*92*), which may reflect transcriptional upregulation at the mutant locus, and PTCs have also been associated with other regulatory phenomena such as nonsense-mediated altered splicing (*160, 161*). Transcriptional silencing following double-strand breaks, however, has been reported in cultured cell systems (*162-164*) and could possibly persist after repair in some cases if epigenetic modifications are inherited. This could obscure detection of self-TA in homozygous mutant cells. Indeed, in our study, we observed no increase in mutant *Actg1* transcription in our PRO-seq dataset from *Actg1*-NSD cells, despite increased ILF3 enrichment (Fig. 2B, D). It is possible in that case that the increased ILF3 recruitment may counteract double-strand break–induced transcriptional silencing. Supporting this possibility, loss of ILF3 in *Actg1*-NSD;Δ*Ilf3* cells led to reduced *Actg1* transcription relative to *Actg1*-NSD cells that have wild-type ILF3 (Fig. 2D). It is however also possible in that case that the increased ILF3 enrichment may reflect ILF3 binding to decay intermediates originating from the *Actg1*-NSD mRNA, and future studies will be needed to further disentangle those possibilities. Overall, self-TA may be more readily detectable in heterozygous settings, as previously reported (*13, 14*). Supporting this, methods that induce TA in wild-type cells, such as the NMD vector used here (Fig. 3A) or Cas13d-mediated perturbations (*26*), have been shown to trigger self-TA and upregulate endogenous *Actg1*. Independently, there may be additional reasons why some genes with increased ILF3 recruitment might not be transcriptionally upregulated in homozygous knockout cells. These can include secondary effects in the knockout context that differentially impact specific genes and potentially counteract ILF3 activity.

Future studies will be important for understanding whether self-TA may influence the penetrance of haploinsufficiency disorders caused by protein-truncating variants, among them polycystic kidney disease (*165*). It will also be important to investigate whether inter-individual genetic or epigenetic differences influence the likelihood or effectiveness of self-TA. Likewise, in genes associated with complete penetrance, it will be informative to understand why self-TA does not occur—whether due to the absence of antisense RNAs that could serve as targets, low NMD efficiency, or other epigenetic features. Alternatively, self-TA may still occur in such cases, but the magnitude of upregulation may be insufficient to promote functional compensation.

Trigger RNAs or ASOs targeting antisense RNAs at regions identified by trigger screens may offer delivery advantages over full-length mRNAs or gene therapy approaches. The physiologically-relevant upregulations observed with TA and trigger RNAs (1.5- to 3-fold (*13, 14*)) may be beneficial for diseases (e.g., diseases of haploinsufficiency) where very high levels obtained with other gene augmentation approaches is not desirable (*166-170*). For example, overexpression of PKD1 also leads to polycystic kidney disease (*166*), suggesting that balanced PKD1 levels are critical, and that therapeutic strategies should aim to restore PKD1 expression to physiological levels (*94*). Moreover, TA has been shown to be trans-generationally inherited (TGTA; (*66*)), suggesting that upregulation responses induced by leveraging the TA machinery may lead to more stable responses that require less frequent dosing. Of note, the incomplete reduction in elongation indices of the RIPseq-identified ILF3-enriched genes upon ILF3 loss in *Actg1*-NSD cells (Fig. 2I) may be explained by TGTA, which involves transgenerational inheritance of TA-associated H3K4me3 marks (*66*). Furthermore, our ASO data were consistent with previous studies that suggested that knocking down antisense transcripts can promote the expression of the sense RNA (*71, 171-174*). Since most antisense transcripts are unannotated and can span large portions of the gene body (Fig. 2A), it is often challenging to determine where ASOs should be targeted to activate sense RNA expression. Trigger screens could help narrow down effective regions for ASO targeting. It will also be interesting to explore whether transcriptional adaptation may contribute to the regulation of genes in which ASO-mediated knockdown of antisense transcripts was previously reported to enhance sense RNA expression (*71, 171-174*).

Our current analysis of trigger screens, based on comparing sequences enriched in the top 10% versus bottom 10% of expressing cells, identifies RNAs that most effectively activate a given target. However, additional “weaker” trigger RNAs may potentially exist outside these peak-enriched regions. Supporting this idea, the *Actg1* TR1 51-nt RNA, which shares sequence homology with exon 9 of Actg2, modestly upregulated Actg2, albeit to a lower degree than the 75-nt RNA identified in the Actg2 trigger screen (fig. S11S). Notably, however, for Actg1 self-TA, where we identified three distinct trigger regions (Fig. 4B), RNAs from each region activated endogenous Actg1 to comparable levels (Fig. 4C). The efficacy of a given trigger RNA may be influenced by multiple factors beyond sequence homology, including RNA structure, or structure stability, the presence of ILF3-recognized sequence or structural motifs, accessibility of the target site (e.g., regions not masked by other RNA-binding proteins), sequence composition and GC content, potential immunogenicity, the number of off-target sites, or the presence of antisense RNAs, whose own levels, sequence and structure may also impact trigger RNA binding. Of note, the 75-nt trigger RNA that activated the paralogous gene *Actg2* (Fig. 3D, F) did not activate the self-gene *Actg1* (fig. S11S). One possible explanation is that *Actg1* may lack an antisense RNA that regulates its own sense transcript at that locus. Supporting this, such 75-nt sequence (mapping to exon 4 of *Actg1*, Fig. 3D) was not identified in the *Actg1* self-TA trigger screen (Fig. 4B). In addition, and as noted earlier, a 187-nt RNA corresponding to the peak of the *Rela* trigger screen failed to induce *Rela*, while smaller fragments appeared effective. It remains unclear whether any of the aforementioned factors, including RNA structure, may have contributed to this outcome. Consistent with a potential role for additional features influencing trigger RNA activity, the two most frequently observed mutations in both the significant and top 200 sequences from the mutagenesis screen bordered the perfect homology region (Fig. 3J). While the 5′ mutation corrected a mismatch (A to C) to match the corresponding *Actg2* base, the 3′ mutation (T to G) did not, suggesting that it may affect RNA function through an alternative mechanism. Furthermore, the magnitude of upregulation observed may depend on factors such as the number of off-target sites for a trigger RNA, the strength of the sense pre-mRNA repression by antisense RNAs under wild-type conditions, or the basal expression level of the targeted gene. This could help explain why the *Actg2* trigger RNA led to stronger upregulation than other trigger–target combinations, given the relatively low expression of *Actg2* under wild-type conditions. Together, understanding how these factors influence trigger RNA activity may help design more effective trigger RNAs. Future studies will be needed to define additional requirements of trigger RNAs, including structural (*175, 176*) or context-dependent features, beyond the sequence homology constraints identified in this study. It is also of interest to determine how any of the factors discussed earlier may influence the extent of ILF3 enrichment at RNAs originating from different adapting gene loci. As illustrated in Fig. 2B, RNAs from several gene loci exhibited stronger ILF3 enrichment than those from *Actg2* in *Actg1*-NSD cells relative to WT. It will also be important to determine mechanisms through which trigger RNAs can activate their cognate targets in an ILF3-independent manner. Similar to ASOs, trigger RNAs may directly bind and interfere with antisense transcripts, thereby enhancing sense gene expression. It is worth exploring whether specific RNA modifications, or better sequence designs, could increase trigger RNA stability and enable them to function fully independently of ILF3.

Fig. S1.

**Validation and characterization of Flow-FISH-based CRISPR screen results.** (**A**) Flow cytometry analysis of signal from *Actg2* FISH probes used in the screen in WT and *Actg1-*NSD cells. (**B**) Gene-level enrichment of the average scores of the sgRNAs in the bottom 30% of *Actg2*/*Rpl13a* expressing cells relative to the top 30% of expressing cells, plotted against MAGeCK-calculated *P* values obtained from a genome-wide CRISPR screen in WT cells. gRNAs targeting *Ilf3* do not decrease *Actg2* expression in WT cells, and those targeting *Actg1* increase *Actg2* further confirming the specificity of the used FISH probes. (**C**) Gene-level enrichment of the average scores of the sgRNAs in the bottom 30% of *Actg2*/*Rpl13a* expressing cells relative to the top 30% of expressing cells, plotted against MAGeCK-calculated *P* values obtained from two independent replicates of a genome-wide CRISPR screen in *Actg1-*NSD cells. Highlighted genes represent a validation of the efficiency of the screen: i) RNA decay factors and the COMPASS complex previously shown to influence TA also identify as hits in the screen ii) gRNAs targeting *Actg2* but not *Actg1* decrease *Actg2* FISH signal confirming the specificity of the used FISH probes. gRNAs targeting *Actb* also lead to increased *Actg2* signal. (**D**) Western blot analysis of ILF3 in *Actg1-*NSD cells and two generated *Ilf3* knockout clones. Upper band is NF110, and lower band is NF90. (**E**) Western blot analysis of ILF3 in WT cells and the generated *Ilf3* knockout clone (WT;∆*Ilf3*). Upper band is NF110, and lower band is NF90. (**F**) qPCR analysis of *Actg1* mRNA expression levels in WT MEFs, WT;∆*Ilf3*, *Actg1*-NSD cells and the two generated *Actg1*-NSD;∆*Ilf3* clones. The decrease in *Actg1* levels in *Actg1*-NSD;∆*Ilf3* clones relative to *Actg1*-NSD cells might be due to TA promoting the expression of the mutant gene too as previously suggested, a phenomenon termed self-TA (*13*). However, this was not observed in the perturb-seq dataset (Fig. 1H), suggesting that these genes may not be subject to strong levels of self-TA. (**G**) Fitted exponential decay curves of *Actg1* mRNA expression levels in *Actg1*-NSD and *Actg1*-NSD;∆*Ilf3* cells at different time points upon transcriptional inhibition with actinomycin D. Data shows that *Actg1* mRNA has a similar half-life in both *Actg1*-NSD and *Actg1*-NSD;∆*Ilf3*, suggesting that loss of ILF3 doesn’t stabilize the *Actg1* mutant mRNA, whose decay was previously established (*13*). t1/2: half-life. (**H**) qPCR quantification of newly synthesized (4sU-labeled) *Actg1* mRNA remaining 7 hours after the end of labeling, relative to the 0-hour time point, in *Actg1*-NSD and *Actg1*-NSD;Δ*Ilf3* MEFs. Cells were pulse-labeled with 4sU for 1 hour followed by a uridine chase to monitor mRNA decay. The similar decrease in labeled *Actg1* mRNA between the two genotypes indicates that loss of ILF3 does not alter *Actg1*-NSD mRNA stability. (**I**) Western blot analysis of co-immunoprecipitation (co-IP) of FLAG-tagged NF110 from MEFs with the indicated proteins. FLAG-tagged LacZ was used as a control. Co-IPs were performed with and without RNase treatment to assess RNA dependence. IP: Immunoprecipitation. p-UPF1: phospho-UPF1 (Ser1127). (**J**) Western blot analysis of ILF3 (top blot) and FLAG (middle blot) in the indicated cell lines. *Actg1*-NSD;∆*Ilf3* cells rescued with NF110∆DZF do not show a signal with the ILF3 antibody as the epitope used to generate the antibody came from the DZF domain. Its expression, however, is apparent with the FLAG antibody. (**K**) Western blot analysis of ILF3 in *Actg1*-NSD and *Actg1*-NSD;∆*Ilf3* cells rescued with FLAG-tagged NF110 expressed under the control of the endogenous *Ilf3* promoter. A dilution series was used to compare expression levels. The blot shows that FLAG-tagged NF110 in *Actg1*-NSD;∆*Ilf3* cells is expressed at levels comparable to the combined levels of endogenous NF110 (upper band) and NF90 (lower band) in *Actg1*-NSD cells. (**L**) qPCR analysis of *Actg2* and *Ilf2* mRNA expression levels in *Actg1-*NSD cells expressing the dCas9-Zim3 CRISPRi machinery and transduced with non-targeting (control) or *Ilf2* gRNAs. (**M**) Western blot analysis of ILF2 (NF45) in *Actg1*-NSD Zim3 dCas9 cells transduced with a control or *Ilf2* gRNA. (F, G, L) n = 3 biologically independent samples. Control expression levels were set at 1 for each assay. Data are mean ± s.d., and a two-tailed Student’s t-test was used to calculate *P* values.

Fig. S2.

**CRISPRn and CRISPRi Perturb-seq nominate candidate TA responses not driven by several tested confounding factors** (**A**) Number of significant differentially expressed genes (DEGs) upon perturbation of a gene by CRISPRn versus CRISPRi. Each dot represents a perturbed gene (n=84). Both CRISPRn and CRISPRi perturbations of a given gene lead to a correlated close number of DEGs. (**B**) Log_2_ Euclidean distance between PCA-transformed transcriptomic responses to perturbations of the same gene versus different genes. Distances were computed in PCA space using the indicated number of principal components. Distances between CRISPRn and CRISPRi perturbations of the same gene (self, blue) are compared to the median distance between CRISPRn perturbation of a gene and CRISPRi perturbations of other (non-self) genes (orange). P-values were calculated using the Mann-Whitney U test. The data show that transcriptomes from CRISPRn and CRISPRi perturbations of the same gene cluster more closely than those from different genes, indicating similarly successful perturbation by both methods and minimal batch effects. n = 84 per violin plot. **C**) Euclidean distance in high dimensional space between transcriptomes upon CRISPRn or CRISPRi perturbation of a given gene (dot in this plot) plotted against expression levels of the perturbed gene relative to control cells when perturbing it with CRISPRi (top) or CRISPRn (bottom). (**D**) Euclidean distance in high dimensional space between transcriptomes upon CRISPRn or CRISPRi perturbation of a given gene (dot in this plot) plotted against the number of identified control (top) and TA-candidate (bottom) observed genes for each perturbed gene. (**C, D**) Data shows that the differences in transcriptome profiles following CRISPRn versus CRISPRi perturbations was independent of the efficiency of NMD or knockdown, respectively, but probably by TA-responses. n=84. (**E**) Number of TA-candidate gene pairs identified for each perturbed gene (dot) plotted against expression levels of the perturbed gene relative to control cells when perturbing it with CRISPRi. Essential genes were identified from the Cancer Dependency Map common essential genes as defined in 20Q1. n=84. (**F**) Expression levels (log_2_[TPM+1]) of the perturbed genes in WT K562s as identified from the Epimap dataset, plotted against the number of identified control gene pairs (left) and TA-candidate (right). n=84. (A, C-F) r value is that of the Pearson correlation.

Fig. S3.

**TA-candidate pairs exhibit properties consistent with previously characterized TA models.** (**A**) Cumulative distribution of the ratio of expression fold change upon perturbing a gene with CRISPRn relative to that upon CRISPRi perturbation. Data shows the values for all genes identified to be significantly upregulated upon CRISPRn-mediated perturbation, and more upregulated in CRISPRn than CRISPRi. Line colors represent the different sets of gene pairs indicated. Blue: all gene pairs that met the previous criteria (n =1873), Orange: all gene pairs exhibiting sequence similarity with E value <1000 regardless of whether they are TA-candidate or not (n=716). Thin lines represent subsets of the thick orange line: Green: all gene pairs where sequence similarity lies in the gene body of the observed (assessed) gene (n=207); Red: all gene pairs where sequence similarity lies in the promoter of the observed (assessed) gene (n=223); Magenta: all gene pairs where sequence similarity lies in a putative enhancer of the observed (assessed) gene (n=563). The data show that genes exhibiting sequence similarity to the mutant gene’s mRNA tend to be upregulated to higher levels upon CRISPRn-mediated perturbation compared to CRISPRi—as reflected in elevated CRISPRn/CRISPRi expression ratios—relative to the distribution observed across all gene pairs. P values are for each dataset in relation to All gene pairs, and were calculated by Mann-Whitney U test. Conclusions don’t change when using the stricter E-value threshold of <1. (**B**) Subsampling analysis showing the distribution of median −log₁₀(E) values from the best BLAST alignment per gene pair, regardless of alignment location (gene body, promoter, or enhancer), from 10,000 randomly drawn subsamples for each comparison set. Each subsample is size-matched to the number of TA gene pairs (754). Gene-pair groups are: Control pairs (blue, n of pairs from which subsamples were drawn=2559), non-TA-candidate pairs where the observed (assessed) genes are in the TA-candidate list (magenta, n of pairs from which subsamples were drawn=52166), all other non-TA-candidate pairs where the observed gene is significantly upregulated (FC > 1, Padj ≤ 0.05) in the WT CRISPRn Perturb-seq dataset (green, n of pairs from which subsamples were drawn=1644), pairs that did not display any change in the WT CRISPRn or CRISPRi Perturb-seq dataset (Pval > 0.05) (orange, n of pairs from which subsamples were drawn=578288), and all gene pairs that aren’t TA pairs (gray, n of pairs from which subsamples were drawn=673996). The red dashed line indicates the median -log₁₀ E-value obtained from the TA-pairs, which shows higher sequence similarity to all compared sets. The p-value is an empirical (ranked) p-value: the fraction of subsamples whose median exceeds the TA-pairs median. Subsampling was used to ensure a fair comparison across groups and avoid set-size–driven differences in E-value distributions. A larger −log_10_(E) indicates higher sequence similarity. (**C)** Distance between PCA components of the gene pairs based of association patterns to health traits from a UK biobank phenotypes exome-wide association results from (*138*) (see Methods) across different number of components. For each gene pair in a set, lower distance is indicative that the two genes have more similar association patterns. Plotted are 681 of the total 754 TA-candidate pairs and 2315 of the total 2559 control pairs. Missing gene pairs are for observed genes that that did not have a reported UK Biobank phenotype association. (**D**) Cumulative distribution of alignment length divided by the length of the aligned-to feature (i.e., gene body, promoter, or the indicated enhancer elements of the observed genes) for TA-candidate (red line) and control (blue line) gene pairs. These data show that TA-candidate pairs tend to have longer normalized alignment lengths than control pairs. All alignments from gene pairs with at least one BLAST hit (alignment) to the tested feature with a negative log₁₀ E-value > 0 were included. Number of plotted alignments for TA-candidate and control gene pairs, respectively, for each feature that met the previous criteria: 46:82 (enhancer_ABC), 31:110 (enhancer_Epimap), 215:317 (enhancer_eRNA-Lidschreiber), 71:100 (enhancer_eRNA-Yulab), 161:416 (promoter_2500bp), 2842:8659 (gene body). These alignments were derived from the following number of TA-candidate and control gene pairs, respectively: 38:145 (genebody), 27:96 (promoter), 22:44 (enhancer_ABC), 15:62 (enhancer_Epimap), 31:75 (enhancer_eRNA-Lidschreiber), and 24:56 (enhancer_eRNA-Yulab). Some gene pairs exhibited sequence similarity to multiple features (e.g., both a gene body and an enhancer element). Each of these homologies was considered separately in this analysis. (**E**) Cumulative distribution of negative log_10_ E-values from the best BLAST alignment per gene pair, for TA-candidate gene pairs (red line) or Control gene pairs (blue line) where the alignment to the observed gene lies in a putative enhancer region. Gene pairs were included only if they had at least one BLAST alignment to the tested enhancer elements with a negative log_10_ E-value > 0. A higher -log_10_ E-value indicates higher level of similarity. Numbers of plotted TA-candidate, and control gene pairs, respectively, that met the previous criteria for each enhancer set: 22:44 (enhancer_ABC), 15:62 (enhancer_Epimap), 31:75 (enhancer_eRNA-Lidschreiber), and 24:56 (enhancer_eRNA-Yulab). (**D, E**) for gene pairs where similarity lies in a putative enhancer region of the observed gene, alignments to enhancers where evidence of enhancer RNA (eRNA) transcription was present displayed higher levels of similarity and longer lengths in TA-candidate pairs relative to Control pairs, which was not always the case for enhancers without direct evidence of eRNAs. These data suggested that eRNAs could be a target for mRNA decay intermediates. (**F**) Cumulative distribution of the alignment start position relative to gene body length for TA-candidate (red line) and control (blue line) gene pairs. These data indicate that the position of alignment between the perturbed gene’s mRNA and the adapting gene’s body has limited influence on TA—that is, whether the alignment lies toward the 5′ or 3′ end of the adapting gene appears to have minimal effect. Number of alignments plotted: 2,842 (TA-candidate) and 8,659 (control). All alignments from gene pairs with at least one BLAST hit to the gene body of the observed gene, with a negative log₁₀ E-value > 0, were included. These alignments were derived from 38 TA-candidate gene pairs and 145 control gene pairs, respectively. (A, C-F) *P* values were calculated by Mann-Whitney U test.

Fig. S4.

**ILF3 as a mediator of TA.** (**A**) Western blot analysis of ILF3 in wild-type K562-Cas9 cells and in *ILF3* polyclonal knockout cells, generated by transducing K562-Cas9 cells with a vector co-expressing Cas12 and four *ILF3*-targeting gRNAs and harvested eight days post-transduction. (**B**) Number of significant differentially expressed genes (DEGs) upon perturbation of a gene by CRISPRn in WT vs *ILF3* KO conditions. Each dot represents a perturbed gene. CRISPRn perturbations of a given gene lead to a correlated close number of DEGs in WT or *ILF3* KO cells. n=84. r value is that of Pearson correlation. (**C**) Log_2_ Euclidean distance between PCA-transformed transcriptomic responses to CRISPRn perturbations of the same gene versus different genes. Distances were computed in PCA space using the indicated number of principal components. Distances between CRISPRn perturbations of the same gene (self, blue) in WT and *ILF3* KO cells are compared to the median distance between CRISPRn perturbation of a gene in WT cells and perturbations of other (non-self) genes in *ILF3* KO cells (orange). P-values were calculated using the Mann-Whitney U test. The data show that perturbations of the same gene yield more similar transcriptomic profiles than those of different genes, consistent with successful gene perturbation and minimal batch effects. n = 84 per group. (**D**) Relative cell counts of K562-Cas9 cells transduced with the Cas12 *ILF3* knockout (KO) vector compared to K562-Cas9 cells transduced with a non-targeting sgRNA, measured at the indicated days post-transduction. Equal numbers of cells were seeded 1–2 days prior to counting. Also shown are WT K562-Cas9 cells grown under low-serum (2% FBS) conditions relative to those grown under standard (10% FBS) conditions, and WT;∆*Ilf3* MEFs relative to WT MEFs. Data represent mean ± s.d.; *P* values were calculated using a two-tailed Student’s t-test. (**E**) Cumulative distribution of E-values obtained upon BLASTing ILF3 motifs (identified from eCLIP-seq ENCODE data (*53, 54*)) with the alignment region from the perturbed-gene to the assessed (observed) gene within TA-candidate (red line) or Control (blue line) gene pairs. Only gene pairs found to have at least one high-confidence match (motif matches with a *P* value < 0.0001) to an ILF3 motif were included for each pair type. n= 121 of the total 754 TA-candidate gene pairs, and 220 of the total 2559 control pairs. (**F**) Percentage of gene pairs with a BLAST alignment between the perturbed and observed gene with E-value <1000, where the aligned region in the perturbed gene contains a high-confidence match (P < 0.0001) to an ILF3 motif identified from ENCODE eCLIP-seq data. For TA-candidate pairs: 121 out of 475 gene pairs with an alignment (from a total of 754). For control pairs: 220 out of 1,660 gene pairs with an alignment (from a total of 2559). A more relaxed BLAST E-value threshold was used here—unlike Fig. S3, which applied a stricter E-value cutoff of <1—in order to increase the number of alignment regions that could be evaluated for ILF3 motif presence. To offset the increased sensitivity, a more stringent P-value threshold for ILF3 motif matches (<0.0001) was applied to reduce noise and improve confidence in motif assignments. (**G**) Cumulative distribution of the observed (adapting) genes’ expression fold change in the CRISPRn perturb-seq dataset. The two lines are TA-candidate pairs where the perturbed gene’s alignment region to the observed (adapting) gene has a high-confidence match to an ILF3 motif (orange line, n=121) versus gene pairs where the alignment region does not match any ILF3 motif, even without applying a P-value threshold (teal line, n = 78). Notably, the conclusions remain consistent when comparing gene pairs with a high-confidence ILF3 motif match to all other TA-candidate pairs not meeting this criterion—including those with no detectable sequence similarity (and thus no alignment region to assess), those with alignments that do not match any ILF3 motif, and those with alignments containing only low-confidence ILF3 motif matches (P > 0.0001). (C, E, G) *P* values were calculated by Mann-Whitney U test.

Fig. S5.

**Validation experiments for *DDX21* and *CSNK1E*.** (**A**) Western blot analysis of UPF1 in K562-Cas9 cells expressing non-targeting (control) or *DDX21* or *CSNK1E* sgRNAs WT cells or *UPF1* polyclonal KO cells. (**B**) Western blot analysis of ILF3 in K562-Cas9 cells expressing non-targeting (control) or *DDX21* or *CSNK1E* sgRNAs in WT cells or *ILF3* polyclonal KO cells. (**C**) qPCR analysis of the indicated genes’ mRNA expression levels in K562-Cas9 cells that express a non-targeting (control) sgRNA in WT, *UPF1* KO or *ILF3* KO conditions. (**D**) qPCR analysis of the indicated genes’ mRNA expression levels in K562-Cas9 cells that express a non-targeting (control) sgRNA in WT (ctrl), *UPF1* KO or *ILF3* KO conditions. (**E**) qPCR analysis of *DDX21* mRNA expression levels in K562-Cas9 cells that express a non-targeting (control), or *DDX21* sgRNA in WT, *UPF1* KO and *ILF3* KO conditions. (**F**) qPCR analysis of *CSNK1E* mRNA expression levels in K562-Cas9 cells that express a non-targeting (control), or *CSNK1E* sgRNA in WT, *UPF1* KO and *ILF3* KO conditions. (**G**) Fitted exponential decay curves of *DDX21* mRNA expression levels K562-Cas9 cells that express a non-targeting (control), or *DDX21* sgRNA in WT and *ILF3* KO conditions. t1/2: half-life. Student’s t-test was used to calculate *P* values. (**H**) Fitted exponential decay curves of *CSNK1E* mRNA expression levels K562-Cas9 cells that express a non-targeting (control), or *CSNK1E* sgRNA in WT and *ILF3* KO conditions. t1/2: half-life. Student’s t-test was used to calculate *P* values. (C-F) n = 3 biologically independent samples. Control expression levels were set at 1 for each assay. Data are mean ± s.d., and a two-tailed Student’s t-test was used to calculate *P* values.

Fig. S6.

**Reduced proliferation independent of ILF3 loss does not abolish upregulation of *DDX21* TA-candidate adapting genes.** (**A**) Western blot analysis SRP9 in K562-Cas9 cells expressing non-targeting (control) or *DDX21* sgRNAs WT cells or *SRP9* polyclonal KO cells. (**B**) Western blot analysis SERBP1 in K562-Cas9 cells expressing non-targeting (control) or *DDX21* sgRNAs WT cells or *SERBP1* polyclonal KO cells. (**C**) qPCR analysis of the indicated genes’ mRNA expression levels in WT K562-Cas9 cells grown in proliferation-limiting 2%FBS conditions or Cas12-generated-*SRP9* or *SERBP1* polyclonal KO K562-Cas9 cells transduced with either a non-targeting (control), or *DDX21* sgRNA. (**D**) qPCR analysis of the indicated genes’ mRNA expression levels in K562-Cas9 cells that express a non-targeting (control) sgRNA in WT cells grown in proliferation-limiting 2%FBS conditions, *SRP9* KO or *SERBP1* KO conditions relative to WT cells grown in standard 10%FBS conditions. (**E**) qPCR analysis of *DDX21* mRNA expression levels in K562-Cas9 cells that express a non-targeting (control), or *DDX21* sgRNA in WT cells grown in 10%FBS or 2%FBS, *SRP9* KO and *SERBP1* KO conditions. (C-E) n = 3 biologically independent samples. Control expression levels were set at 1 for each assay. Data are mean ± s.d., and a two-tailed Student’s t-test was used to calculate *P* values.

Fig. S7.

**Disruption of RNA:DNA hybrids does not affect transcriptional adaptation.** (**A**) qPCR analysis of mRNA expression levels of the indicated adapting genes in their respective knockout cell lines overexpressing RNASEH1, relative to knockout cells without RNASEH1 overexpression. Control expression levels were set at 1 for each assay. (**B**) Top: Western blot analysis of RNASEH1 expression in *Actg1*-NSD and K562-Cas9 cells overexpressing RNASEH1. The RNASEH1 signal in the control (Ctrl) lanes appears absent due to signal saturation from the strong overexpression in the adjacent lane, which makes the control signal appear comparatively undetectable. Bottom: When control samples are run separately, a longer exposure reveals that RNASEH1 is clearly detectable, confirming that the apparent absence of signal in the top panel is an artifact of overexposure rather than a lack of expression. (**C**) log₂[TPM + 1] of antisense reads from PRO-seq data in WT MEFs (this study; n = 14648 genes, of which only 261 had a TPM of zero) and K562 cells (data from (*52*); n = 15178 genes, 5885 of which were detected in our Perturb-seq dataset, with only 9 having an antisense TPM of zero). Only genes with detectable sense reads in PRO-seq are included. These data indicate that most genes exhibit bidirectional transcription. *Actg1* and *Actg2* are highlighted in the MEF dataset. The entire genebody (including introns) was considered when calculating TPM. (**D**) Log_2_ fold change of RNAs’ IP/input signal in the ILF3 nuclear RIP-seq experiments in *Actg1*-NSD cells relative to that in WT MEFs as identified by an unstranded analysis (left), an antisense-specific analysis (middle), or sense-specific analysis (right). Plotted genes are those identified to be enriched (log_2_ (fold change)≥1 and *P*_val_ ≤0.01) according to the unstranded analysis that are presented in Fig. 2B. *Actg1* and *Actg2* are both highlighted. The strand-specific analysis shows that most genes enriched in the unstranded data are also enriched when considering only the antisense or sense strand, supporting the rationale for using the unstranded analysis for identification of ILF3-enriched genes. (**E**) Subsampling analysis showing the distribution of median -log₁₀ E-values from 10,000 randomly sampled gene sets (n = 383 genes per set), matched in number to the genes enriched for ILF3 binding in *Actg1*-NSD versus WT MEFs (as identified in Fig. 2B). Each set was generated by randomly selecting genes from the full set of annotated genes that showed no change in ILF3 recruitment between *Actg1*-NSD and WT (Pval>0.05, baseMean >30; n=14274), and aligning their sequences to *Actg1* mRNA using BLAST. The x-axis shows the median -log₁₀ E-value for each random gene set. The red dashed line indicates the median -log₁₀ E-value obtained from the ILF3-enriched genes, which shows higher sequence similarity to *Actg1* mRNA than most randomly selected gene sets. P-value is an empirical (ranked) p-value. Subsampling was performed to enable a fair comparison, since directly comparing the ILF3-enriched gene set (n = 383) to the full gene set could artificially inflate differences due the particularly large disparity in set size. (**F**) Gene Ontology (GO) term enrichment analysis (*177*) for genes enriched for ILF3 binding in *Actg1*-NSD versus WT MEFs (as identified in Fig. 2B). The top ten terms based on *P* value are displayed. The dashed line marks a *P* value of 0.01. Marked in red are terms that are connected to ACTG1 function. (**G**) Cross-linked nuclear RIP-qPCR analysis of ILF3 recruitment to the indicated genes in K562-Cas9 cells expressing non-targeting (control) or *DDX21* sgRNAs. (**H**) Gene-level enrichment of the average scores of the sgRNAs in the bottom 30% of *Actg2*/*Rpl13a* expressing cells relative to the top 30% of expressing cells, plotted against MAGeCK-calculated *P* values obtained from two independent replicates of a genome-wide CRISPR screen in *Actg1-*NSD cells. Genes scoring as hits in the counter screen in WT cells were removed prior to the MAGeCK analysis, and hence the figure looks different than fig. S1C. PolII-pausing promoting factors are marked in blue while those promoting transcription (Tx) elongation are marked in orange. (A, G) Data are mean ± s.d., and a two-tailed Student’s t-test was used to calculate *P* values.

Fig. S8.

**ILF3-dependent enrichment of epigenetic modifiers in transcriptional adaptation.** (**A**) Gene-level enrichment of the average scores of the sgRNAs in the bottom 30% of *Actg2*/*Rpl13a* expressing cells relative to the top 30% of expressing cells, plotted against MAGeCK-calculated *P* values obtained from two independent replicates of a genome-wide CRISPR screen in *Actg1-*NSD cells. Genes scoring as hits in the counter screen in WT cells were removed prior to the MAGeCK analysis, and hence the figure looks different than fig. S1C. Highlighted is *Smarca4* (BRG1) a core component of the SWI/SNF chromatin remodeling complex. (**B**) ChIP-qPCR analysis of BRG1, PRMT1 and YY1 at the *Actg2* locus or *Rel* (as a control locus), in WT, *Actg1*-NSD, *Actg1*-NSD;∆*Ilf3* and WT;∆*Ilf3* cells. The *Actg2* and *Rel* loci are shown below the plot, with the position of a primer pair at the TSS shown (purple triangles). Data are mean ± s.d., and a two-tailed Student’s t-test was used to calculate *P* values.

Fig. S9.

**Artificial recruitment of ILF3 activates gene expression.** (**A**) Western blot analysis dCas13 from nuclear lysates of WT MEFs expressing dCas13 or dCas13-NF110 or dCas13-lacZ. Red star marks the expected dCas13 band. YY1 was used as a loading control. As previously reported for fusion proteins with Cas9 (*178*), we observed a destabilization of the dCas13-NF110 protein relative to dCas13 alone, and hence included a dCas13-lacZ control that is expressed at similar levels. (**B**) ChIP-qPCR analysis of WDR5, H3K4me3, BRG1, PRMT1 and YY1 at the *Actg2* locus or *Rel* (as a control locus) in WT MEFs expressing dCas13-NF110 fusion protein with a non-targeting (control) gRNA, or a gRNA targeting antisense of *Actg2*. The *Actg2* and *Rel* loci are shown below the plot, with the position of a primer pair at the TSS shown (purple triangles). AS: antisense. (**C**) ChIP-qPCR analysis of WDR5, H3K4me3, BRG1, PRMT1 and YY1 at the *Actg2* locus in WT MEFs expressing dCas13-lacZ fusion protein with a non-targeting (control) gRNA, or a gRNA targeting antisense of *Actg2*. AS: antisense. (**D**) qPCR analysis of *Cdk9* or *Rel* mRNA expression levels upon transducing cells expressing dCas13-NF110 fusion protein (white bars) or dCas13 alone (grey bars) or dCas13-lacZ (bars with stripes) with a non-targeting (control) gRNA, or gRNAs targeting antisense (A.S.) of *Cdk9* or *Rel*. ex: exon; intr: intron. (**E**) qPCR analysis of *Actg2* mRNA expression levels upon transducing cells expressing dCas13-NF110 fusion protein (white bars) or dCas13 alone (grey bars) or dCas13-lacZ (bars with stripes) with a non-targeting (control) gRNA, or gRNAs targeting the non-coding exon 1 of *Actg2* sense pre-mRNA. The higher upregulation levels obtained relative to some gRNAs targeting *Actg2* antisense RNA is probably due to the higher expression levels of *Actg2* sense pre-mRNA relative to the antisense RNA, which could enable more recruitment of dCas13-NF110. ex: exon. (**E**) qPCR analysis of *Serpine1* or *Nrg1* mRNA expression levels upon transducing cells expressing dCas13-NF110 fusion protein (white bars) or dCas13 alone (grey bars) or dCas13-lacZ (bars with stripes) with a non-targeting (control) gRNA, or gRNAs targeting *Serpine1* or *Nrg1* sense premRNA. ex: exon; intr: intron. (D-F) n = 3 biologically independent samples. Control expression levels were set at 1 for each assay. (B-F) Data are mean ± s.d., and a two-tailed Student’s t-test was used to calculate *P* values.

Fig. S10.

**Trigger screens enable identification of the trigger components of TA.** (**A**) Cartoon depicting how NMD vectors can induce TA. GOI: Gene of interest. PTC: Premature Termination Codon. EJC: Exon Junction Complex. The NMD vector was designed to include two premature termination vectors followed by exons and introns of *HBB* which was reported to be a robust model of NMD (*179*). The presence of introns is required for EJC deposition upon splicing and subsequent efficient NMD (*9*). (**B**) qPCR analysis of the transduced transgene expression levels in WT MEFs transduced with a vector expressing *Actg1* in an NMD vector relative to *Actg1* in a vector without *HBB* exons and introns (No-NMD). (**C**) qPCR analysis of *Actg2* and the endogenous *Actg1* mRNA expression levels in WT MEFs expressing *Actg1* coding sequence from the No-NMD vector or control, GFP-2A-RFP, from the NMD vector. (**D**) RNAseq analysis of WT MEFs expressing *Actg1* from the NMD vector relative to control GFP-2A-RFP. Data shows that *Actg2* is within the top most upregulated genes. (**E**) Location of the significant triggers identified from the trigger screen mapped onto *Actg2* mRNA sequence. White gaps in the arrows indicate a mismatch. Sequences below shows that the identified *Actg1* 75 nucleotide trigger shares extensive homology with a 75-nucleotide region in *Actg2*, in addition to the 24, 27 and 31 nucleotide trigger RNAs used in Fig. 3F and fig. S10I. Red strokes indicate a mismatch. The perfect homology region represents a stretch of 20 nucleotides within the *Actg1* 75 nucleotide sequence that is exactly similar to *Actg2*. The 3′ end of the RNA, contains multiple mismatches and is referred to as the imperfect homology region (**F**) qPCR analysis of *Actg2* mRNA expression levels in WT MEFs expressing the trigger-screen-identified 75-nucleotide trigger region, or *Actg1* without the 75-nucleotide trigger region in the NMD vector relative to control GFP-2A-RFP. (**G**) qPCR analysis of *Actg2* and *Actg1* pre-mRNA and mRNA expression levels in *Actg1*-NSD∆75 cells (*Actg1*-NSD MEFs where exon 4 of *Actg1* containing the 75-nt trigger sequence was deleted) relative to *Actg1*-NSD cells. Decreased transcription (pre-mRNA) was only observed for *Actg2*, but not *Actg1*, in agreement with that this trigger region was identified only for *Actg2* (Fig. 3D) but not *Actg1* (Fig. 4B). The decreased *Actg1* mRNA levels is possibly due to destabilization of the mRNA as a result of the deleted region. (**H**) qPCR analysis of *Actg2* mRNA expression levels upon transducing cells expressing dCas13-NF110 fusion protein (white bars) or dCas13 alone (grey bars) or dCas13-lacZ (bars with stripes) with a non-targeting (control) gRNA, or gRNAs targeting antisense RNA transcribed from the region corresponding to the 75-nucleotide trigger in *Actg2*. AS: antisense (**I**) qPCR analysis of *Actg2* mRNA expression levels in WT MEFs transfected with the indicated trigger RNAs. The 24, 27, 31 nt RNAs induced a significant mild upregulation of *Actg2* and thereafter guided the co-transfection experiment in Fig. 3F. The control used for the 21-31 nt trigger RNA experiments is the same as that used in the 24+27+31 experiment in WT MEFs in Fig. 3F. (**J**) Log₁₀ of the median 6-FAM intensity observed upon nucleofecting MEFs with 6-FAM-conjugated versions of the ASOs used in Fig. 3G, plotted against the median upregulation levels of *Actg2* relative to control. 6-FAM intensities for mock-nucleofected cells and those transfected with control ASOs are shown below. The plot demonstrates efficient nucleofection of all ASOs and reveals no correlation between nucleofection efficiency (measured by 6-FAM intensity) and *Actg2* upregulation, suggesting that differences in gene induction likely reflect variation in the ASOs' ability to knock down the targeted antisense RNA. Different off-target effects of the individual ASOs that indirectly affect gene induction levels, however, cannot also be ruled out. r value is that of Pearson correlation. (**K**) qPCR analysis of *Actg2* mRNA expression levels in *Ilf3* knockdown MEFs transfected with the indicated antisense oligos (ASOs) targeting antisense RNAs in the *Actg2* locus homologous to the 75-nucleotide identified from the trigger screen relative to non-targeting control. (**L**) Western blot analysis of RNASEH1in WT Cas9 cells transduced with a control or *Rnaseh1* gRNA. (**M**) qPCR analysis of *Actg2* mRNA expression levels in *Rnaseh1* knockout MEFs transfected with the indicated antisense oligos (ASOs) targeting antisense RNAs in the *Actg2* locus homologous to the 75-nucleotide identified from the trigger screen relative to non-targeting control. (**N**) qPCR analysis of *Actg2* mRNA expression levels in WT MEFs transfected with the 75-nt WT trigger RNA or with variants of it containing non-adjacent 3 or 4 mismatches within either the perfect homology or imperfect homology regions, relative to control. For the imperfect homology region, mutations targeted nucleotides shared between *Actg1* and *Actg2*; the four-mismatch variant included one mutation in a non-homologous nucleotide. (A) Figure was created with BioRender.com. (E) Figure made in Snapgene. (B, C, F-I, K, M, N) n = 3 biologically independent samples. Control expression levels were set at 1 for each assay. Data are mean ± s.d., and a two-tailed Student’s t-test was used to calculate *P* values.

Fig. S11.

**TA trigger screens highlight candidate sequences for same-gene activation.** (**A**) Images of gel electrophoresis following PCR of WT MEFs or the homozygous NG11-*Actg1* knockin (KI) KI using primers flanking the knockin region. To obtain the endogenous ACTG1 reporter cell line, *NG11* sequence was inserted immediately downstream of the *Actg1* start codon, while stably expressing *NG1-10* from a separate transgene. (**B**) qPCR analysis of *Actg1* mRNA expression levels in WT MEFs transfected with the indicated RNAs identified from a native nuclear ILF3 RIP-seq experiment to be originating from TR2 relative to mock transfected control. Two different 40-nt RNAs where tested and were denoted as (a) or (b). (**C**) qPCR analysis of *Actg1* mRNA expression levels upon transducing cells expressing dCas13-NF110 fusion protein (white bars) or dCas13 alone (grey bars) or dCas13-lacZ (bars with stripes) with a non-targeting (control) gRNA, or gRNAs targeting antisense RNA transcribed from the indicated *Actg1* trigger regions. AS: antisense. (**D**) qPCR analysis of *Rela* expression levels in WT (white bars) or *ILF3* knockdown (kd) (grey bars) MEFs expressing the *Rela* coding sequence, or a control (GFP-2A-RFP) message from the NMD vector. (**E**) qPCR analysis of the endogenous *Rela* mRNA expression levels in WT MEFs expressing *Rela* coding sequence from the No-NMD vector or control, GFP-2A-RFP, from the no-NMD vector. (**F**) Flow cytometry analysis of signal from *Rela* FISH probes used in the *Rela* trigger screen in WT and *Rela* knockdown MEFs. (**G**) qPCR analysis of *Rela* mRNA expression levels in WT and *Rela* knockdown MEFs. (**H**) Enrichment of triggers in the top 10% of *Rela/Rpl13a* expressing cells, relative to the bottom 10% of expressing cells, plotted against one-sided MAGeCK-calculated *P* values obtained from two independent replicates of the trigger screen. Red dots represent triggers that significantly increased *PKD1* expression (Log_2_ Fold Change ≥0.5, P_value_ ≤0.01), while blue dots represent the control random scrambled triggers showing that none of them scored as significant. (**I**) Location of the significant triggers identified from the trigger screen mapped to *Rela* coding sequence. A single trigger region of 187-nt was identified. Figure made in Snapgene. (**J**) qPCR analysis of *Rela* mRNA expression levels in WT MEFs transfected with the 187-nt RNA identified from the trigger screen relative to mock transfected control. (**K**) qPCR analysis of *Rela* mRNA expression levels in WT (white bars) or *Ilf3* knockdown (grey bars) MEFs transfected with the indicated RNAs relative to mock transfected control. The asterisk sign (*) marks RNAs that successfully induced *Rela* expression, and which were then tested for ILF3-dependency. Because the native ILF3 RIP-seq was performed in *Actg1*-NSD cells lacking an mRNA-destabilizing *Rela* mutation, the identified RNAs may be derived from normal decay of the wild-type *Rela* transcript. (**L)** qPCR analysis of *Rela* mRNA expression levels upon transducing cells expressing dCas13-NF110 fusion protein (white bars) or dCas13 alone (grey bars) or dCas13-lacZ (bars with stripes) with a non-targeting (control) gRNA, or gRNAs targeting antisense RNA transcribed from the identified *Rela* trigger region. AS: antisense. (**M**) qPCR analysis of *Rela* mRNA expression levels in MEFs nucleofected with ASOs targeting antisense RNAs at the identified *Rela* trigger region. (**N**) qPCR analysis of *PKD1* expression levels in WT HEKs expressing the different segments of the *PKD1* coding sequence (divided into five segments), or a control (GFP-2A-RFP) message from the NMD vector. (**O**) Western Blot analysis of ILF3 in WT and ILF3 knockdown HEK cells. (**P**) qPCR analysis of the *PKD1* mRNA expression levels in WT HEK cells expressing the fifth segment of *PKD1* coding sequence from the No-NMD vector or control, GFP-2A-RFP, from the no-NMD vector. (**Q**) Flow cytometry analysis of signal from *PKD1* FISH probes used in the *PKD1* trigger screen in WT and *PKD1* knockdown HEKs. (**R**) qPCR analysis of *PKD1* mRNA expression levels in WT and *PKD1* knockdown HEKs. (**S**) qPCR analysis of the indicated genes’ mRNA expression levels in WT MEFs or HEKs transfected with the indicated trigger RNAs. The decrease of *Actg1* observed with the *Actg2* 75-nt trigger RNA was not observed on the protein level. (B-E, G, J-N, P, R, S) n = 3 biologically independent samples. Control expression levels were set at 1 for each assay. Data are mean ± s.d., and a two-tailed Student’s t-test was used to calculate *P* values.

Fig. S12.

**Working model for adapting genes where sequence homology is not linked to an antisense RNA.** ILF3 binds mRNA decay intermediates and returns to the nucleus, where it may be guided to the adapting genes’ locus through interactions with regulatory RNAs, such as enhancer RNAs (eRNAs) or sense pre-mRNAs, if they exhibit sequence homology. Once ILF3 is localized at a locus, it may promote gene expression by recruiting transcriptional regulators and/or chromatin modifiers, by enhancing transcriptional elongation, or through a combination of these and other mechanisms. Figure created with BioRender.com.

**Table S1.**

Sequences of Cas9, Cas12 and Cas13 gRNA used in the study.

**Table S2.**

Sequences of qPCR primers used in the study.

**Table S3.**

Results from the CRISPR screens performed in the study.

**Table S4.**

Results of the trigger screen, and sequences of oligos used.

**Table S5.**

Sequences of ASOs and trigger RNAs used in the study.

**Table S6.**

Sequences of the gRNAs used in the CRISPRn Perturb-seq experiment.
